# Comparison of doublet and triplet therapies for metastatic hormone-sensitive prostate cancer: A systematic review and network meta-analysis

**DOI:** 10.3389/fonc.2023.1104242

**Published:** 2023-03-07

**Authors:** Lei Wang, Chunxing Li, Zichen Zhao, Xiaojian Li, Chong Tang, Zhenpeng Guan, Feng Sun, Jin Gu, Ningchen Li

**Affiliations:** ^1^ Wu Jieping Urology Center, Peking University Shougang Hospital, Beijing, China; ^2^ Department of Pharmacy, Aerospace Center Hospital, Beijing, China; ^3^ Department of Orthopedics, Peking University Shougang Hospital, Beijing, China; ^4^ Department of Epidemiology and Biostatistics, School of Public Health, Peking University Health Science Center, Beijing, China; ^5^ Gastrointestinal Cancer Center, Peking University Cancer Hospital, Key Laboratory of Carcinogenesis and Translational Research (Ministry of Education), Beijing, China; ^6^ Department of Gastrointestinal Surgery, Peking University Shougang Hospital, Beijing, China

**Keywords:** prostate cancer, chemotherapy, hormonal therapy, radiation therapy, combination therapy

## Abstract

**Background:**

The best choice of first-line treatment for metastatic hormone-sensitive prostate cancer (mHSPC) is unclear. We aimed to compare the effectiveness and safety determined in randomized clinical trials of doublet and triplet treatments for mHSPC.

**Methods:**

Medline, Embase, Cochrane Central and ClinicalTrials.gov were searched from inception through July 01, 2022. Eligible studies were phase III randomized clinical trials evaluating androgen deprivation treatment (ADT) alone, doublet therapies [ADT combined with docetaxel (DOC), novel hormonal agents (NHAs), or radiotherapy (RT)], or triplet therapies (NHA+DOC+ADT) as first-line treatments for mHSPC. Outcomes of interest included overall survival (OS), progression-free survival (PFS) and grades 3-5 adverse events (AEs). Subgroup analyses were performed based on tumor burden. The effects of competing treatments were assessed by Bayesian network meta-analysis using R software.

**Results:**

Ten trials with 12,298 patients comparing nine treatments were included. Darolutamide (DARO) +DOC+ADT ranked best in terms of OS benefits (OR 0·52 [95% CI 0·39–0·70]), but its advantages were all statistically insignificant compared with other therapy options except for DOC+ADT (OR 0·68 [95% CI 0·53–0·88]) and RT+ADT (OR 0·57 [95% CI 0·40–0·80]). In terms of PFS, enzalutamide(ENZA)+DOC+ADT (OR 0·32 [95% CI 0·24–0·44]) and abiraterone and prednisone (AAP) +DOC+ADT (OR 0·33 [95% CI 0·25–0·45]) ranked best. For patients with high volume disease (HVD), low volume disease (LVD), and visceral metastases, the optimal therapies were AAP+DOC+ADT (OR 0·52 [95% CI 0·33–0·83]), apalutamide+ADT (OR 0·52 [95% CI 0·26–1·05]) and DARO+DOC+ADT (OR 0·42 [95% CI 0·13–1·34]), respectively. For safety, AAP+DOC+ADT (OR 3·56 [95% CI 1·51–8·43]) ranked worst with the highest risk of grade 3−5 AEs.

**Conclusions:**

Triple therapies may further improve OS and PFS but may be associated with a decrease in safety. Triplet therapies could be suggested for HVD patients, while doublet combinations should still be preferred for LVD patients.

**Systematic Review Registration:**

https://www.crd.york.ac.uk/PROSPEROFILES/303117_STRATEGY_20220202.pdf, identifier CRD4202303117.

## Introduction

Prostate cancer (PCa) is one of the most common malignant tumors in men worldwide (1). Distant metastasis often indicates a poor prognosis (2). For metastatic hormone-sensitive prostate cancer (mHSPC), androgen-deprivation therapy (ADT) has been regarded as the standard of care (SOC) and the only systematic treatment option for a long time (3). However, patients receiving ADT will inevitably gradually resist the treatment and enter the stage of castration resistance (mCRPC), which will significantly worsen the prognosis (4–6). Delaying the progression of metastatic prostate cancer to mCRPC has always been an important topic in the field of prostate cancer treatment.

In recent years, the emergence of many novel therapies has greatly improved the prognosis of patients with mHSPC (7–9). These therapies consist of ADT combined with another therapeutic agent. The earlier agent was the docetaxel (DOC) chemotherapy (10–12), followed by the androgen synthesis inhibitor abiraterone acetate (13, 14) and the new androgen receptor inhibitors enzalutamide (ENZA) (15, 16) and apalutamide (APA) (17). The above three drugs and darolutamide (DARO) are also known as novel hormonal agents (NHAs) to distinguish them from traditional endocrine therapeutic drugs such as goserelin and bicalutamide. In addition, ADT combined with radiotherapy (RT) (18) is considered to provide survival benefits for mHSPC patients with low volume disease (LVD). The emergence of these novel combination therapies is significantly changing the previous standard of care for mHSPC using ADT alone, but the advantages and disadvantages of these combination therapies are controversial due to the lack of head-to-head comparisons (4, 7, 8, 19–24).

Recent reports of triplet therapies for mHSPC have made the situation even more confusing. The earliest reports of triplet therapy can be traced back to a subgroup analysis reported in the ENZAMET study in 2019 (15), where up to 44.7% of enrolled patients received docetaxel chemotherapy just before or concurrently with the experimental intervention. Data from this subgroup could be used to analyze the efficacy of ENZA+DOC+ADT versus DOC+ADT, but there was no significant difference in the final overall survival (OS) results. However, the PEACE-1 study (25) published positive results on triplet therapy in 2021, and OS in patients receiving abiraterone combined with DOC+ADT was significantly better than that in patients receiving DOC+ADT. The results from another well-designed phase III clinical study, ARASENS (26), further showed that patients who received the novel hormonal agent darolutamide in combination with DOC+ADT also had a longer OS than patients receiving DOC+ADT.

The emergence of new effective treatment options is beneficial, but for clinical oncologists, understanding the pros and cons of different treatment options is clinically important. This study aimed to conduct a network meta-analysis (NMA) of the efficacy and safety of doublet and triplet therapies for mHSPC identified in phase III randomized clinical trials (RCTs) in recent years to compare the efficacy and safety of different treatments in mHSPC according to the current data.

## Methods

The study protocol was registered with the International Prospective Register of Systematic Reviews (PROSPERO), registration number CRD4202303117.

### Search strategy

According to the Preferred Reporting Items for Systematic Reviews and Meta-Analyses (PRISMA) specification, a systematic search was conducted for RCT studies published prior to and including July 01, 2022. Databases searched included Medline, Embase, Cochrane Central, and ClinicalTrials.gov. The main search terms included prostate cancer, hormone sensitive, clinical trial, docetaxel, abiraterone, enzalutamide, apalutamide, darolutamide, radiotherapy, etc. Different combinations and strategies were applied during different database retrievals (**Appendix P 1-4**). Only the results of RCTs published in English were included in this article, and a search was performed before the final statistical analysis to avoid omitting the latest published results.

### Study selection

The literature was screened according to the principles of Participants, Interventions, Comparisons and Outcomes (PICO). Inclusion criteria were as follows: study population: 1) Adult patients with mHSPC, aged ≥18 years; 2) Patients with initial onset or those who progressed after previous local therapy; 3) The duration of ADT treatment allowed in the stage of localized prostate cancer was no more than three years, and in the stage of metastatic prostate cancer was no more than six months; 4) Data from phase III RCTs. Interventions: 1) First-line treatments for mHSPC recommended in the latest guidelines of EAU and NCCN, including ADT combined with docetaxel, abiraterone acetate, enzalutamide, apalutamide, and RT+ADT (for LVD patients); 2) Recently reported triplet therapies, mainly NHAs+DOC+ADT. Control group: 1) According to the design of different studies, treatments in control groups included ADT alone, ADT plus placebo, or ADT plus traditional nonsteroidal antiandrogens (NSAA) (27, 28); 2) In studies with triplet therapies, the treatment in control groups was DOC+ADT. Outcomes: The main outcome was OS, and the secondary outcomes were progression-free survival (PFS) and safety indicators of high-grade AEs. Exclusion criteria: 1) Patients with metastatic or nonmetastatic castration- resistant prostate cancer; 2) Nonrandomized controlled studies or phase I/II RCTs; 3) Research that could not extract relevant data; 4) Duplicate studies.

### Risk of bias assessment

The methodological quality of each study was independently assessed by two independent investigators using the revised Cochrane Collaboration Risk of Bias tool RoB1.0, and differences of opinion were resolved by consultation. The overall bias of the trial was assessed for each study from the following seven domains: random sequence generation (selection bias), allocation concealment (selection bias), blinding of participants and personnel (performance bias), blinding of outcome assessment (detection bias), incomplete outcome data (attrition bias), selective reporting (reporting bias), and other biases. The study as a whole was deemed as having a “low risk of bias” if all terms were at low risk of bias. The study as a whole was judged to be at high risk of bias if either item was judged to be at high risk of bias. Other conditions were judged to be at moderate risk of bias. We also assessed the certainty of evidence using the Confidence in Network Meta-Analysis framework (CINeMA).

### Data extraction

Two independent researchers followed a preplanned search strategy that required each researcher to perform his research separately according to PRISMA guidelines (29). When multiple papers reported results from the same study at different stages, data was only extracted for the most recent results. Any discrepancies were resolved by consensus. Two investigators used predesigned forms for data extraction and recording. The characteristics of the included patients, including ECOG performance score, age, PSA value, Gleason score, visceral metastasis, tumor burden, proportion of patients with primary metastasis, previous ADT use, and previous/concurrent use of docetaxel, were recorded in detail. In terms of related efficacy results, HR values for OS and PFS were recorded, as well as HR values for different tumor burden subgroups. For safety outcomes, the number of patients with high-grade AEs were recorded, and OR values were calculated.

### Data synthesis strategy

Three similar indicators related to PFS were used in different studies, including PFS (12, 13, 18, 30–32), radiographic progression-free survival (rPFS) (11, 14, 16, 17, 25, 33–36), and clinical progression-free survival (cPFS) (10, 11, 15). Since the three were similar in definition, and in most cases the imaging progression was earlier than the aggravation of clinical symptoms and death, we unified the three into one “generalized PFS” in the present meta-analysis (7). Traditional nonsteroidal antiandrogens (NSAA) have long been considered to result in no or very little improvement in OS in patients with metastatic prostate cancer (27, 28); therefore, we considered NSAA+ADT as the same treatment as ADT alone to pool more data in the present review. In the subgroup analysis, we focused on tumor burden (high- versus low-volume disease), as most RCTs suggested that tumor burden was a feature that influenced the efficacy of interventions. Following the CHAARTED study criteria (11, 36, 37), high volume disease (HVD) was defined as the presence of visceral metastases and/or four or more bone metastases, with at least one bone metastasis located outside the spine and pelvis. We also made subgroup analyses for de novo patients and patients with previous local treatment.

### Statistical analysis

The network meta-analysis was performed using the “gemtc” and “rjags” packages of the R 4·0·5 software using the Monte Carlo Markov Chain (McMc) method in a Bayesian framework (38, 39). A network diagram was drawn for each intervention. The original hazard ratio (HR) values and 95% confidence intervals (CIs) of each RCT study (or their subgroup data) were extracted, and the consistency model was used (40). The number of model chains was 3, the initial value was 2·5, the sampling number was 10,000, and the number of iterations was 100 000 with a step size of 10. For closed-loop studies with both direct evidence and indirect evidence, the node-splitting model was used to test the consistency of closed-loop studies, and P<0·05 was considered statistically significant. The “mtc.anohe” command in the “gemtc” package was used to assess overall heterogeneity and was recorded with the variance parameter I^2^. Heterogeneity was assessed visually using forest plots and I^2^ statistics. If the heterogeneity was large (I^2^>50%, P<0·1), the source of heterogeneity was further analyzed.

The trace plotting method and density plotting method were used to evaluate the convergence of the model. The rank probability was calculated to determine the level of superiority and inferiority of different treatments according to P scores (41). We extracted the “number of patients with grade 3-5 AEs/total patients” in each group and calculated the odds ratio (OR) and 95% CI.

## Results

### Characteristics of eligible studies

**Figure 1:**
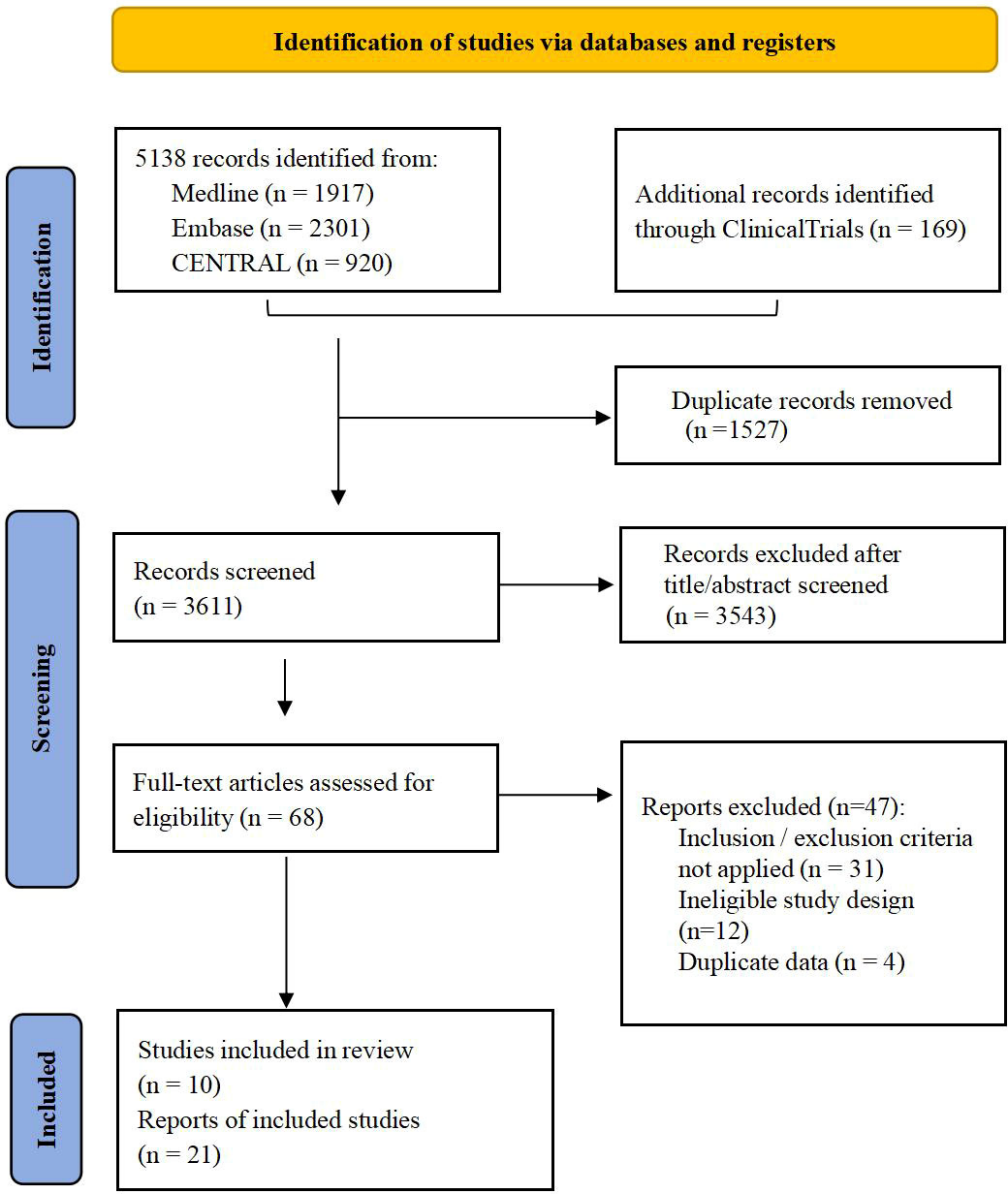
Literature screening flow chart.

A total of 5138 articles and 169 clinical trials were retrieved from the literature search, and 68 of them were read in full text after browsing the titles and abstracts. Finally, ten multicenter phase III randomized controlled trials (21 references) (10–18, 25, 26, 30–37, 42, 43) were included in the analysis (**Figure 1**). A total of 12,298 patients were included, with a median number of patients of 1,069 (range, 385-2061), median follow-up time of 44·6 months (range, 34-84 months), and publication years between 2013 and 2022. The ten trials involved a total of nine treatments, including ADT alone, DOC+ADT, abiraterone and prednisone(AAP)+ADT, ENZA+ADT, APA+ADT, RT+ADT, ENZA+DOC+ADT, AAP+DOC+ADT, and DARO+DOC+ADT. For OS, the overall risk of bias was low in five trials (CHAARTED, STAMPEDE-arm C, TITAN, HORRAD and ARASENS), while the remaining trials raised some concerns. All trials raised some concerns regarding generalized PFS except for the TITAN trial (**Appendix P 5-6**).

The STAMPEDE study (12, 13, 18, 30, 31) allowed the recruitment of localized high-risk patients, and we only extracted data from mHSPC patients in this analysis. In the multiarm, multiphase STAMPEDE study, there was a period of overlap in the recruitment timing of arm C and arm G; thus data from this period could be used to directly compare AAP+ADT and DOC+ADT (32). The PEACE-1 study (25) used a 2 × 2 factorial design for newly diagnosed mHSPC patients. Only the data of the docetaxel population was extracted, and the triplet therapy “AAP+DOC+ADT” and the doublet therapy “DOC+ADT” were compared. The baseline characteristics of all included studies are listed in **Table 1** and **Appendix (P 7-8).**

**Table 1:** Characteristics of included trials.

Trial	Population	Enrollment	Treatment added to ADT	mHSPC cases No. (Exp. *vs*. Ctrl)	Inclusion of interest	Primary endpoint	Secondary endpoint (with interest)	Follow -up, mo
GETUG -AFU15 ^[10,37]^	France and Belgium	Oct. 2004- Dec. 2008	Docetaxel *vs*. no treatment	385 (192 *vs*. 193)	1) no previous chemotherapy; 2) previous hormone therapy in metastatic setting within the past 2 months allowed; 3) 29% with metastases after previous radical treatment; 4) 48% with HVD; 5) daily prednisone use not needed; 6) 66% received ADT combined with NSAA.	OS	cPFS, bPFS, rPFS	84
CHAARTED^[11,38]^	USA	July 2006- Dec. 2012	Docetaxel *vs*. no treatment	790 (397 *vs*. 393)	1) no previous chemotherapy; 2) no previous hormone therapy in metastatic setting; 3) 27% with metastases after previous radical treatment; 4) 65% with HVD; 5) daily prednisone use not needed.	OS	cPFS, time to CRPC	54
STAMPEDE-arm C ^[12] [31]^	UK and Swiss	Oct.2005-Mar.2013	Docetaxel *vs*. no treatment	1086 (362 *vs*. 724)	1) no previous chemotherapy; 2) 5% with metastases after previous radical treatment; 3) 56% with HVD; 4) daily prednisone use needed (5mg, bid); 5) 7% with planned SOC radiotherapy.	OS	FFS, PFS, time to any treatment after progression	78
STAMPEDE-arm G ^[13,30]^		Nov. 2011- Jan. 2014	AAP *vs*. no treatment	1002 (500 *vs*. 502)	1) no prior chemotherapy; 2) no previous long-term hormone therapy; 3) 5% with metastases after previous radical treatment; 4) 55% with HVD; 5) 30% with planned SOC Radiotherapy.	OS	FFS, PFS, symptomatic SRE, adverse events, QOL, PCa-specific survival	42
STAMPEDE-arm H ^[18]^		Jan. 2013- Sep.2016	Radiotherapy *vs*. no treatment	2061 (1032 *vs*. 1029)	1) 18% with planned DOC chemotherapy (≤6 cycles and without disease progression); 2) 100% newly diagnosed mPCa; 3) 58% with HVD.	OS, FFS	PFS, mPFS, prostate cancer-specific survival, and symptomatic local event-free survival	37
LATITUDE ^[14,36]^	Worldwide (34 countries)	Feb. 2013- Dec.2014	AAP *vs*. placebo	1199 (597 *vs*. 602)	1) no prior chemotherapy, radiation therapy, or surgery; 2) previous hormone therapy in metastatic setting within the past 3 months allowed; 3) 100% newly diagnosed mPCa; 4) mHSPC with two of three high risk factors, 80% with HVD.	OS, rPFS	Time to PSA progression, Time to next symptomatic SRE, Time to subsequent PCa therapy	52
ENZAMET ^[15]^	6 countries, maily Australian and Canada	Mar.2014-Mar.2017	Enzalutamide *vs*. NSAA	1125 (563 *vs*. 562)	1) 16% with DOC chemotherapy (≤2 cycles and without disease progression) before randomization; 2) previous hormone therapy in metastatic setting within the past 3 months allowed; 3) 39% with metastases after previous radical treatment; 4) 52% with HVD; 5) 44.7% with planned early DOC used.	OS	cPFS, adverse events	34
ARCHES ^[16,33,35]^	Worldwide (24 countries)	Mar. 2016- Jan.2018	Enzalutamide *vs*. placebo	1150 (574 *vs*. 576)	1) 18% with previous chemotherapy (≤6 cycles and without disease progression); 2) no previous hormone therapy in metastatic setting; 3) 26% with metastases after previous radical treatment; 4) 63% with HVD; 5) 435(38%) had received prior AA. 5) 6.7% received concomitant antiandrogens.	OS, rPFS	time to PSA progression, time to initiation of new antineoplastic therapy, objective response	44.6
TITAN ^[17,34]^	Worldwide (23 countries)	Dec.2015- July 2017	Apalutamide *vs*. placebo	1052 (525 *vs*. 527)	1) 10.7% with previous chemotherapy (≤6 cycles and without disease progression); 2) previous hormone therapy in metastatic setting within the past 3 months allowed; 3) 16% with metastases after previous radical treatment; 4) 63% with HVD; 5) NSAA allowed before randomization.	OS, rPFS	Time to chemotherapy, time to pain progression, time to chronic opioid use, time to SRE.	44
HORRAD [^43]^	Netherlands	Nov. 2004- Sep.2014	Radiotherapy *vs*. no treatment	432 (216 *vs*. 216)	1) no previous chemotherapy; 2) 100% newly diagnosed mPCa; 3) 83% with high burden in HORRAD definition.	OS	PSA-PFS	47
PEACE-1 (Docetaxel population) ^[25]^	7 European countries	Nov. 2013- Dec. 2018	AAP+DOC *vs*. DOC	710 (355 *vs*. 355)	1) 100% *de novo* mHSPC; 2) no previous long-term hormone therapy; 3) 64% with HVD; 4) Concomitant DOC and abiraterone use; 5) Full 6 cycles of DOC administered in 100% of patients; 6) ± radiotherapy allowed.	rPFS; OS	CRPC-free survival, cPFS, Prostate cancer specific survival, Toxicity	42
ARASENS ^[26]^	Worldwide (23 countries)	Nov. 2016- June 2018	DARO+DOC *vs*. DOC	1306 (651 *vs*. 654)	1)100% *de novo* mHSPC. 2) No previous chemotherapy, 2nd AR inhibitor, immunotherapy, or radiotherapy within 2 weeks before randomization; 3) NSAA allowed but should be discontinued before randomization.	OS	CRPC-free survival, time to pain progression, time to initiation of new antineoplastic therapy, safety	43.7 (DARO) 42.4 (Ctrl)

AA, antiandrogen drugs; AAP, abiraterone acetate+prednisone; ADT, androgen deprivation therapy; AR, androgen receptor; bPFS, biochemical progression free survival; cPFS, clinical progression free survival; CRPC, castration resistant prostate cancer; Ctrl, control group; DARO, darolutamide; DOC, docetaxel; Exp., experimental group; FFS, failure free survival; HVD, high volume disease in CHAARTED definition; mHSPC, metastatic hormonal-sensitive prostate cancer; mPCa, metastatic prostate cancer; mPFS, metastatic progression free survival; NSAA, non-steroidal anti-androgen; including nilutamide, flutamide or bicalutamide; OS, overall survival; QOL, quality of life; rPFS, radiographic progression free survival; SRE, skeletal related events; SOC, standard of care.

There was a certain proportion of patients with “early planned docetaxel used” in ENZAMET (15), ARCHES (16, 42), TITAN (17, 33) and STAMPEDE arm H (18) trials. There were obvious differences in HR values for OS between the docetaxel population and the population that did not use docetaxel (**Table 2**), which suggested that the treatment sequence of docetaxel followed by NHAs may limit the effectiveness of NHAs (15, 16, 18, 33, 42). Thus, in our review, we strictly evaluated the impact of “planned docetaxel use” on the efficacies of NHAs or radiotherapy in relevant studies (15, 16, 18, 33, 42). We strictly excluded data from the docetaxel population in effectiveness assessment, unless data extraction was difficult in some subgroups. In the ENZAMET and ARCHES studies, 44·7% and 17·8% of patients received “early planned docetaxel chemotherapy”, respectively, and these patients actually received triplet therapy. We extracted data from these docetaxel populations and made an exploratory analysis on the comparative efficacy of triplet therapy of “ENZA+DOC+ADT” and doublet therapy of “DOC+ADT”. For similar data of the docetaxel population in the TITAN and STAMPEDE arm H studies, we did not conduct a similar analysis due to the small number of patients and incomplete data (**Appendix P 9**).

**Table 2:** Clinical trials or subgroups related to mHSPC’s triplet therapies.

Study	Available comparison	Patients in available subgroups (n, %)	*de novo* mHSPC	Start of DOC use to NHA or RT	Proportion of pts with full 6 cycles of DOC administered	OS for DOC population (HR, 95% CI)	OS for non-DOC population (HR, 95% CI)	Generalized PFS forDoc population (HR,95% CI)
ENZAMET ^[15]^	ENZA+DOC+ADT *vs*. DOC+ADT	503(44.7%)	61%	Prior (35%) and Concomitant (65%)	71% (65% in ENZA group and 76% in control group)	0.90 (0.62-1.31)	0.53, 0.37-0.75	cPFS: 0.48 (0.37-0.62)
ARCHES ^[16,33,35]^	ENZA+DOC+ADT *vs*. DOC+ADT	205(17.8%)	67%	Prior	86%	0.74 (0.46-1.20)	0.64, 0.51-0.81	rPFS 0.52 (0.30-0.89)
TITAN^[17,34]^	APA+DOC+ADT *vs*. DOC+ADT	113(10.7%)	81%	Prior	NR (In median, 6 cycles administered)	1.12 (0.59-2.12)	0.61, 0.50-0.76	NR
STAMPEDE -RT ^[18]^	RT+DOC+ADT *vs*. DOC+ADT	367(17.8%)	100%	Prior	NR	0.81 (0.49-1.34)	0.93, 0.80-1.08	NR
PEACE-1^[25]^	AAP+DOC+ADT *vs*. DOC+ADT	710(60.5%)	100%	Concomitant	100%	0.75 (0.59-0.95)	NA	rPFS: 0.50 (0.40-0.62)
ARASENS^[26]^	DARO+DOC+ADT *vs*. DOC+ADT	1306(100%)	86%	Concomitant	86.6%	0.68 (0.57-0.80)	NA	NR

AAP: abiraterone and prednisone; ADT: androgen deprivation therapy; APA: apalutamide; CI: confidence interval; cPFS: clinical progression free survival; DARO: darolutamide; DOC: docetaxel; ENZA: enzalutamide; HR: hazard ratio; NA: not applicable; NHA: novel hormonal agent; NR: not reported; PFS: progression free survival; rPFS: radiographic progression free survival; RT: radiotherapy.

### OS for the overall population

**Figure 2:**
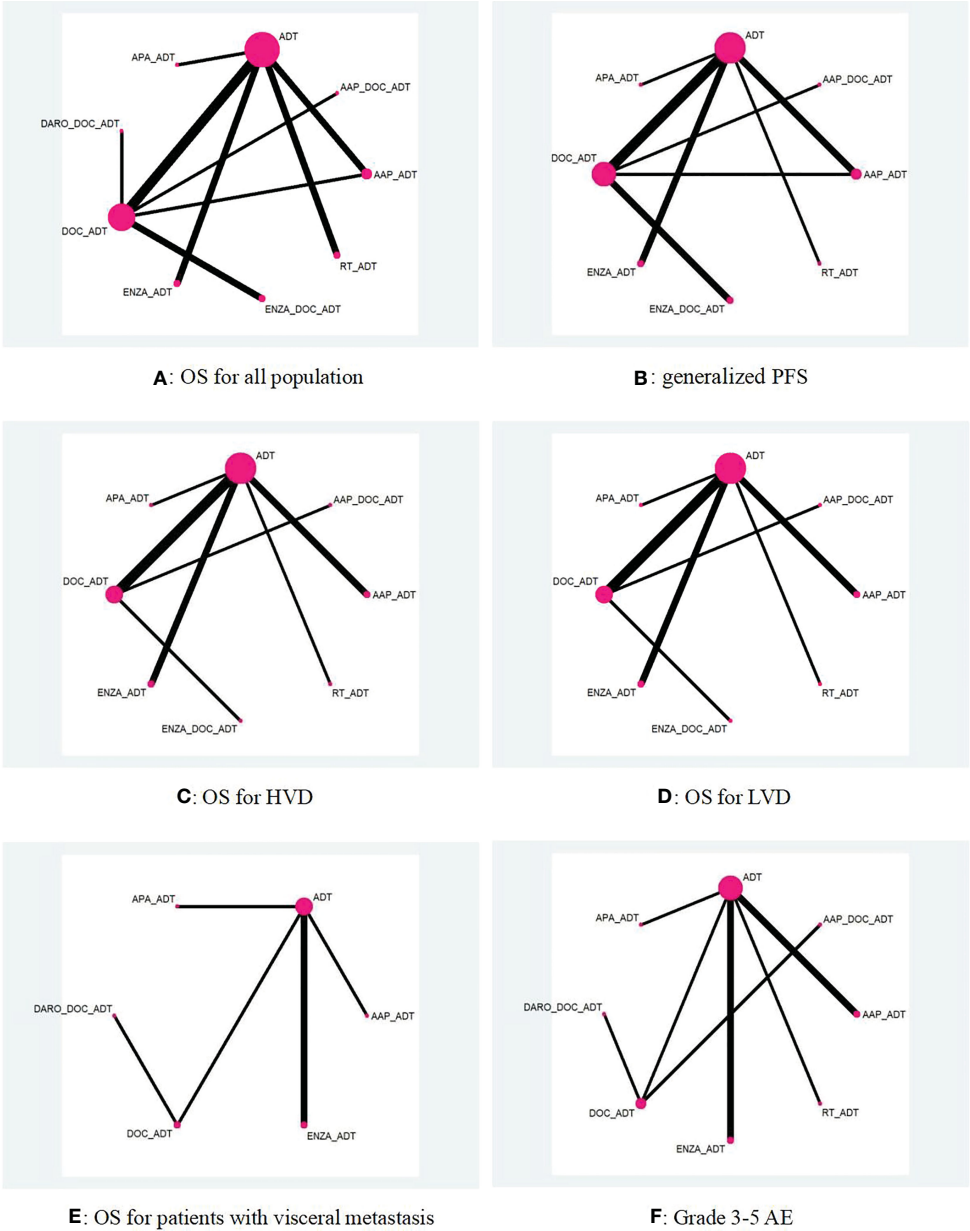
Network diagram of different treatment options for mHSPC **(A–F)**. AAP, abiraterone acetate and prednisone; ADT, androgen deprivation treatment; APA, apalutamide; DARO, darolutamide; DOC, docetaxel; ENZA, enzalutamide; HVD, high volume disease; LVD, low volume disease; OS, overall survival; PFS, progression free survival; RT, radiotherapy; The connector line “_” represents combination of different treatment regimens.

**Figure 2** shows a network diagram comparing different treatment options. Only data from 342 mHSPC patients from arms C and G of the STAMPEDE study, a multiarm, multistage RCT, were extracted for a head-to-head comparison (32). Consistency tests for this closed-loop comparison suggested that p values were all >0.1 (**Appendix P 11**). The results of the heterogeneity test indicated that I^2 =^ 60.1% for the comparison of abiraterone and docetaxel, and the heterogeneity was derived from the direct comparison between the two agents (**Appendix P 12-18**).

**Figure 3:**
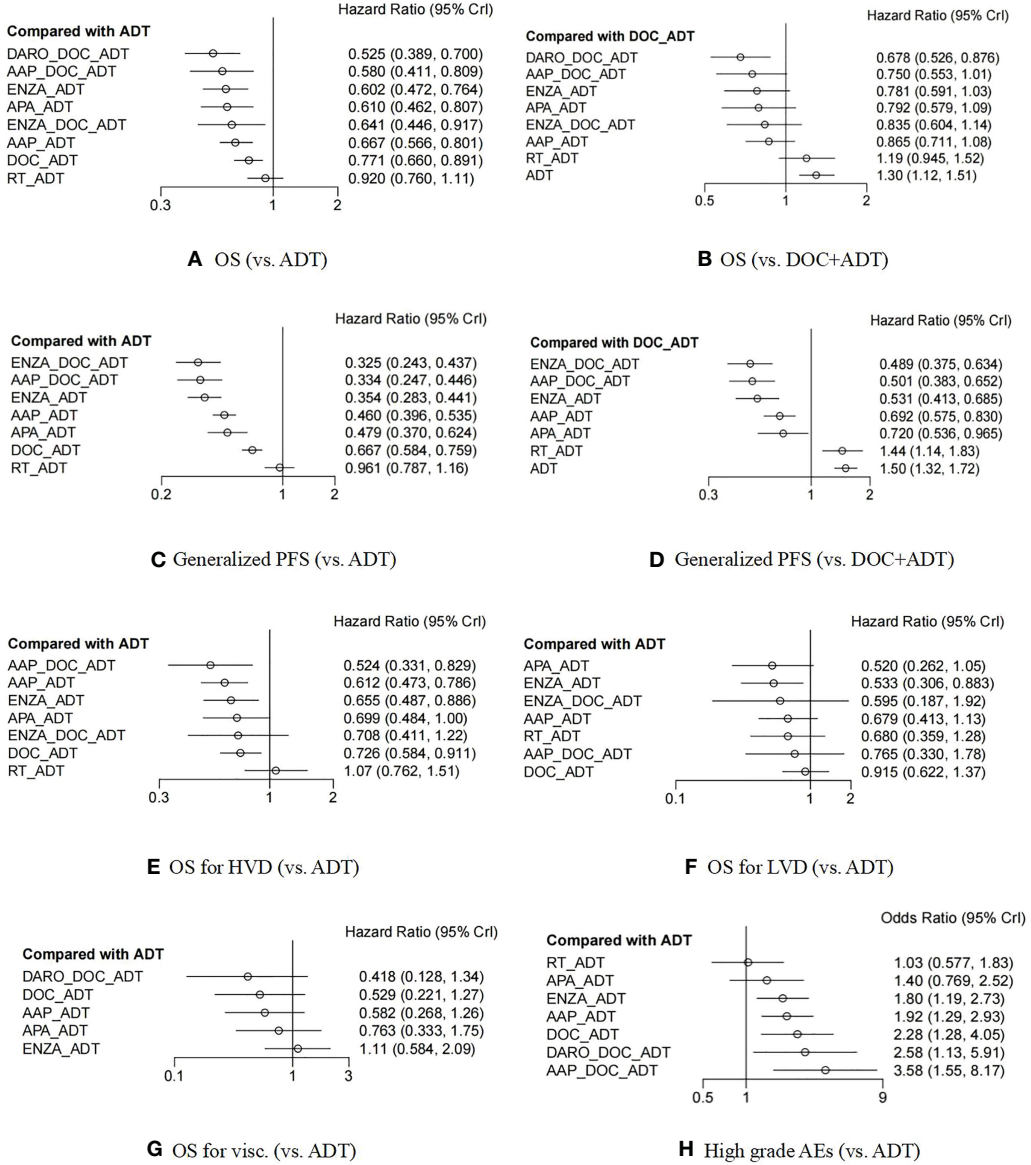
Forest plot between different treatment options and ADT monotherapy (or DOC+ADT) in patients with metastatic hormonal-sensitive prostate cancer **(A–H)**. ADT, androgen deprivation treatment; DOC, docetaxel; AAP, abiraterone acetate and prednisone; ENZA, enzalutamide; APA, apalutamide; RT, radiotherapy; DARO, darolutamide; OS, overall survival; PFS, progression free survival; HVD, high volume disease; LVD, low volume disease; Visc., patients with visceral metastasis; AEs, adverse events. The connector line “_” represents combination of different treatment regimens.

The STAMPEDE study had four comparison groups, arm C (12), arm G (13), and arm H (18), and a head-to-head subgroup (32). While in both ENZAMET (15) and ARCHES (16, 42) studies, independent analyses of the “docetaxel population” were performed. Therefore, a total of 15 comparison groups were obtained from the ten trials (**Appendix P 9**), and the data of these 15 groups were all included in the network meta-analysis of OS. The forest plot demonstrated a comparison with ADT alone or DOC+ADT and is shown in **Figures 3A, B**.

Except for radiotherapy (HR 0·92, 95% CI 0·76-1·11), the other seven treatment options were all significantly better than ADT in terms of OS benefits. Compared with docetaxel chemotherapy, only the triplet therapies of DARO+DOC+ADT (HR 0·68, 95% CI 0·53-0·88) had significant OS benefits. Additionally, the triplet therapy of ENZA+DOC+ADT was not superior to ENZA+ADT (HR 1·07, 95% CI 0·70-1·64). The detailed HR data of the comparison between each two treatment groups can be found in **Appendix (P 21-24).** The ranking and ranking probability of competing interventions are shown in **Appendix (P 25-32).** The triplet treatment DARO+DOC+ADT had the highest probability of ranking the best. The convergence of the models was well according to trace and density plots (**Appendix P 33-34**).

### Generalized PFS for the overall population

In addition to the HORRAD (43) and ARASENS (26) trials, 13 of the 15 comparison groups reported results of PFS or similar indicators (**Appendix P 9**). Specifically, six comparison groups (GETUG-AFU15 (10, 36), LATITUDE (14, 35), ARCHES (16, 42) and its docetaxel subgroup, TITAN (17, 33), and PEACE-1 (25) reported results of rPFS, four comparison groups (STAMPEDE arm C (12), arm G (13), arm H (18), and AAP-DOC head-to-head comparison subgroup (32) reported results of PFS, and cPFS was reported in four comparison groups (GETUG-AFU15 (10), CHAARTED (11), ENZAMET (15), and ENZAMET’s docetaxel subgroup). We unified these indicators as a “generalized PFS” (We chose rPFS, rather than cPFS, in GETUG-AFU15 for subsequent comparison).

The results showed that except for radiotherapy (HR 0·96, 95% CI 0·80-1.16), all other measures were significantly better than ADT (**Figures 3C, D**). In addition, except for ADT (HR 1·50, 95% CI 1·32-1·72) and radiotherapy (HR 1·44, 95% CI 1·14-1·83), which were inferior to docetaxel chemotherapy, other treatments were all superior to docetaxel chemotherapy. The ranking probability and HR values for the pairwise comparisons of each treatment measure can be found in **Appendix (P 21-32).** The triplet therapy ENZA+DOC+ADT had the highest probability of ranking the best. Besides, we also made respective analyses for rPFS, PFS and cPFS (The forest plots can be seen in **Appendix P 20**).

### OS for the population in subgroups

OS data of high- or low- volume subgroups were extracted from 11 of the 15 comparison groups and 8 treatments were included (**Appendix P 9**). The analysis results of mHSPC with high volume disease (HVD) showed that radiotherapy (HR 1·07, 95% CI 0·76-1·51) had no benefit compared with ADT, the benefit of ENZA+DOC+ADT (HR 0·71, 95% CI 0·41-1·22) had no significant difference compared with ADT, while the other five treatments all had significant benefits compared to ADT (**Figure 3E**). In terms of possible ranking, the best was AAP+DOC+ADT (HR 0·52, 95% CI 0·33-0·83), followed by AAP+ADT (HR 0·61, 95% CI 0·47-0·79). The results of low tumor disease (LVD) showed that APA+ADT (HR 0·52, 95% CI 0·26-1·05) and ENZA+ADT (HR 0·53, 95% CI 0·31-0·88) possibly ranked the highest (**Figure 3F**). However, it has to be emphasized that ARASENS trial did not stratify the patients according to disease volume.

Only six studies reported HR data in patients with visceral metastases, involving five interventions (**Appendix P 9).** The results suggested that DARO+DOC+ADT (HR 0·42, 95% CI 0·13-1·34) had the highest probability of ranking the best, while the new antiandrogens, especially enzalutamide (HR 1·11, 95% CI 0·58-2·09), performed less well. (**Figures 3G** and **Appendix P 25-32**)

### Safety

In terms of safety indicators, we selected grade 3-5 AEs, as there were more studies reporting grade 3-5 AEs than serious adverse events (SAEs). In particular, two important trials related to triplet therapies, PEACE-1 (25) and ARASENS (26), both reported grade 3-5 AEs. The CHAARTED study (11) reported grade 3-5 AEs in the docetaxel group, but corresponding data in the control group were lacking; thus, these data were not used. No data from GETUG -AFU15 (10, 36) were used, as they reported a different safety indicator of “AE events/person month”. Finally, we included a meta-analysis of grade 3-5 AEs from nine studies (**Appendix P 45**) involving eight different interventions.

The results showed that the risk of grade 3-5 AEs in triplet therapies was relatively high, especially that of AAP+DOC+ADT (**Figure 3H**). The order of security was: ADT alone (comparator) > RT+ADT (1·03, 0·57-1·87) > APA+ADT (1·39, 0·76-2·55) > ENZA+ADT (1·81, 1·18-2·77) > AAP+ADT (1·92, 1·27-2·95) > DOC+ADT (2·28, 1·26-4·16) > DARO+DOC+ADT (2·59, 1·11-6·09) > AAP+DOC+ADT (3·56, 1·51-8·43).

The certainty of the evidence for outcomes of interest as measured with CINeMA varied from high to very low. Full information on CINeMA is described in the appendix (**Appendix P 46–64**).

## Discussion

We performed a comprehensive network meta-analysis on the first-line therapies for mHSPC recommended by major guidelines such as EAU (2) and NCCN (44). Compared with previous similar meta-analyses, our paper not only included the final OS data of the ARCHES (42) and TITAN (33) trials but also conducted an in-depth analysis of three different triplet therapies: the darolutamide triplet therapy and the abiraterone triplet therapy came from newly reported outcomes of ARASENS (26) and PEACE-1 (25) trials, while data of the third therapy, the enzalutamide triplet therapy, were extracted from the docetaxel population from previous ENZAMET (15) and ARCHES (16, 34, 42) studies.

This paper has two features in data extraction. First, in the OS analysis, the docetaxel population in multiple studies (including ENZAMET (15), ARCHES (16, 34, 42), TITAN (17, 33), and STAMPEDE arm-H (18) was excluded, as we found that the HRs for OS of interventions in the docetaxel population in the above trials were significantly different from those of the nondocetaxel population. The use of docetaxel was likely to affect the accurate evaluation of the efficacy of interventions. Second, we reintegrated and utilized the docetaxel population data in the ENZAMET (15) and ARCHES (16, 34, 42) trials to evaluate whether enzalutamide triple therapy was superior to docetaxel treatment.

Our results showed that triplet therapies with darolutamide or abiraterone were likely to further improve OS benefits. However, ENZA+DOC+ADT did not show an OS benefit compared with ENZA+ADT or DOC+ADT, and the possible explanations might be as follows: 1) In the ENZAMET and ARCHES studies, whether patients chose to accept “planned early docetaxel use” was not random but mainly dependent on the judgment of the attending doctors, while the ENZAMET study was also not blinded; 2) In the docetaxel population of the ENZAMET study, there was a certain difference between the enzalutamide group and the control group in the proportion of patients receiving six complete courses of chemotherapy, which were 65% and 76%, respectively; 3) A considerable proportion of patients received docetaxel before enzalutamide (35% in ENZAMET, 100% in ARCHES), rather than receiving novel hormonal agents and docetaxel chemotherapy concurrently as in the PEACE-1 and ARASENS studies. However, in the comparison of “generalized FPS” benefits, enzalutamide triplet therapy was “unexpectedly” ranked first. Since the orders of pros and cons of several other interventions in terms of PFS were almost exactly the same as those in terms of OS, we highly suspected that the poor performance of triplet therapy with enzalutamide in terms of OS was more likely to be influenced by external factors such as patient baseline and medication use.

For different tumor burden or visceral metastasis subgroups, the rankings of interventions fluctuate greatly. For example, ENZA+ADT ranked almost highest for LVD patients but lowest for patients with visceral metastasis. This suggests that we should consider a variety of factors when choosing treatment for mHSPC patients, especially the burden and location of metastasis.

In the PEACE-1 trial, AAP+DOC+ADT had a significant advantage over DOC+ADT in overall OS, and in STAMPEDE arm H, RT+ADT had a significant advantage over ADT in OS for low volume disease. However, treatment superiority in these randomized controlled trials became insignificant in our meta-analysis. Considering the power of indirect comparison of network metastasis, when this happens, the results of RCTs are more reliable. However, we also look forward to direct comparisons from more RCTs to further clarify the superiority of the above treatment combinations.

There are some deficiencies in this meta-analysis: 1) We considered “ADT+NSAA” therapy (27, 28) in the control group of the ENZAMET trial the same and analyzed it consolidated with “ADT+placebo” or “ADT+ no treatment” in other trials to make the interpretation of the results more concise and to make it possible to combine docetaxel populations in the ENZAMET and ARCHES trials. However, it will be questioned in terms of rigor. 2) Strictly speaking, PFS, rPFS and cPFS are not the same indicators. Once they are combined into “generalized PFS” for analysis, caution should be taken in the interpretation of the results. 3) For the studies of ENZAMET, ARCHES, TITAN and STAMPEDE arm H, we excluded the data of the docetaxel population in the comparison of HRs (OS) to ensure that HR data were not affected by docetaxel chemotherapy. However, in the analysis of some HRs for OS and PFS of high/low volume disease subgroups, due to the difficulty of data acquisition, we did not exclude the data of the docetaxel population. 4) In triplet therapies, many drugs were given sequentially rather than concurrently (**Table 2**), these differences could reduce the reliability of our network meta-analysis. 5) According to CINeMA, we rated many comparisons as low or very low quality, especially for OS in subgroups analysis, which restricts the interpretation of our results. The reasons for downgrading mainly come from items of imprecision and incoherence, and the low quality may be related to small sample size of included trials and too many indirect comparisons.

## Conclusion

Our results showed that compared with ADT alone, ADT combined with docetaxel, abiraterone acetate, enzalutamide or apalutamide had significant benefits for OS, and the newly emerging triplet therapies may further increase the benefits of OS but at the expense of a certain decrease in safety. Our results suggested that the standard treatment of mHSPC could be considered transferring to the triple therapy of NHAs+DOC+ADT after the transition from ADT alone to NHA/DOC+ADT, especially for patients with high volume disease. However, in most cases, the differences in efficacy between the different interventions are not significant, and they show different efficacy profiles when considering different subgroups, which allows clinicians considerable flexibility in choosing treatment options for individual patients.

## Data availability statement

The original contributions presented in the study are included in the article/**Supplementary Material**. Further inquiries can be directed to the corresponding authors.

## Author contributions

Conceptualization: LW and FS. Data curation: LW, CL, ZZ, and XL. Formal statistical analysis: LW, CL, and FS. Methodology: LW and CL. Software: CT. Validation: ZG and FS. Writing-original draft: LW and CL. Writing-review and editing: JG and NL. All authors had full access to all of the data, and the corresponding authors had the final responsibility to submit for publication. All authors contributed to the article and approved the submitted version.
