# Supplementary material for: Comparison of doublet and triplet therapies for metastatic hormone-sensitive prostate cancer: A systematic review and network meta-analysis
Source: Front Oncol. 2023 Mar 7;13:1104242. doi: 10.3389/fonc.2023.1104242 (PMC10028133; doi:10.3389/fonc.2023.1104242)
Supplement: Supplementary file 1 [file DataSheet_1.pdf]

Supplementary appendix for

Comparison of Doublet and Triplet Therapies for Metastatic Hormone-Sensitive Prostate Cancer: A Systematic Review and Network Meta-analysis

Pages 1-4: Search strategy
Pages 5-6: Risk of study bias
Pages 7-8: Patients' supplementary information of included trials
Page 9-10: Hazard Ratio for OS and PFS in included trials or subgroups
Page 11: Consistency test of closed-loop studies
Pages 12-18: Heterogeneity test
Pages 19-20: Supplementary forest plots
Pages 21-24: Relative effect estimates for all possible treatment comparisons
Pages 25-32: OS/PFS ranking and ranking probability of competing interventions based on overall population
Pages 33-44: Trace and density plots
Page 45: Summary of grade 3-5 adverse events in reported trials
Pages 46-64: Assessment of the certainty of evidence using the confidence in network meta-analysis (CINeMA)

Supplementary Material 1: Search strategy

MEDLINE (PubMed interface)

Related to mHSPC

#1 Prostatic Neoplasms[Mesh] OR (prostat*[tw] AND cancer[tw])

#2 prostat*[tiab] AND (canc*[tiab] OR tumor[tiab] OR carcinom*[tiab] OR adeno*[tiab] OR malignan*[tiab] OR tumour*[tiab] OR neoplas*[tiab])

#3 #1 OR #2

#4 metastasis[Mesh] OR metasta*[tw] OR advanced[tw]

#5 Hormones[Mesh] OR hormon*[tw] OR castrat*[tw] OR androgen*[tw] OR testosterone[tw]

#6 sensitive[tw] OR naive[tw] OR primary[tw] OR de novo[tw] OR newly diagnosed[tw]

#7 #3 AND #4 AND #5 AND #6

Related to intervention

#8 Docetaxel[tw] OR Docetaxol[tw] OR RP 56976[tw] OR RP56976[tw] OR Taxotere[tw] OR NSC 628503[tw] OR Daxotel[tw]

#9 abiraterone[tw] OR CB-7598[tw] OR CB7598[tw]

#10 MDV3100[tw] OR Enzalutamide[tw] OR MDV-3100[tw] OR xtandi[tw]

#11 apalutamide[tw] OR ARN-509[tw] OR arn509[tw] OR erleada[tw]

#12 darolutamide[tw] OR odm201[tw] OR ODM-201[tw]

#13 Radiotherapy[Mesh] OR radiotherap*[tw] OR radiation [tw] OR irradiation [tw]

#14: #8 OR #9 OR #10 OR #11 OR #12 OR #13

Related to RCT

#15 randomized controlled trial [pt]

#16 controlled clinical trial [pt]

#17 randomized [tiab]

#18 placebo [tiab]

#19 Drug therapy [sh]

#20 Randomly [tiab]

#21Trial [tiab]

#22Groups [tiab]

#23: #15 OR #16 OR #17 OR #18 OR #19 OR #20 OR #21 OR #22

#24 Animals [mh] NOT humans [mh]

#25: #23 NOT #24

Combined items

#26: #7 AND #14 AND #25

EMBASE

Related to prostate cancer

1. ‘prostate carcinoma’/exp

2. 'prostate cancer'/exp

3. 'prostate tumor'/exp
4. (prostat* NEAR/3 adeno*):ti,ab,kw
5. (prostat* NEAR/3 malignan*):ti,ab,kw
6. (prostat* NEAR/3 canc*):ti,ab,kw
7. (prostat* NEAR/3 carcinoma*):ti,ab,kw
8. (prostat* NEAR/3 tumo?r*):ti,ab,kw
9. (prostat* NEAR/3 neoplas*):ti,ab,kw
10. 1 OR 2 OR 3 OR 4 OR 5 OR 6 OR 7 OR 8 OR 9

Related to metastasis
11. metasta*/exp 1035036
12. (metast* NEAR/3 prostat*):ti,ab,kw
13. (advanc* NEAR/3 prostat*):ti,ab,kw
14. 11 OR 12 OR 13

Related to hormone-sensitive
15. (hormon* NEAR/3 prostat*):ti,ab,kw
16. (castrat* NEAR/3 prostat*):ti,ab,kw
17. (androgen NEAR/3 prostat*):ti,ab,kw
18. 15 OR 16 OR 17
19. sensitive/exp
20. naive/exp
21. 19 OR 20
22. 18 AND 21
23. (primary NEAR/6 prostat*):ti,ab,kw
24. ('de novo*' NEAR/6 prostat*):ti,ab,kw
25. (new* diagnos*):ti,ab,kw
26. 22 OR 23 OR 24 OR 25

Related to intervention
27. 'docetaxel'/exp OR (Docetaxel OR Docetaxol OR "RP 56976" OR RP56976 OR Taxotere OR "NSC 628503" OR nsc628503 OR Daxotel OR dexotel):ti,ab,kw
28. 'abiraterone'/exp OR (abiraterone OR 'CB-7598' OR CB7598):ti,ab,kw
29. 'enzalutamide'/exp OR (Enzalutamide OR 'MDV-3100' OR MDV3100 OR xtandi):ti,ab,kw
30. 'apalutamide'/exp OR (apalutamide OR 'ARN-509' OR arn509 OR erleada):ti,ab,kw
31. 'darolutamide'/exp OR (darolutamide OR 'ORM-16497' OR 'ORM-16555' OR 'ODM-201' OR odm201):ti,ab,kw
32. 'radiotherapy'/exp OR (radiotherapy OR 'radiotherap*' OR 'radiation' OR 'irradiation'):ti,ab,kw
33. 27 OR 28 OR 29 OR 30 OR 31 OR 32

Related to randomised controlled trials
34. 'randomi*ed controlled trial'/exp
35. 'controlled clinical trial'/exp
36. randomi*ed:ti,ab,kw

37. placebo:ti,ab,kw
38. 'clinical trial':ti,ab,kw
39. randomly:ti,ab,kw
40. 'double blind':ti,ab,kw
41. trial:ti,ab
42. groups:ti,ab
43. 34 OR 35 OR 36 OR 37 OR 38 OR 39 OR 40 OR 41 OR 42
44. ('animal'/exp OR 'animal experiment'/exp) NOT 'human'/exp
45. 43 NOT 44

Combining all terms
46. 10 AND 14 AND 26 AND 33 AND 45

Cochrane CENTRAL

Terms specific to prostate cancer:
1. MeSH descriptor: [Prostatic Neoplasms] explode all trees
2. (prostat* near/3 malignan*):ti,ab,kw
3. (prostat* near/3 canc*):ti,ab,kw
4. (prostat* near/3 carcinoma*):ti,ab,kw
5. (prostat* near/3 tumor*):ti,ab,kw
6. (prostat* near/3 neoplas*):ti,ab,kw
7. #1 or #2 or #3 or #4 or #5 or #6

Terms specific to metastatic disease:
8. MeSH descriptor: [Neoplasm Metastasis] explode all trees
9. (metast* near/3 prostat*):ti,ab,kw
10. (advanced* near/3 prostat*):ti,ab,kw
11. #8 or #9 or #10

Terms specific to hormonal sensitive:
12. hormon*:ti,ab,kw
13. castra*:ti,ab,kw
14. #12 OR #13
15. sensitive:ti,ab,kw
16. naive:ti,ab,kw
17. #15 OR #16
18. #14 AND #17
19. de-novo:ti,ab,kw
20. (new* near/3 diagnos*):ti,ab,kw
21. #19 OR #20
22. #18 OR #21

COMBINED

23. #7 and #11 and #22

ClinicalTrials.gov

Condition or disease: Prostate Cancer Metastatic
Study Type: Interventional Studies (Clinical Trials)
Study Results: Studies With Results
Last update posted：from 01/06/2017 to 01/06/2022

Supplementary Material 2: Risk of study bias for OS (A) or PFS (B).

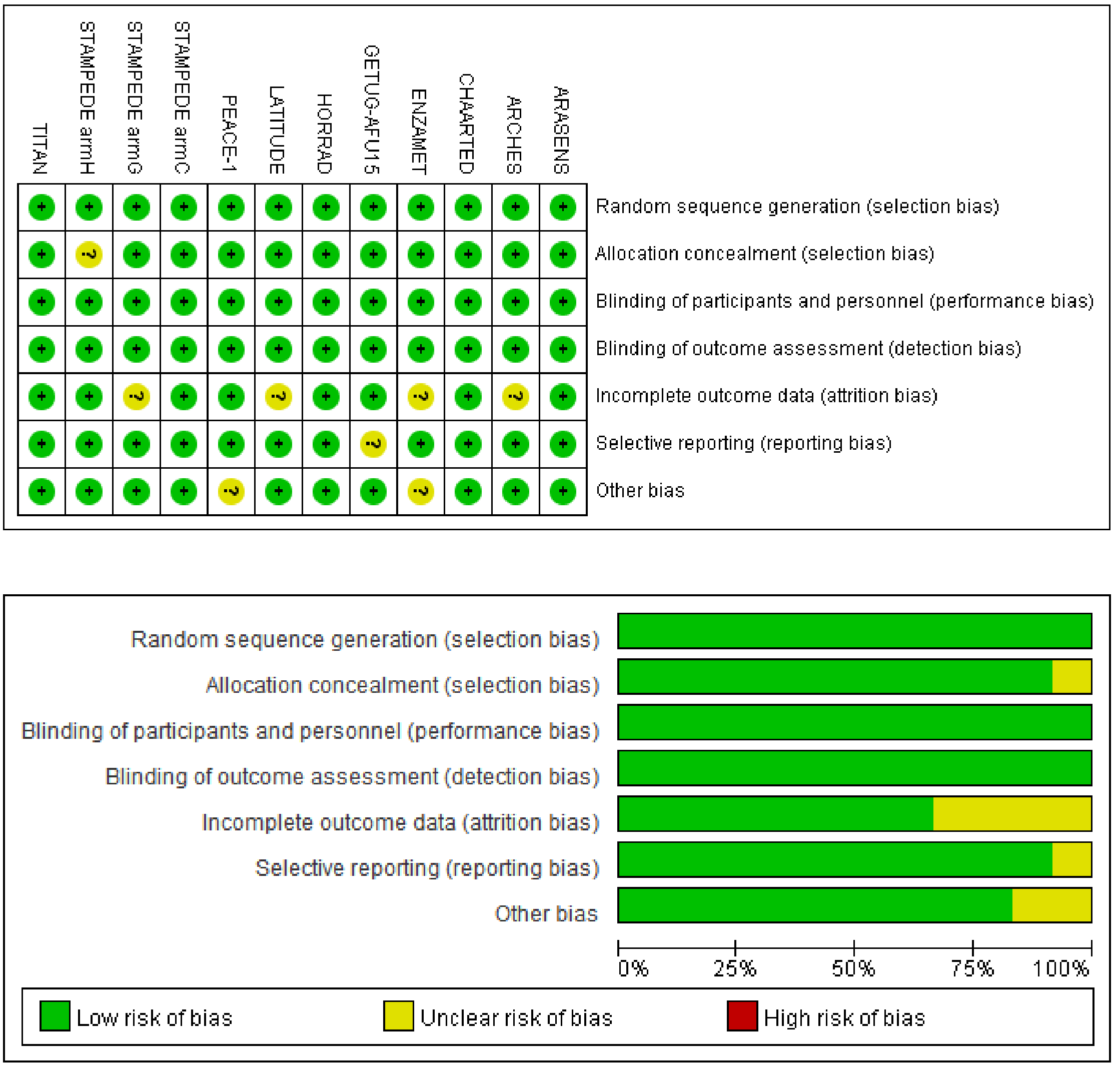

A

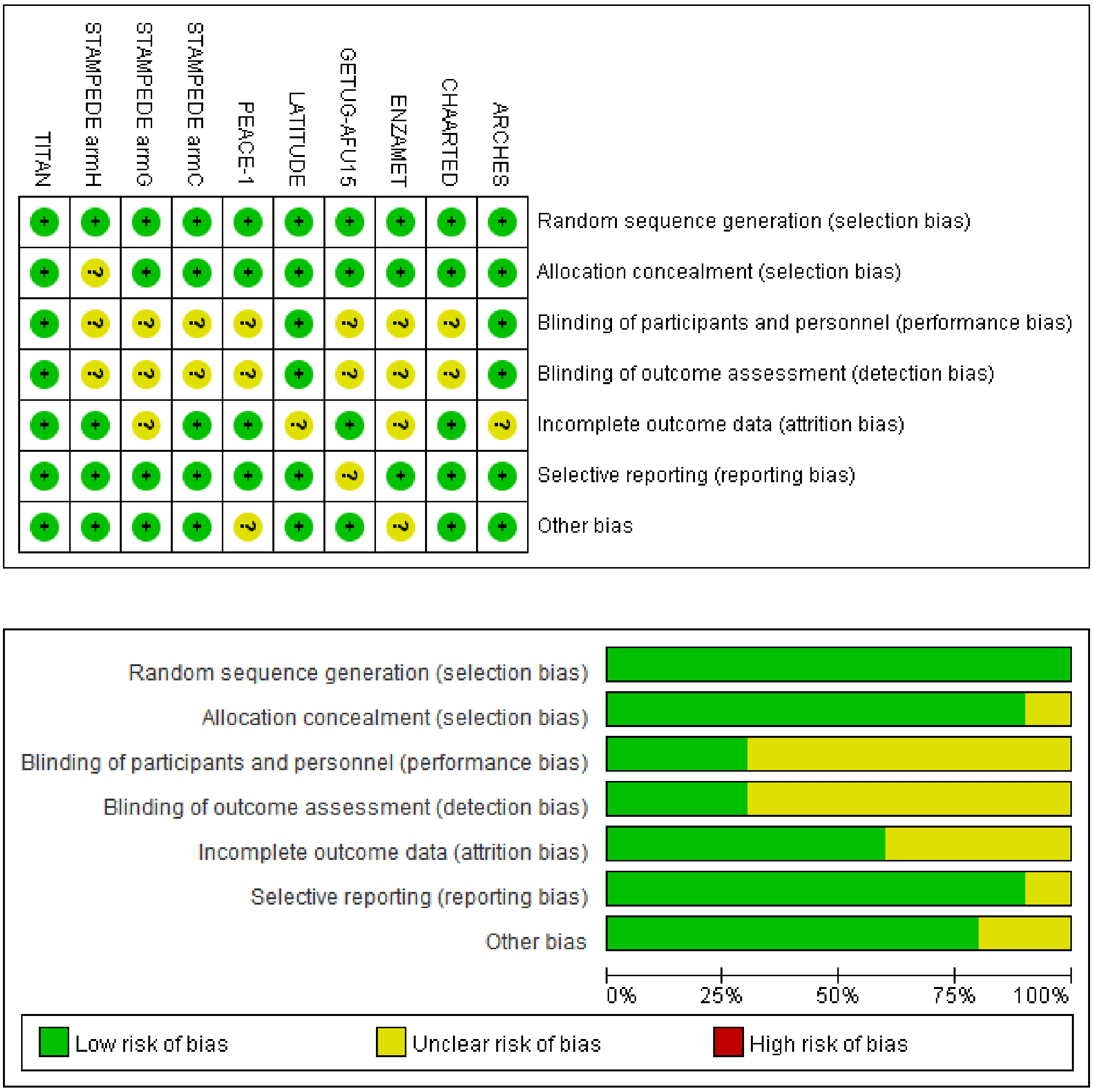

TITAN
STAMPEDE armH
STAMPEDE armG
STAMPEDE armC
PEACE-1
LATITUDE
GETUG-AFU15
ENZAMET
CHAARTED
ARCHES
Random sequence generation (selection bias)
Allocation concealment (selection bias)
Blinding of participants and personnel (performance bias)
Blinding of outcome assessment (detection bias)
Incomplete outcome data (attrition bias)
Selective reporting (reporting bias)
Other bias
0%
25%
50%
75%
100%
Low risk of bias
Unclear risk of bias
High risk of bias

B

Supplementary Material 3: Patients' supplementary information of included trials.

Trial	Performance status (ECOG/WHO grade, %)	Age (Exp. vs. Ctrl, yrs)	PSA (Exp. vs. Ctrl, ng/ml)	Gleason score (Exp. vs. Ctrl, no. %)	Visceral metastases (Exp. vs. Ctrl, no. %).	Metastatic burden (CHAARTED definition, Exp. vs. Ctrl, no. %)	Metastases at initial diagnosis (Exp. vs. Ctrl, no. %)	Patients with planned/previous docetaxel use (Exp. vs. Ctrl, no. %)
GETUG -AFU15[9, 35]	Grade 0, 97.5%<br>Grade 1-2, 2.5%	Median(IQR): 63 (57-68) vs. 64 (58-70)<br>Range: NR	Median(IQR): 26.7 (5-106) vs. 25.8 (5-126)<br>Range: NR	≤7: 84(45) vs. 78(41)<br>8-10: 103(55) vs. 113(59)<br>Unknown: 2 vs. 5	28 (15) vs. 23 (12)	LVD: 100(52) vs. 102(53)<br>HVD:92(48) vs. 91(47)<br>Unknown: 0	128(67) vs. 144(76)	none
CHAARTED [10, 36]	Grade 0, 69.5%<br>Grade 1-2, 30.5%	Median(IQR): 64(NR) vs. 63(NR)<br>Range: 36-91	Median(IQR): 50.9(NR) vs. 52.1(NR)<br>Range: 0.1-8540	≤7: 117(33) vs.104(30)<br>8-10: 241(67) vs.243(70)<br>Unknown: 46 vs. 39	57 (14) vs. 66 (17)	LVD:134(34) vs. 143(36)<br>HVD:263(66) vs. 250(64)<br>Unknown: 0	289(73) vs. 286(73)	none
STAMPEDE-arm C [11] [29]	Grade 0, 72.8%<br>Grade 1-2, 27.2%	Median(IQR): 65(60-70) vs. 65(60-71)<br>Range: NR	Median(IQR): 97(40.5-340) vs. 102.5(32.8-354)<br>Range: NR	≤7: 65(20) vs.158(25)<br>8-10: 253(80) vs. 480(75)<br>Unknown: 44 vs. 86	Lung:13(4) vs.33(5)<br>Liver: 6(2) vs. 15(2)<br>Other: 25(7) vs. 46(6)	LVD:124(46) vs. 238(43)<br>HVD:148(54) vs. 320(57)<br>Unknown: 90 vs. 166	Total: 1037(95)	none
STAMPEDE-arm G [12, 28]	Grade 0, 74.6%<br>Grade 1-2, 25.4%	Median(IQR): 67(NR) vs. 67(NR)<br>Range: NR	Median(IQR): 51(19-158)vs. 56(19-165)<br>Range: NR	≤7: 104(23) vs.110(24)<br>8-10: 345(77) vs.342(76)<br>Unknown: 101	22 (5) vs. 19 (4)	LVD:206(46) vs. 196(43)<br>HVD:243(54) vs. 256(57)<br>Unknown: 101	Total: 859(95)	none
STAMPEDE -arm H[17]	Grade 0, 71.1%<br>Grade 1-2, 28.9%	Median(IQR): 68(63-73) vs. 68(63-73)<br>Range: 37-87	Median(IQR): 97(30-313) vs. 98(30-316)<br>Range: 1-20590	≤7: 172(18) vs.173(17)<br>8-10: 810(82) vs.820(83)<br>Unknown: 36 vs. 50	Lung:48(5) vs. 42(4)<br>Liver: 19(2) vs. 23(2)<br>Other: 33(3) vs. 35(3)	LVD:410(43) vs.409(42)<br>HVD:553(57)vs. 567(58)<br>Unknown: 69 vs. 53<br>52% high in HORRAD definition	Total: 2061(100)	183(18) vs. 184(18)
LATITUDE [13, 34]	Grade 0-1, 96.7%<br>Grade 2, 3.3%	Median(IQR): 68(NR) vs. 67(NR)<br>Range: 33-92	Median(IQR): NR(NR) vs. NR(NR)<br>Range: NR	≤7: 13(2) vs.16(3)<br>8-10: 584(98) vs.586(97)<br>Unknown: 0	114 (19) vs. 114 (19)	LVD:110(18) vs. 133(22)<br>HVD:487(82) vs. 468(78)<br>Unknown: 0 vs 1	Total: 1199(100)	none
ENZAMET [14]	Grade 0, 72.0%<br>Grade 1-2, 28.0%	Median(IQR): 69(63-74) vs. 69(64-74)<br>Range: NR	Median(IQR): NR(NR) vs. NR(NR)<br>Range: NR	≤7: 152(31) vs.163(34)<br>8-10: 335(69) vs.321(66)<br>Unknown: 76 vs 78	62 (11) vs. 67 (12)	LVD:272(48) vs. 265(47)<br>HVD:291(52) vs. 297(53)<br>Unknown: 0	325(58) vs. 327(58)	254(45) vs. 249(44)

ARCHES [15, 31, 33]	Grade 0, 77.5%<br>Grade 1, 22.5%	Median(IQR): 70(NR) vs. 70(NR)<br>Range: 42-92	Median(IQR): 5.4(NR) vs. 5.1(NR)<br>Range: 0-19000	≤7: 171(31) vs.187(33)<br>8-10: 386(69) vs.373(67)<br>Unknown: 16 vs. 17	NR	LVD:220(38) vs. 203(35)<br>HVD:354(62) vs. 373(65)<br>Unknown: 0	402(83) vs. 365(81)<br>unknown: 89 vs. 125	103(18) vs. 102(18)
TITAN[16, 32]	Grade 0, 64.3%<br>Grade 1, 35.7%	Median(IQR): 69(NR) vs. 68(NR)<br>Range: 43-94	Median(IQR): 5.97(NR) vs. 4.02(NR)<br>Range: 0-2682	≤7: 174(33) vs.169(32)<br>8-10: 351(67) vs.358(68)<br>Unknown: 0	56 (11) vs. 72 (14)	LVD:200(38) vs. 192(36)<br>HVD:325(62) vs. 335(64)<br>Unknown: 0	411(83) vs. 441(88)<br>unknown: 29 vs. 27	58(11) vs. 55(10)
HORRAD[41]	Grade 0, 84.0%<br>Grade 1-3, 16.0%	Median(IQR): 67(61-71) vs. 67(61-71)<br>Range: 47-85	Median(IQR): 125(48-433) vs. 149(50-483)<br>Range: 4-14000	≤7: 73(34) vs.71(33)<br>8-10: 142(66) vs.144(67)<br>Unknown: 1 vs 1	NR	LVD: NR; HVD: NR<br>82% high in HORRAD definition	Total: 432(100)	none
PEACE-1 [23] (Docetaxel population)	Grade 0, 69.9%<br>Grade 1-2, 30.1%	Median(IQR): 66(60-70) vs. 66(59-70)	Median(IQR): 13.7 (2.4-58.9) vs. 12.0 (3.0-59.9)	≤7: 79(23) vs.71(20)<br>8-10:270(77) vs.276(80)	41(12) vs. 47(13)	LVD:131(37) vs. 123(35)<br>HVD: 224(63) vs. 232(65)	Total: 710(100)	none
ARASENS[24]	Grade 0, 71.1%<br>Grade 1, 28.9%	Median(Range): 67(41-89) vs. 67(42-86)	Median(Range): 30.3 (0-9219) vs. 24.2 (0-11947)	≤7: 122(18.7) vs.118(18)<br>8-10: 505(77.6) vs.516(78.9)	111(17) vs. 118(18)	NR	558(85.7) vs. 566(86.5)<br>unknown: 6 vs 7	none

Ctrl: control group; ECOG: Eastern Cooperative Oncology Group; Exp.: experimental group; HVD: high volume disease; IQR: interquartile range; LVD: low volume disease; NR: not reported; PSA: prostatic specific antigen.

Supplementary Material 4A: Hazard Ratio for OS and PFS in included trials or subgroups

STUDY No.[Ref.]	Trial	Comparison	Patients number	HR (OS -total)	HVD-pts number	HR (OS-HVD)	LVD-pts number	HR (OS-LVD)	Visceral meta-pts	HR (OS-visc.)	PFS type	HR(PFS -total)
1[10, 37]	GETUG -AFU15	DOC+ADT vs ADT	385	0.88, 0.68-1.14	183	0.78, 0.56-1.09	202	1.02, 0.67-1.55	NR	NR	rPFS<br>cPFS	0.69, 0.55-0.87<br>0.75, 0.59-0.94
2[11, 38]	CHAARTED	DOC+ADT vs ADT	790	0.72, 0.59-0.89	513	0.63, 0.50-0.79	277	1.04, 0.70-1.55	123 (15.6%)	0.53, 0.30-0.92	cPFS	0.62, 0.51-0.75
3[12] [31]	STAMPEDE -arm C	DOC+ADT vs ADT	1086a	0.81, 0.69-0.95	468	0.81, 0.64-1.02	362	0.76, 0.54-1.07	NR	NR	PFS	0.69, 0.59-0.81
4[14, 36]	LATITUDE	AAP+ADT vs ADT	1199	0.66, 0.56-0.78	955	0.62, 0.52-0.74	243	0.72, 0.47-1.10	228 (19.0%)	0.58, 0.41-0.83	rPFS	0.47, 0.39-0.55
5[13, 30]	STAMPEDE -arm G	AAP+ADT vs ADT	1002[a]	0.61, 0.49-0.75	499	0.60, 0.46-0.78	402	0.64, 0.42-0.97	NR	NR	PFS	0.45, 0.37-0.54
6[32]	STAMPEDE, AAP vs. DOC	AAP+ADT vs DOC+ADT	342[b]	1.13, 0.77-1.66	NR	NR	NR	NR	NR	NR	PFS	0.69, 0.50-0.95
7[15]	ENZAMET[c]	ENZA+ADT vs ADT	622	0.53, 0.37-0.75	232	0.65, 0.42-0.99	390	0.38, 0.21-0.69	129 (11.5%)	1.05, 0.54-2.02	cPFS	0.34, 0.26-0.44
8[15]	ENZAMET, DOC-sub	ENZA+DOC+ADT vs DOC+ ADT	503	0.90 , 0.62-1.31	356	0.97, 0.64- 1.46	147	0.65, 0.25- 1.71				0.48, 0.37-0.62
9[16, 33, 35]	ARCHES[c]	ENZA+ADT vs ADT	945	0.64, 0.51-0.81	727	0.66, 0.52-0.83	423	0.66, 0.43-1.03	128 (11.13%)	1.16, 0.67-2.00	rPFS	0.37, 0.28-0.49
10 [16, 33, 35]	ARCHES, DOC-sub	ENZA+DOC+ADT vs DOC+ ADT	205	0.74, 0.46-1.20								0.52, 0.30-0.89
11[17, 34]	TITAN[c]	APA+ADT vs ADT	939	0.61, 0.50-0.76	660	0.70, 0.56-0.88	392	0.52, 0.35-0.79	128 (12.2%)	0.76, 0.47-1.23	rPFS	0.48, 0.39-0.60
	TITAN, DOC-sub		113	1.12 0.59-2.12								
12[18]	STAMPEDE - arm H[c]	RT+ADT vs ADT	1691	0.93, 0.80-1.08	1120	1.07, 0.90-1.28	819	0.68, 0.52-0.90	NR	NR	PFS	0.96, 0.85-1.08
	STAMPEDE-arm H, DOC-sub		367	0.81 0.49-1.34								
13[43]	HORRAD	RT+ADT vs ADT	432	0.90, 0.70-1.14	NR	NR	NR	NR	NR	NR	NR	NR
14[25]	PEACE-1, DOC-sub	AAP+DOC+ADT vs DOC+ ADT	710	0.75, 0.59-0.95	456	0.72, 0.55-0.95	254	0.83, 0.50-1.38	88 (12.4%)	NR	rPFS	0.50, 0.40-0.62
15[26]	ARASENS	DARO+DOC+ADT vs DOC+ ADT	1305	0.68, 0.57-0.80	NR	NR	NR	NR	229 (17.5%)	0.79, 0.55-1.14	NR	NR

[a] only metastatic patients were included; [b] direct comparison of abiraterone versus docetaxel from the multi-arm, multi-stage STAMPEDE trial; [c] patients with prior or concomitant docetaxel chemotherapy were excluded. AAP: abiraterone and prednisone; cPFS: clinical progression free survival; DOC: docetaxel; DOC-sub: subgroup with docetaxel population and available for triplet therapy analyses; HR: hazard ratio; HVD: high volume disease; LVD: low volume disease; NO.: number; NR: not reported; OS: overall survival; PFS: progression free survival; Ref.: reference; rPFS: radiographic progression free survival; visc.: visceral metastasis.

Supplementary Material 4B: Hazard Ratio for OS in de novo and previous local treatment subgroups

STUDY No.	1	2	3	4	5	6	7	9	11	12	13	14	15
Trial	GETUG -AFU15	CHAART ED	STAMPE DE-arm C	LATITUD E	STAMPE DE-arm G	STAMPE DE, abir vs. doce	ENZAME T	ARCHES	TITAN	STAMPED E -RT	HORRAD	PEACE-1, doce-sub	ARASENS
Patients number	385	790	1776*	1199	1917*	342§	1125	1150	1052	2061	432	710	1305
HR (OS -total)	0.88, 0.68-1.14	0.72, 0.59-0.89	0.78, 0.66-0.93	0.66, 0.56-0.78	0.63, 0.52-0.76	1.13, 0.77-1.66	0.67, 0.52-0.86	0.66, 0.53-0.81	0.65, 0.53-0.79	0.92, 0.80-1.06	0.90, 0.70-1.14	0.75, 0.59-0.95	0.68, 0.57-0.80
De novo -pts number	144/190(76%) ADT:DOC 128/190(67%)	575/789 (73%)	1681/1776 (95%)	100%	1819/1917 (95%)	NA	652/1125 (58%)	890/1136 (78%)	852/996 (86%)	100%	100%	100%	1124/1292 (87%)
HR (OS-de novo)	0.93, 0.69–1.25	0.68, 0.54-0.85	0.78, 0.65-0.94	0.66, 0.56-0.78	0.61, 0.50-0.74	NA	0.65, 0.47-0.89	0.63, 0.50-0.79	0.68, 0.55-0.85	0.92, 0.80-1.06	0.90, 0.70-1.14	0.75, 0.59-0.95	0.71, 0.59-0.85
PLT-pts number	46/190(24%) ADT:DOC 62/190(33%)	214/789 (27%)	95/1776 (5%)	0	98/1917 (5%)	NA	473/1125 (42%)	246/1136 (22%)	144/996 (14%)	NA	NA	NA	168/1292 (13%)
HR (OS-PLT)	0.83, 0.47–1.47	0.97, 0.58–1.62	0.80, 0.26–2.48	NA	0.94, 0.35–2.52	NA	0.72, 0.47–1.09	0.71, 0.41–1.21	0.39, 0.22-0.69	NA	NA	NA	0.61, 0.35–1.05

*Metastatic and localized patients were all included; § Direct comparison of abiraterone versus docetaxel from the multi-arm, multi-stage STAMPEDE trial; doce-sub: subgroup with docetaxel population and avilable for triple treatment analysis. PLT: previous local treatment.

Supplementary Material 5: Consistency test of closed-loop studies

	comparison	p.value	CrI
1	d.ADT.DOC+ADT	0.162625	
2	-> direct		-0.23 (-0.38, -0.073)
3	-> indirect		-0.57 (-1.0, -0.11)
4	-> network		-0.26 (-0.41, -0.12)
5	d.ADT.AAP+ADT	0.173875	
6	-> direct		-0.45 (-0.63, -0.26)
7	-> indirect		-0.11 (-0.56, 0.34)
8	-> network		-0.41 (-0.57, -0.23)
9	d.DOC+ADT.AAP+ADT	0.156700	
10	-> direct		0.13 (-0.30, 0.55)
11	-> indirect		-0.22 (-0.46, 0.020)
12	-> network		-0.15 (-0.34, 0.076)

Supplementary Material 6: Heterogeneity test

Heterogeneity test for OS for all population

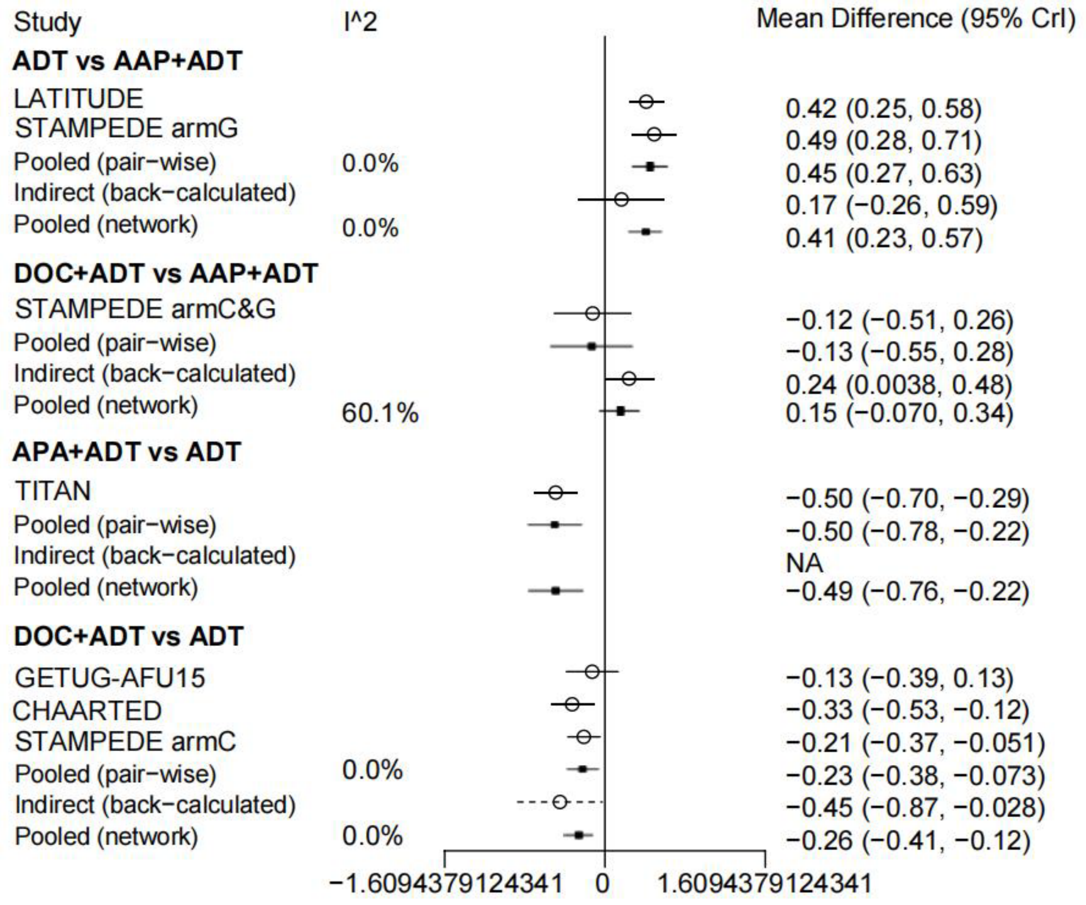

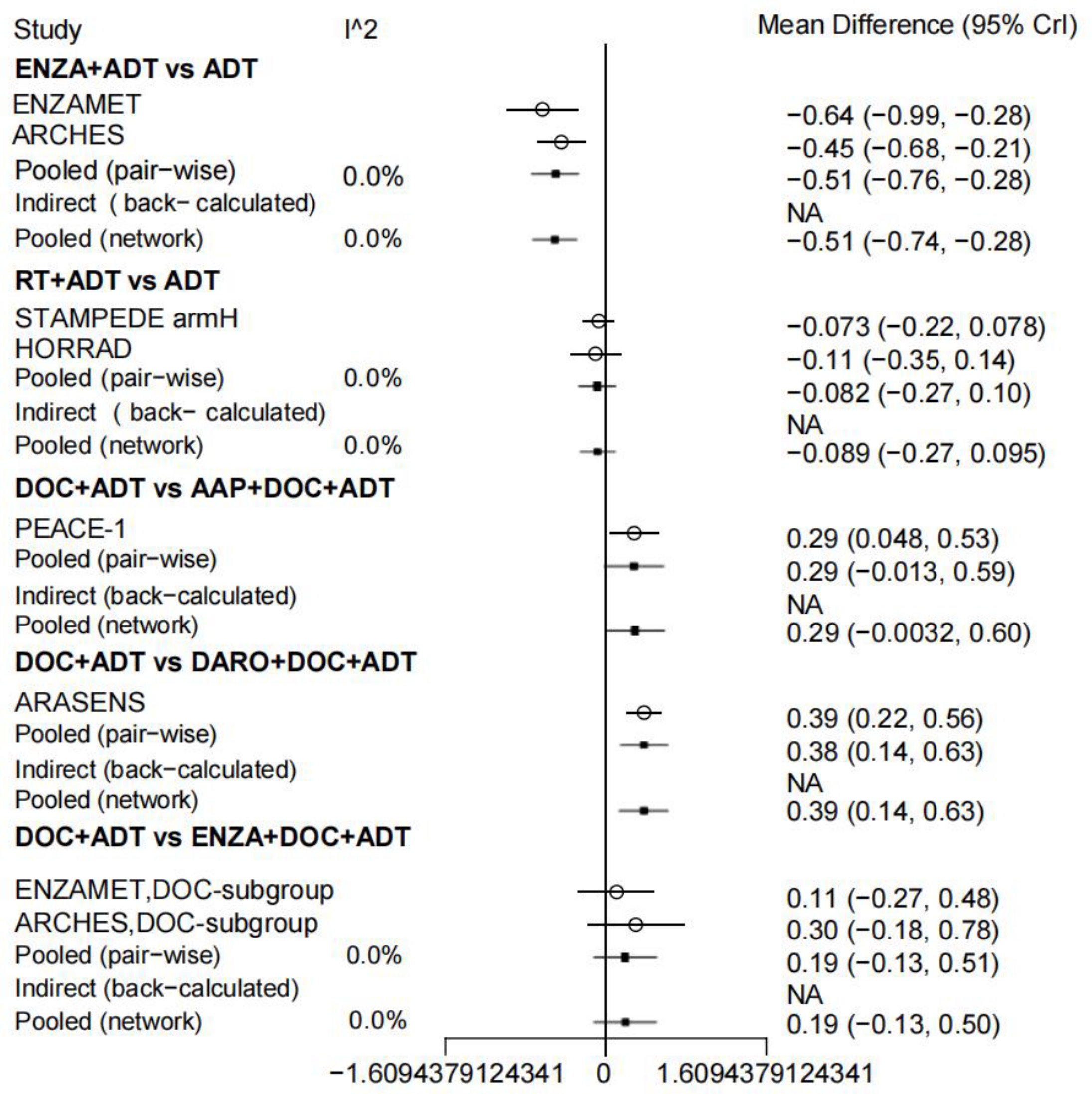
Study
I^2
Mean Difference (95% CrI)
ENZA+ADT vs ADT
ENZAMET −0.64 (−0.99, −0.28)
ARCHES −0.45 (−0.68, −0.21)
Pooled (pair−wise) 0.0% −0.51 (−0.76, −0.28)
Indirect (back− calculated) NA
Pooled (network) 0.0% −0.51 (−0.74, −0.28)
RT+ADT vs ADT
STAMPEDE armH −0.073 (−0.22, 0.078)
HORRAD −0.11 (−0.35, 0.14)
Pooled (pair−wise) 0.0% −0.082 (−0.27, 0.10)
Indirect (back− calculated) NA
Pooled (network) 0.0% −0.089 (−0.27, 0.095)
DOC+ADT vs AAP+DOC+ADT
PEACE-1 0.29 (0.048, 0.53)
Pooled (pair−wise) 0.29 (−0.013, 0.59)
Indirect (back−calculated) NA
Pooled (network) 0.29 (−0.0032, 0.60)
DOC+ADT vs DARO+DOC+ADT
ARASENS 0.39 (0.22, 0.56)
Pooled (pair−wise) 0.38 (0.14, 0.63)
Indirect (back−calculated) NA
Pooled (network) 0.39 (0.14, 0.63)
DOC+ADT vs ENZA+DOC+ADT
ENZAMET,DOC-subgroup 0.11 (−0.27, 0.48)
ARCHES,DOC-subgroup 0.30 (−0.18, 0.78)
Pooled (pair−wise) 0.0% 0.19 (−0.13, 0.51)
Indirect (back−calculated) NA
Pooled (network) 0.0% 0.19 (−0.13, 0.50)
−1.6094379124341
0
1.6094379124341

Heterogeneity test for PFS

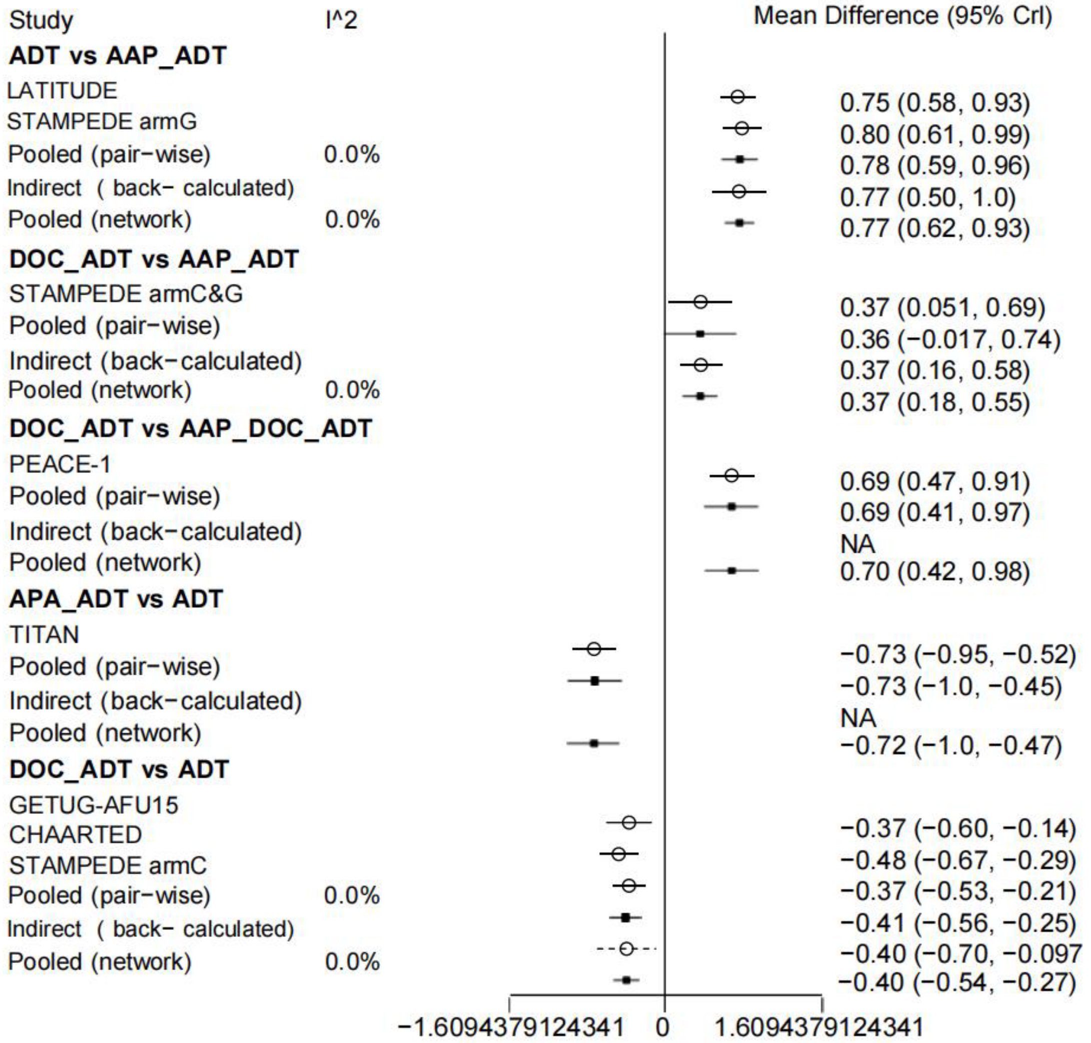

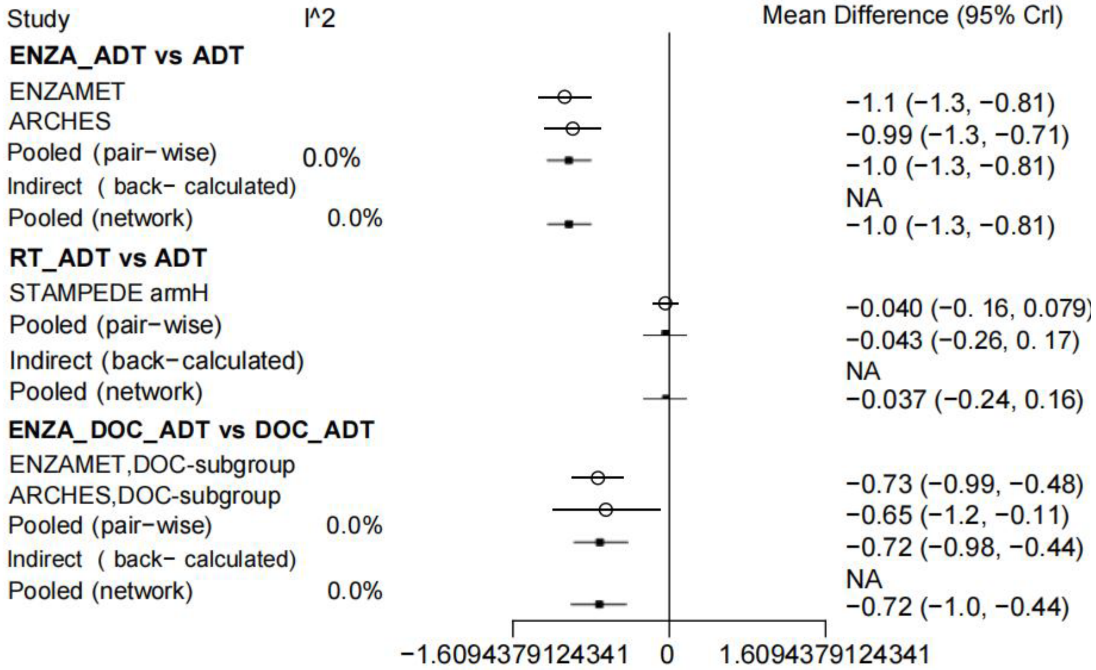

Heterogeneity test for OS-HVD

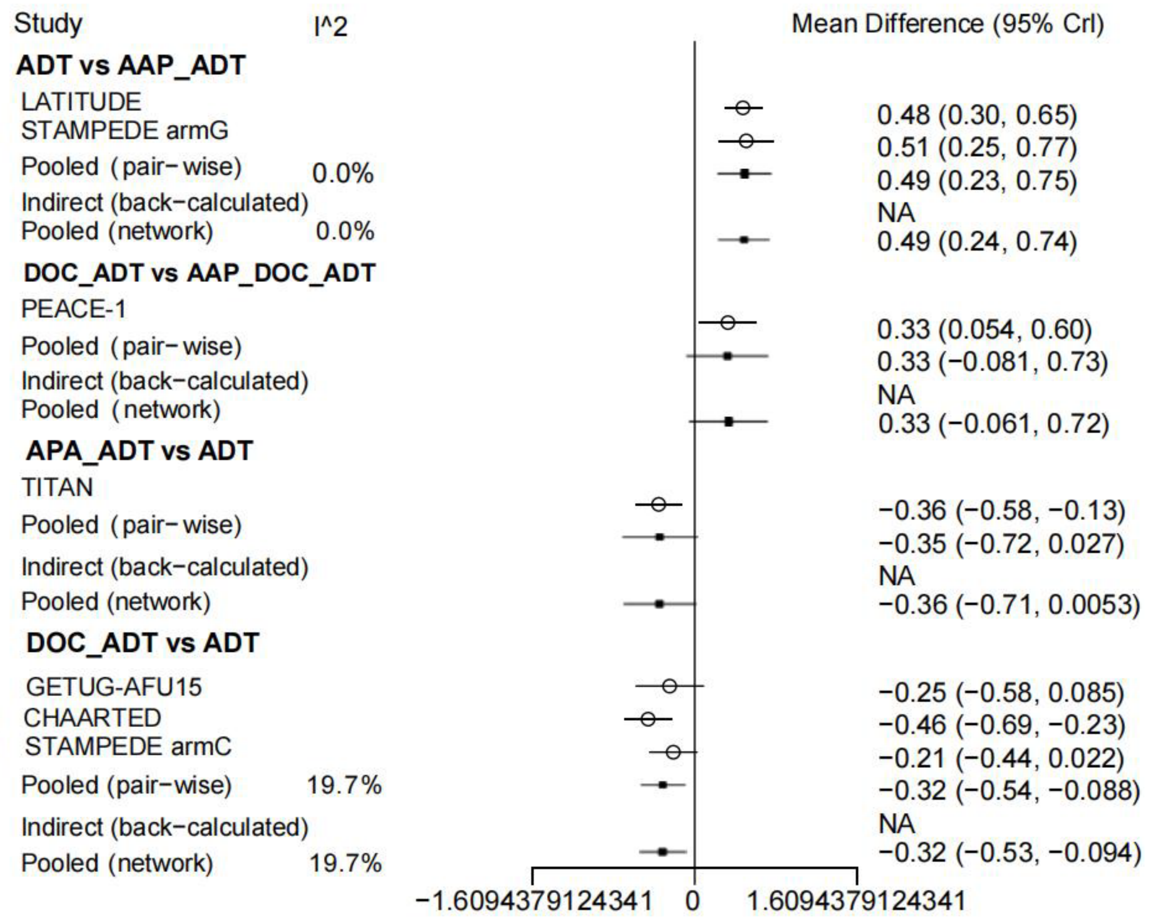

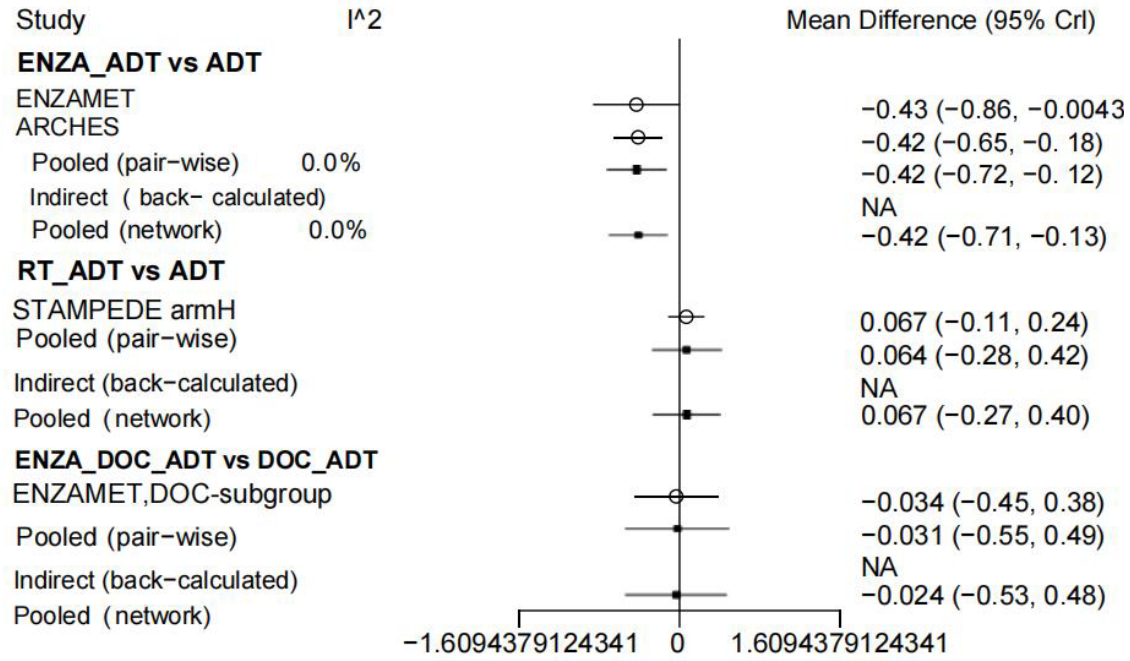

Heterogeneity test for OS-LVD

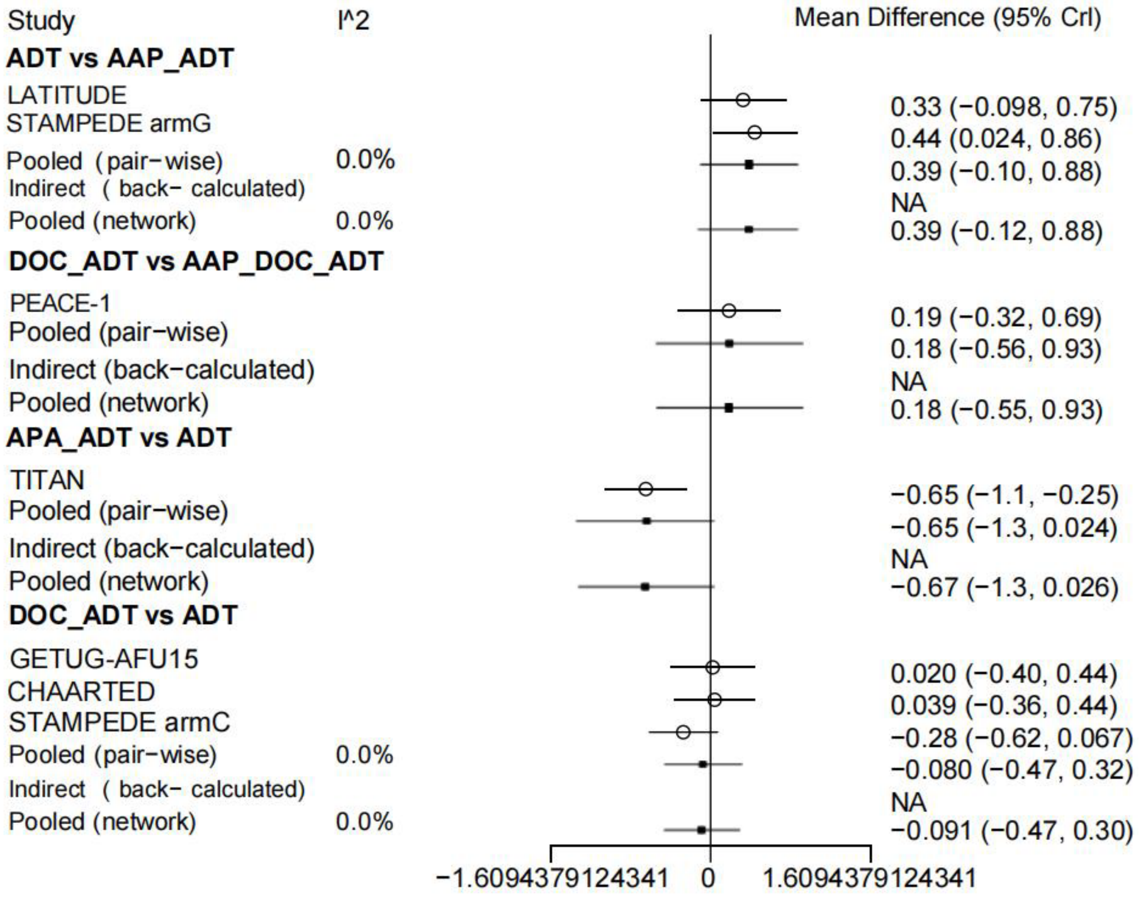

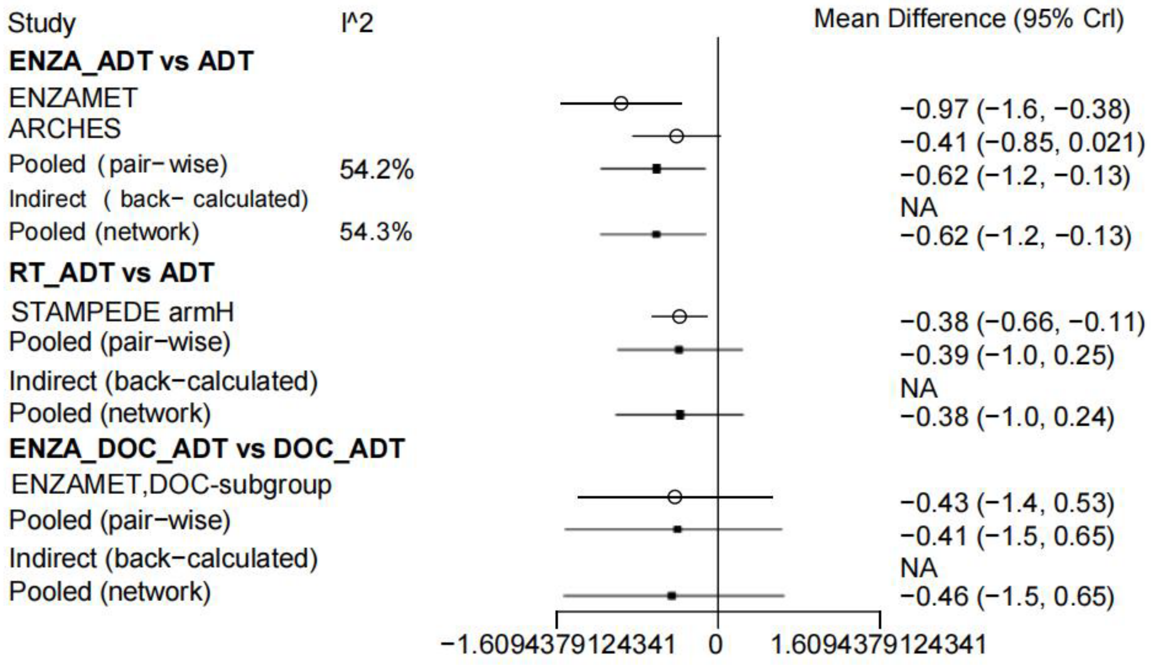

Heterogeneity test for OS-visceral metastasis

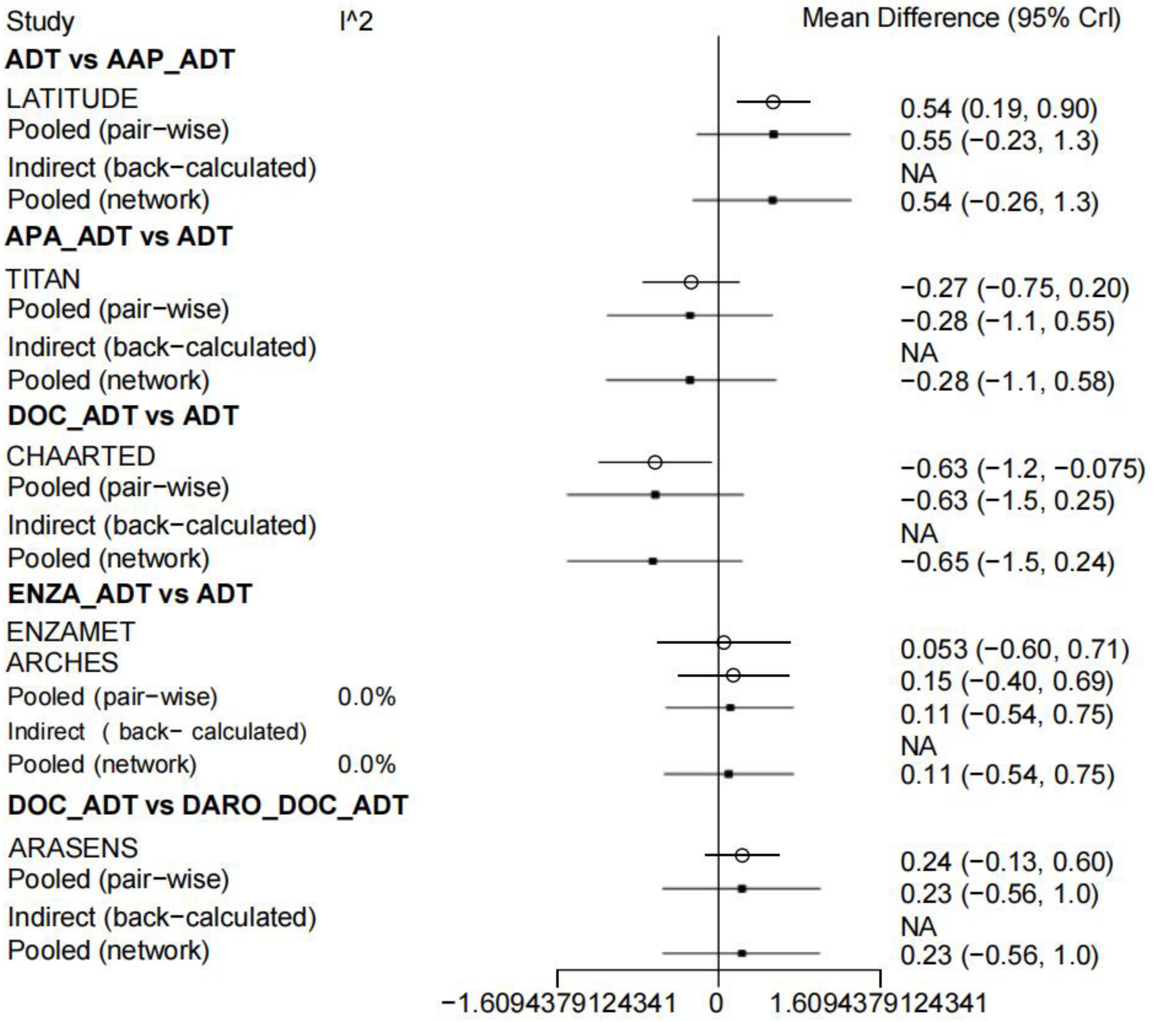

Heterogeneity test for high grade AE

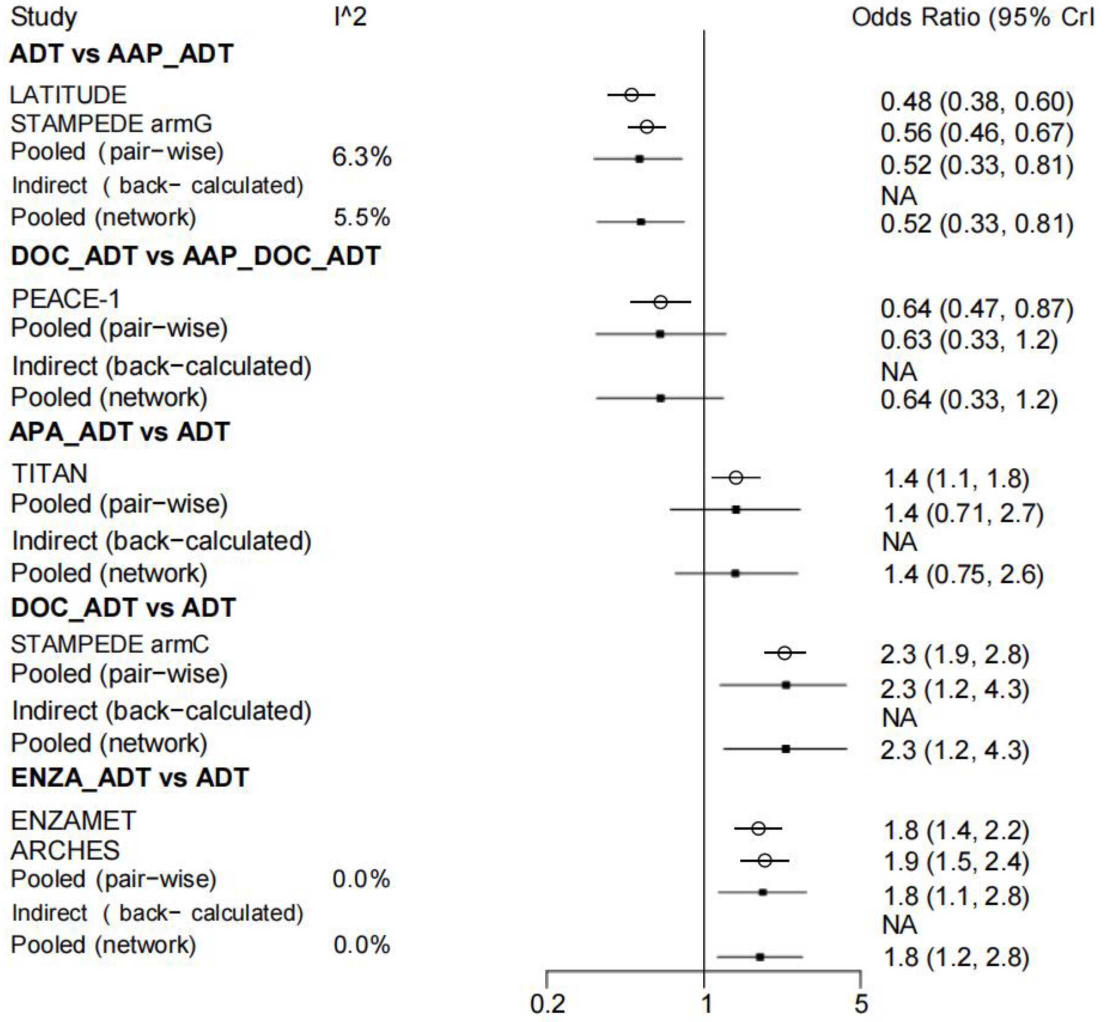

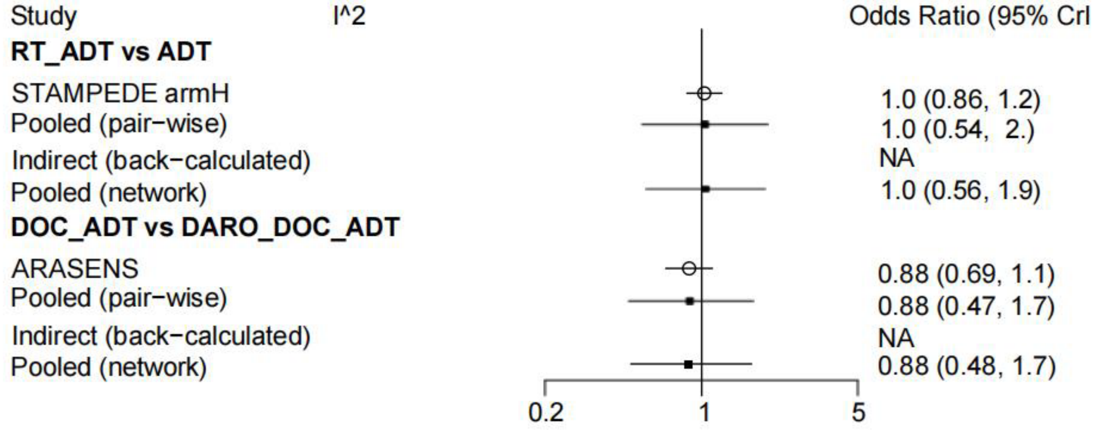

Supplementary materials 7: Supplementary forest plots (for de novo and previous local treatment patients, as well as respective analyses for rPFS, PFS and cPFS)

ADT: androgen deprivation treatment; DOC: docetaxel; AAP: abiraterone acetate and prednisone; ENZA: enzalutamide; APA: apalutamide; RT: radiotherapy; DARO: darolutamide. OS: overall survival; PFS: progression free survival; cPFS: clinical progression free survival; rPFS: radiographic progression free survival; The connector line "_" represents combination of different treatment regimens.

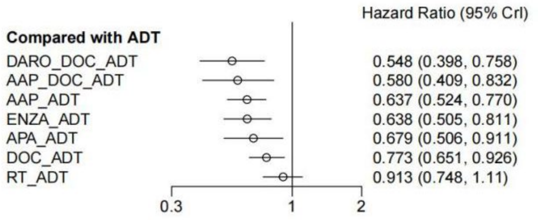

A: OS for de novo disease (vs. ADT)

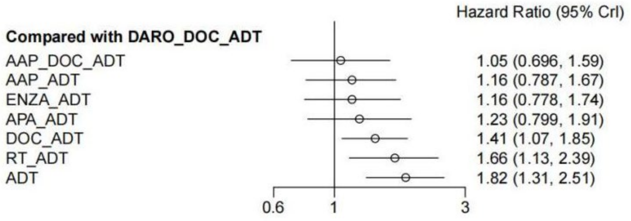

B: OS for de novo disease (vs. DARO_DOC_ADT)

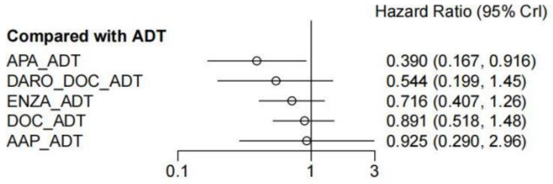

C: OS for patients with previous local treatment (vs. ADT)

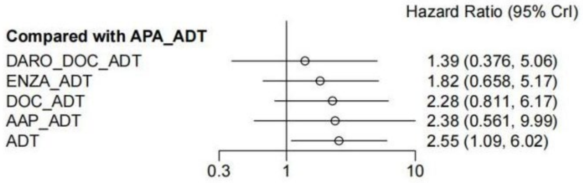

D: OS for patients with previous local treatment (vs. APA_ADT)

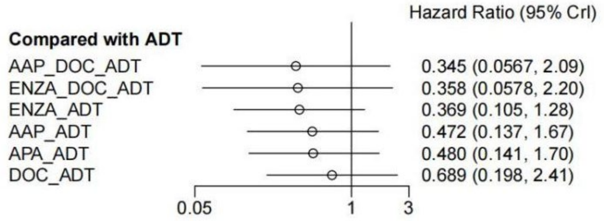

E: rPFS (vs.ADT)

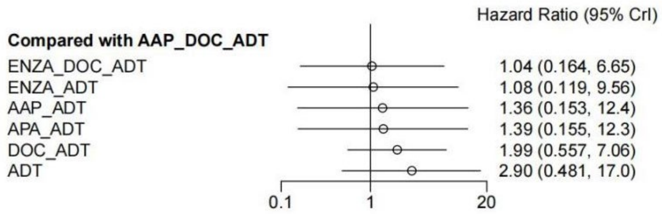

F: rPFS (vs.ENZA_DOC_ADT)

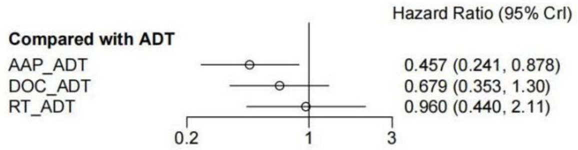

G: PFS (vs.ADT)

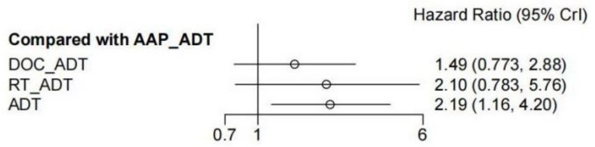

H: PFS (vs.AAP)

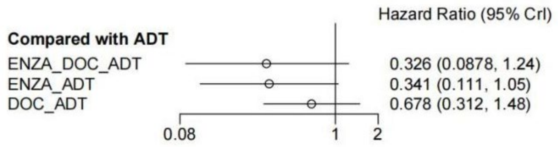

I: cPFS (vs.ADT)

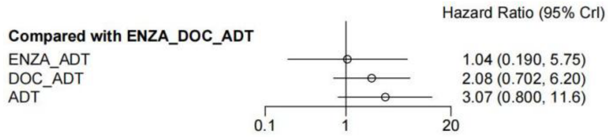

J: cPFS (vs.ENZA_DOC_ADT)

Supplementary Material 8: Relative effect estimates for all possible treatment comparisons

Overall survival, hazard ratio (95% CI)

DARO+DOC+ADT								
0.91 (0.61, 1.34)	AAP+DOC+ADT							
0.81 (0.54, 1.23)	0.90 (0.58, 1.40)	ENZA+DOC+ADT						
**0.57 (0.40, 0.80)**	**0.63 (0.43, 0.92)**	0.70 (0.46, 1.03)	RT+ADT					
0.86 (0.57, 1.29)	0.95 (0.61, 1.47)	1.05 (0.67, 1.65)	1.51 (1.07, 2.12)	APA+ADT				
0.87 (0.60, 1.26)	0.96 (0.64, 1.45)	1.07 (0.70, 1.64)	1.53 (1.14, 2.06)	1.01 (0.71, 1.47)	ENZA+ADT			
0.79 (0.56, 1.08)	0.87 (0.60, 1.24)	0.97 (0.65, 1.41)	1.38 (1.06, 1.76)	0.92 (0.66, 1.26)	0.90 (0.67, 1.20)	AAP+ADT		
**0.68 (0.53, 0.88)**	0.75 (0.55, 1.01)	0.83 (0.60, 1.14)	1.20 (0.95, 1.52)	0.79 (0.58, 1.10)	0.78 (0.59, 1.04)	0.86 (0.71, 1.09)	DOC+ADT	
**0.52 (0.39, 0.70)**	**0.58 (0.41, 0.81)**	**0.64 (0.45, 0.92)**	0.92 (0.76, 1.11)	**0.61 (0.46, 0.81)**	**0.60 (0.47, 0.76)**	**0.67 (0.57, 0.80)**	**0.77 (0.66, 0.89)**	Placebo/No treatment /NSAA+ADT

Generalized progression-free survival, hazard ratio (95% CI)

AAP+DOC+ADT							
1.02 (0.70, 1.48)	ENZA+DOC+ADT						
**0.35 (0.24, 0.49)**	**0.34 (0.24, 0.48)**	RT+ADT					
0.69 (0.47, 1.03)	0.68 (0.45, 1.01)	2.00 (1.45, 2.77)	APA+ADT				
0.94 (0.65, 1.36)	0.92 (0.64, 1.34)	2.72 (2.03, 3.63)	1.36 (0.97, 1.91)	ENZA+ADT			
**0.72 (0.52, 0.99)**	**0.71 (0.51, 0.97)**	2.08 (1.64, 2.66)	1.05 (0.77, 1.42)	0.77 (0.59, 1.00)	AAP+ADT		
**0.50 (0.38, 0.65)**	**0.49 (0.37, 0.64)**	1.44 (1.14, 1.83)	**0.72 (0.54, 0.96)**	**0.53 (0.41, 0.68)**	**0.69 (0.58, 0.83)**	DOC+ADT	
**0.33 (0.25, 0.45)**	**0.32 (0.24, 0.44)**	0.96 (0.80, 1.16)	**0.48 (0.37, 0.63)**	**0.35 (0.28, 0.44)**	**0.46 (0.40, 0.53)**	**0.67 (0.59, 0.76)**	Placebo/No treatment /NSAA+ADT

Overall survival for patients with high volume disease, hazard ratio (95% CI)

AAP+DOC+ADT							
0.75 (0.39, 1.40)	ENZA+DOC+ADT						
**0.49 (0.28, 0.87)**	0.66 (0.35, 1.28)	RT+ADT					
0.75 (0.42, 1.34)	1.01 (0.52, 0.99)	1.52 (0.93, 2.54)	APA+ADT				
0.80 (0.47, 1.38)	1.08 (0.58, 2.01)	**1.63 (1.05, 2.55)**	1.06 (0.67, 1.71)	ENZA+ADT			
0.85 (0.51, 1.46)	1.15 (0.63, 2.11)	**1.75 (1.14, 2.70)**	1.14 (0.73, 1.78)	1.07 (0.72, 1.60)	AAP+ADT		
0.72 (0.49, 1.08)	0.97 (0.59, 1.61)	1.47 (0.96, 2.20)	0.96 (0.62, 1.48)	0.90 (0.62, 1.31)	0.84 (0.59, 1.18)	DOC+ADT	
**0.52 (0.33, 0.83)**	0.71 (0.41, 1.22)	1.07 (0.76, 1.51)	0.70 (0.48, 1.02)	**0.66 (0.49, 0.89)**	**0.61 (0.47, 0.79)**	**0.73 (0.58, 0.91)**	Placebo/No treatment /NSAA+ADT

Overall survival for patients with low volume disease, hazard ratio (95% CI)

AAP+DOC+ADT							
1.26 (0.33, 4.74)	ENZA+DOC+ADT						
1.13 (0.40, 3.21)	0.88 (0.24, 3.38)	RT+ADT					
1.47 (0.50, 4.34)	1.15 (0.30, 4.40)	1.30 (0.52, 3.38)	APA+ADT				
1.42 (0.54, 4.06)	1.11 (0.32, 4.20)	1.27 (1.57, 3.07)	0.97 (0.42, 2.45)	ENZA+ADT			
1.12 (0.43, 2.95)	0.87 (0.25, 3.13)	1.00 (0.45, 2.24)	0.77 (0.34, 1.78)	0.79 (0.37, 1.58)	AAP+ADT		
0.83 (0.40, 1.75)	0.65 (0.22, 1.94)	0.74 (0.35, 1.56)	0.57 (0.25, 1.25)	0.58 (0.30, 1.08)	0.74 (0.39, 1.37)	DOC+ADT	
0.77 (0.33, 1.78)	0.60 (0.19, 1.92)	0.68 (0.36, 1.28)	0.52 (0.26, 1.05)	**0.53 (0.31, 0.88)**	0.68 (0.41, 1.13)	0.92 (0.62, 1.37)	Placebo/No treatment /NSAA+ADT

Overall survival for patients with visceral metastasis, hazard ratio (95% CI)

DARO+DOC+ADT					
0.55 (0.13, 2.29)	APA+ADT				
0.38 (0.10, 1.44)	0.69 (0.24, 1.96)	ENZA+ADT			
0.73 (0.18, 3.02)	1.31 (0.43, 4.15)	1.91 (0.70, 5.18)	AAP+ADT		
0.79 (0.37, 1.74)	1.43 (0.42, 4.77)	2.11 (0.71, 6.07)	1.10 (0.34, 3.50)	DOC+ADT	
0.42 (0.13, 1.34)	0.76 (0.33, 1.75)	1.11 (0.58, 2.09)	0.58 (0.27, 1.26)	0.53 (0.22, 1.27)	Placebo/No treatment /NSAA+ADT

High grade AE, Odds ratio (95% CI)

DARO+DOC+ADT							
0.73 (0.31, 1.73)	AAP+DOC+ADT						
2.53 (0.84,6.90)	**3.46 (1.19, 9.92)**	RT+ADT					
1.85 (0.65, 5.17)	2.55 (0.90, 7.22)	0.73 (0.31, 1.64)	APA+ADT				
1.44 (0.56, 3.75)	1.98 (0.78, 5.27)	0.57 (0.28, 1.18)	0.78 (0.38, 1.62)	ENZA+ADT			
1.36 (0.53, 3.34)	1.86 (0.71, 4.66)	0.54 (0.26, 1.08)	0.73 (0.35, 1.49)	0.94 (0.52, 1.69)	AAP+ADT		
1.13 (0.63, 2.03)	1.56 (0.86, 2.85)	0.45 (0.20, 1.03)	0.61 (0.27, 1.37)	0.79 (0.39, 1.61)	0.84 (0.43, 1.72)	DOC+ADT	
**2.59 (1.11,6.09)**	**3.56 (1.51, 8.43)**	1.03 (0.57, 1.87)	1.39 (0.76, 2.55)	**1.81 (1.18, 2.77)**	**1.92 (1.27, 2.95)**	**2.28 (1.26, 4.16)**	Placebo/No treatment /NSAA+ADT

Overall survival for patients with de novo disease, hazard ratio (95% CI)

DARO+DOC+ADT							
0.95(0.63, 1.44)	AAP+DOC+ADT						
0.86 (0.59, 1.26)	0.91(0.61, 1.38)	AAP+ADT					
0.86 (0.58, 1.30)	0.91 (0.60, 1.40)	0.997 (0.73, 1.35)	ENZA+ADT				
0.81 (0.53, 1.26)	0.86 (0.54, 1.36)	0.94 (0.66, 1.33)	0.94 (0.64, 1.38)	APA+ADT			
**0.71 (0.54, 0.94)**	0.75 (0.55, 1.02)	0.83 (0.63, 1.06)	0.823 (0.61, 1.11)	0.88 (0.62, 1.24)	DOC+ADT		
**0.60 (0.41, 0.89)**	**0.64 (0.43, 0.96)**	**0.70 (0.53, 0.92)**	**0.70 (0.52, 0.95)**	0.74(0.52, 1.07)	0.85 (0.66, 1.11)	RT+ADT	
**0.55 (0.40, 0.76)**	**0.58 (0.41, 0.83)**	**0.64 (0.52, 0.77)**	**0.64 (0.51, 0.81)**	**0.68 (0.51, 0.91)**	**0.77 (0.65, 0.93)**	0.91 (0.75, 1.11)	Placebo/No treatment /NSAA+ADT

Overall survival for patients with previous local treatment, hazard ratio (95% CI)

APA+ADT					
0.72 (0.19, 2.71)	DARO+DOC+ADT				
0.54 (0.20, 1.50)	0.76 (0.24, 2.33)	ENZA+ADT			
0.44 (0.16, 1.20)	0.61 (0.26, 1.42)	0.81 (0.38, 1.74)	DOC+ADT		
0.41 (0.10, 1.80)	0.58 (0.13, 2.65)	0.76 (0.21, 2.78)	0.95 (0.26, 3.30)	AAP+ADT	
**0.39 (0.17, 0.92)**	0.54 (0.20, 1.45)	0.72 (0.41, 1.26)	0.89 (0.52, 1.48)	0.93 (0.29, 2.96)	Placebo/No treatment /NSAA+ADT

AAP: abiraterone acetate and prednisone; ADT: androgen deprivation therapy; APA: apalutamide; CI: confidence interval; DARO: darolutamide; DOC: docetaxel; ENZA: enzalutamide; NSAA: non-steroidal anti-androgen, including nilutamide, flutamide or bicalutamide; RT: radiotherapy.

Supplementary Material 9: OS/PFS ranking and ranking probability of competing interventions based on overall population

A. OS for overall population

Probability for place		1st	2nd	3rd	4th	5th	6th	7th	8th	9th
Rank	Treatment									
1	DARO+DOC+ADT	0.49	0.26	0.13	0.07	0.03	0.01	0	0	0
2	AAP+DOC+ADT	0.20	0.23	0.20	0.15	0.11	0.08	0.02	0	0
3	ENZA+ADT	0.11	0.19	0.24	0.21	0.15	0.08	0.02	0	0
4	APA+ADT	0.11	0.16	0.20	0.20	0.17	0.08	0.02	0	0
5	ENZA+DOC+ADT	0.08	0.12	0.15	0.16	0.17	0.20	0.10	0.03	0.01
6	AAP+ADT	0.01	0.03	0.09	0.20	0.32	0.28	0.06	0.01	0
7	DOC+ADT	0	0	0	0.01	0.04	0.22	0.68	0.04	0
8	RT+ADT	0	0	0	0	0.01	0.02	0.07	0.74	0.16
9	ADT alone	0	0	0	0	0	0	0	0.17	0.83

B. Generalized PFS for overall population

Probability for place		1st	2nd	3rd	4th	5th	6th	7th	8th
Rank	Treatment								
1	ENZA+DOC+ADT	0.45	0.33	0.20	0.02	0.01	0	0	0
2	AAP+DOC+ADT	0.36	0.35	0.25	0.03	0.01	0	0	0
3	ENZA+ADT	0.19	0.30	0.46	0.04	0.01	0	0	0
4	AAP+ADT	0	0.01	0.04	0.59	0.37	0	0	0
5	APA+ADT	0	0.01	0.01	0.32	0.59	0.02	0	0
6	DOC+ADT	0	0	0	0	0.02	0.98	0	0
7	RT+ADT	0	0	0	0	0	0	0.69	0.31
8	ADT alone	0	0	0	0	0	0	0.31	0.69

C. OS for patients with high volume disease

Probability for place		1st	2nd	3rd	4th	5th	6th	7th	8th
Rank	Treatment								
1	AAP+DOC+ADT	0.61	0.18	0.10	0.06	0.03	0.02	0	0
2	AAP+ADT	0.15	0.33	0.26	0.15	0.07	0.03	0	0
3	ENZA+ADT	0.08	0.19	0.24	0.21	0.16	0.11	0.01	0
4	APA+ADT	0.06	0.11	0.16	0.20	0.19	0.23	0.03	0.01
5	ENZA+DOC+ADT	0.10	0.16	0.12	0.13	0.14	0.23	0.06	0.06
6	DOC+ADT	0	0.02	0.10	0.24	0.38	0.24	0.01	0
7	ADT alone	0	0	0	0	0.01	0.08	0.65	0.25
8	RT+ADT	0	0	0.01	0.01	0.02	0.06	0.24	0.66

D. OS for patients with low volume disease

Probability for place		1st	2nd	3rd	4th	5th	6th	7th	8th
Rank	Treatment								
1	APA+ADT	0.31	0.26	0.17	0.23	0.07	0.04	0.02	0.02
2	ENZA+ADT	0.24	0.30	0.22	0.13	0.07	0.03	0.01	0
3	ENZA+DOC+ADT	0.29	0.13	0.11	0.10	0.09	0.08	0.06	0.14
4	AAP+ADT	0.04	0.10	0.19	0.24	0.22	0.12	0.06	0.02
5	RT+ADT	0.05	0.11	0.18	0.23	0.20	0.12	0.06	0.04
6	AAP+DOC+ADT	0.06	0.09	0.11	0.14	0.16	0.16	0.11	0.16
7	DOC+ADT	0	0	0.01	0.04	0.13	0.29	0.38	0.15
8	ADT alone	0	0	0	0.01	0.06	0.16	0.30	0.47

E. OS for patients with vesical metastasis

Probability for place		1st	2nd	3rd	4th	5th	6th
Rank	Treatment						
1	DARO+DOC+ADT	0.59	0.20	0.11	0.05	0.03	0.03
2	DOC+ADT	0.11	0.46	0.26	0.11	0.04	0.02
3	AAP+ADT	0.21	0.20	0.35	0.16	0.04	0.03
4	APA+ADT	0.08	0.11	0.19	0.35	0.13	0.13
5	ADT alone	0	0	0.04	0.18	0.53	0.24
6	ENZA+ADT	0.01	0.02	0.05	0.14	0.23	0.55

F. Grade 3-5 AEs for overall population

Probability for place		1st	2nd	3rd	4th	5th	6th	7th	8th
Rank	Treatment								
1	ADT alone	0.51	0.42	0.05	0.01	0	0	0	0
2	RT+ADT	0.41	0.44	0.10	0.03	0.01	0.01	0.01	0
3	APA+ADT	0.05	0.09	0.64	0.11	0.05	0.02	0.02	0.01
4	ENZA+ADT	0.01	0.02	0.10	0.46	0.27	0.08	0.05	0.02
5	AAP+ADT	0	0.01	0.06	0.27	0.44	0.12	0.08	0.03
6	DOC+ADT	0	0.01	0.02	0.06	0.14	0.58	0.18	0.01
7	DARO+DOC+ADT	0.01	0.01	0.02	0.05	0.07	0.16	0.55	0.13
8	AAP+DOC+ADT	0	0	0.01	0.01	0.02	0.03	0.12	0.80

G. OS for de novo patients

Probability for place		1st	2nd	3rd	4th	5th	6th	7th	8th
Rank	Treatment								
1	DARO+DOC+ADT	0.48	0.28	0.12	0.07	0.04	0.01	0	0
2	AAP+DOC+ADT	0.30	0.29	0.16	0.12	0.09	0.02	0.01	0
3	AAP+ADT	0.07	0.16	0.30	0.29	0.16	0.03	0	0
4	ENZA+ADT	0.10	0.17	0.25	0.25	0.18	0.05	0.01	0
5	APA+ADT	0.06	0.10	0.16	0.21	0.29	0.15	0.03	0.01
6	DOC+ADT	0	0	0.01	0.06	0.21	0.65	0.07	0
7	RT+ADT	0	0	0	0.01	0.02	0.09	0.74	0.14
8	ADT alone	0	0	0	0	0	0.01	0.15	0.84

H. OS for patients with previous local treatment

Probability for place		1st	2nd	3rd	4th	5th	6th
Rank	Treatment						
1	APA+ADT	0.65	0.23	0.07	0.03	0.01	0.01
2	DARO+DOC+ADT	0.25	0.39	0.19	0.09	0.05	0.04
3	ENZA+ADT	0.04	0.21	0.37	0.24	0.10	0.04
4	DOC+ADT	0	0.04	0.17	0.32	0.29	0.17
5	ADT alone	0	0.01	0.04	0.18	0.44	0.33
6	AAP+ADT	0.06	0.13	0.15	0.14	0.11	0.41

AAP: abiraterone acetate and prednisone; ADT: androgen deprivation therapy; APA: apalutamide; DARO: darolutamide; DOC: docetaxel; ENZA: enzalutamide; OS: overall survival; PFS: progression free survival; RT: radiotherapy.

Supplementary Material 10: Trace and density plots

A: Trace and density plots for OS for all population

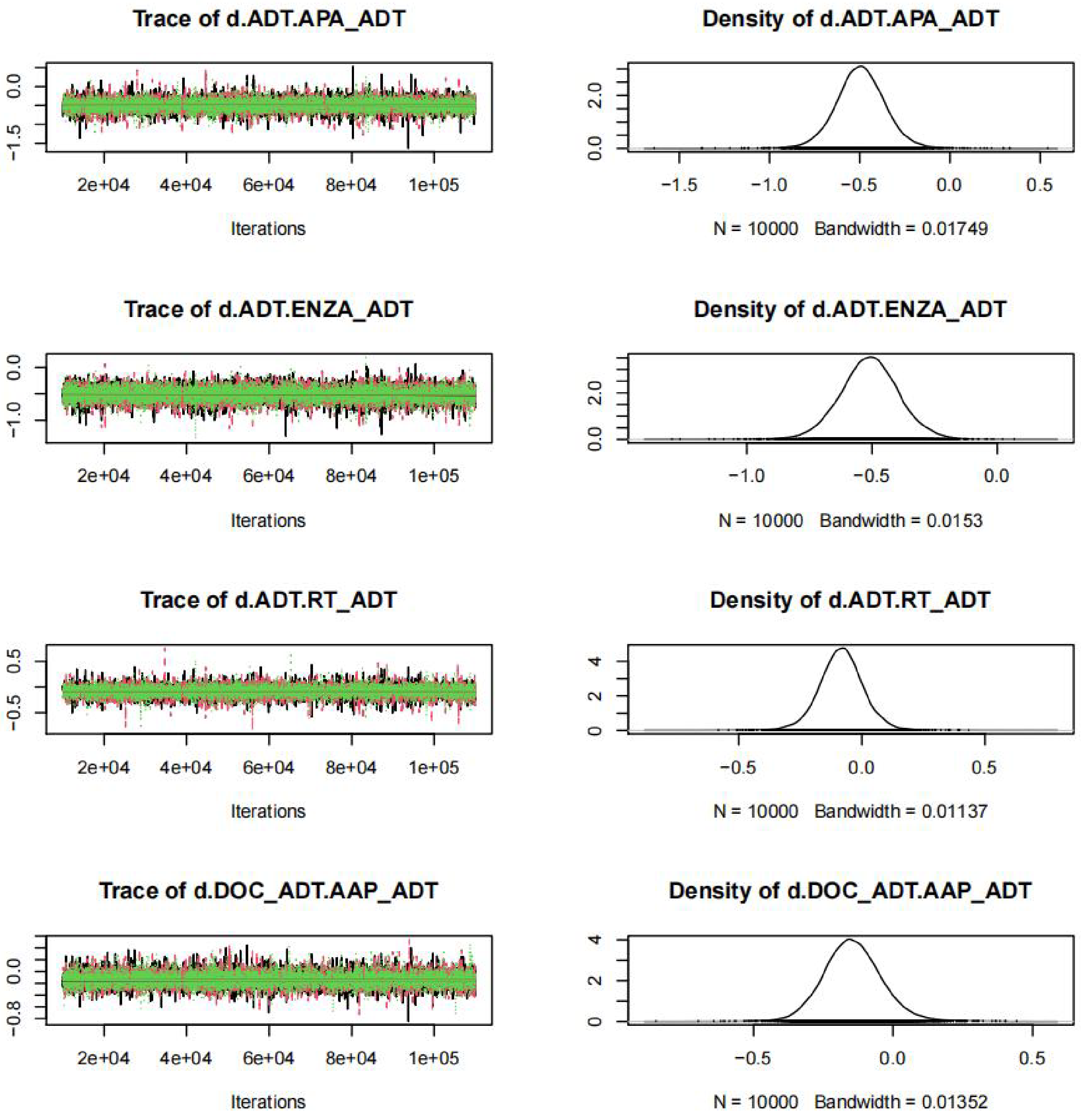

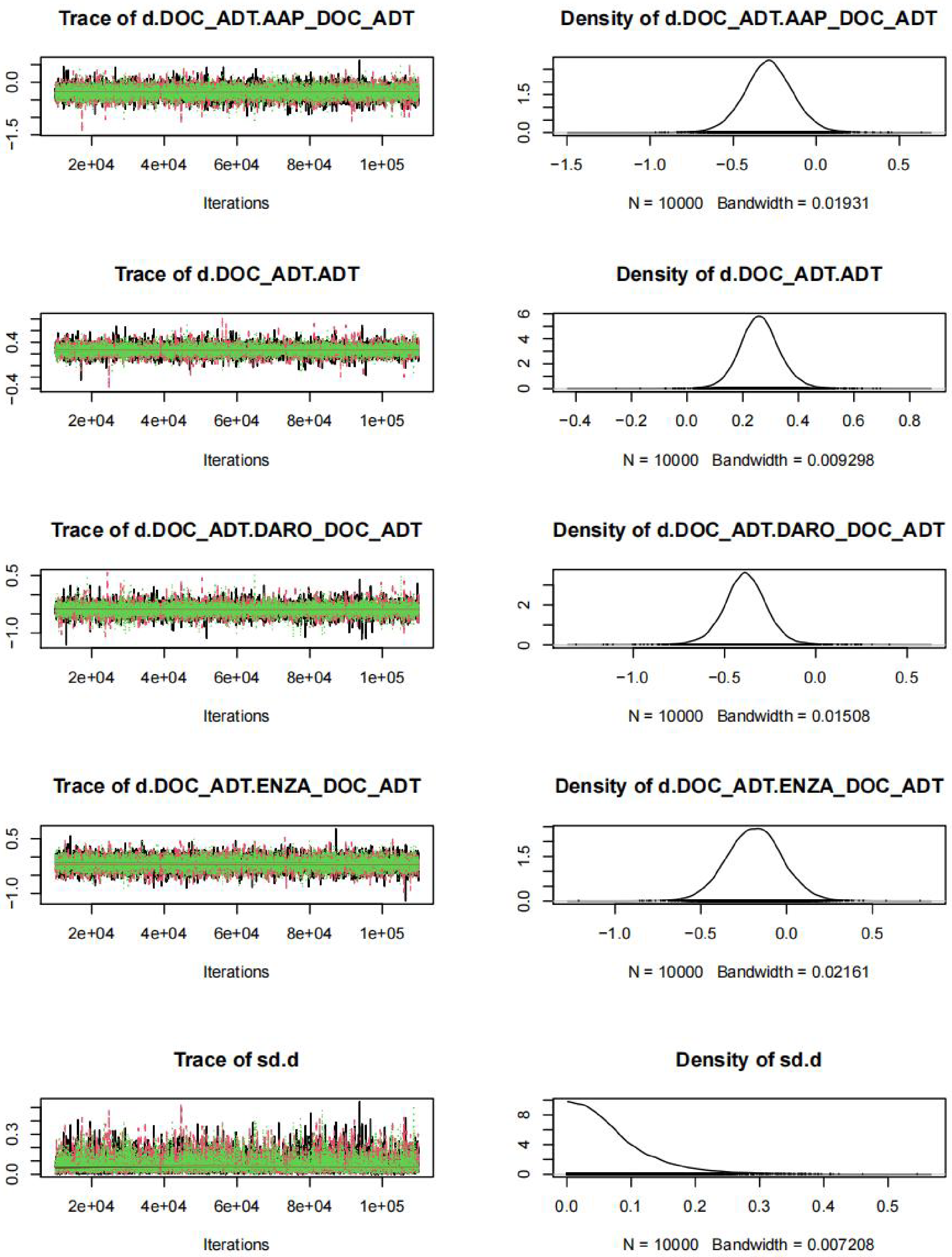
Trace of d.DOC_ADT.AAP_DOC_ADT
Density of d.DOC_ADT.AAP_DOC_ADT
Iterations
N = 10000 Bandwidth = 0.01931
Trace of d.DOC_ADT.ADT
Density of d.DOC_ADT.ADT
Iterations
N = 10000 Bandwidth = 0.009298
Trace of d.DOC_ADT.DARO_DOC_ADT
Density of d.DOC_ADT.DARO_DOC_ADT
Iterations
N = 10000 Bandwidth = 0.01508
Trace of d.DOC_ADT.ENZA_DOC_ADT
Density of d.DOC_ADT.ENZA_DOC_ADT
Iterations
N = 10000 Bandwidth = 0.02161
Trace of sd.d
Density of sd.d
Iterations
N = 10000 Bandwidth = 0.007208

B: Trace and density plots for generalized PFS for all population

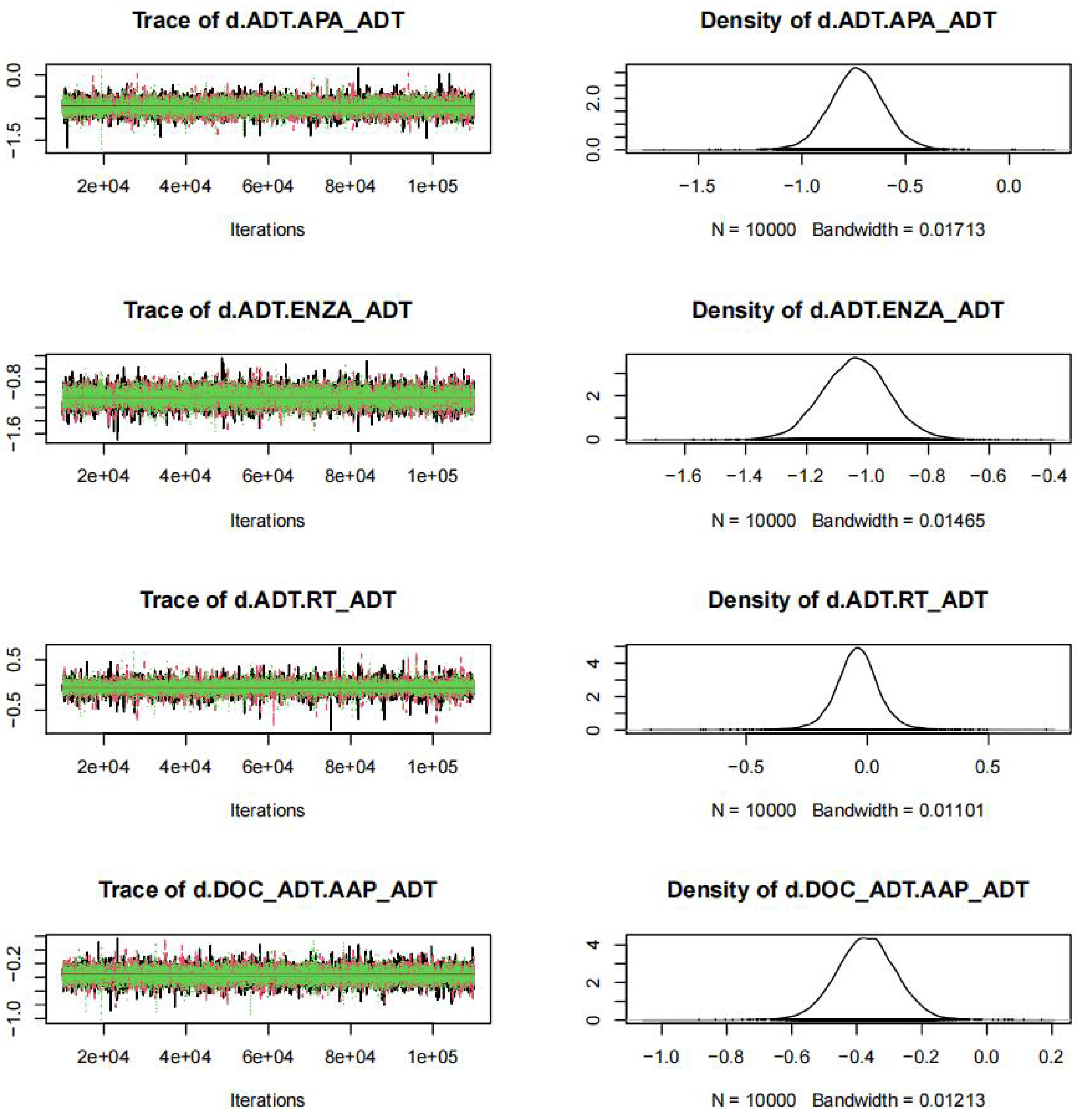

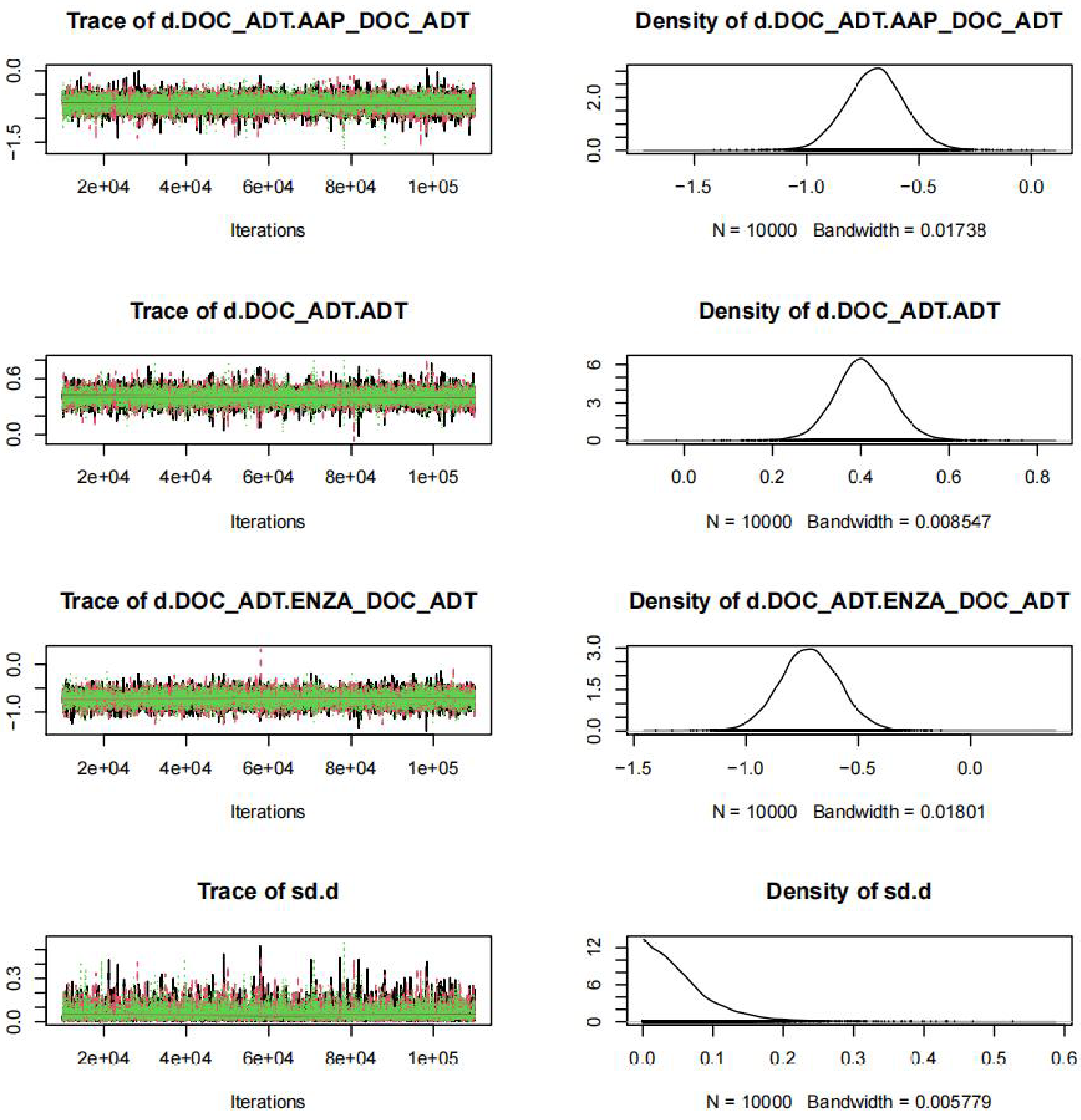
Trace of d.DOC_ADT.AAP_DOC_ADT
Iterations
Density of d.DOC_ADT.AAP_DOC_ADT
N = 10000 Bandwidth = 0.01738
Trace of d.DOC_ADT.ADT
Iterations
Density of d.DOC_ADT.ADT
N = 10000 Bandwidth = 0.008547
Trace of d.DOC_ADT.ENZA_DOC_ADT
Iterations
Density of d.DOC_ADT.ENZA_DOC_ADT
N = 10000 Bandwidth = 0.01801
Trace of sd.d
Iterations
Density of sd.d
N = 10000 Bandwidth = 0.005779

C: Trace and density plots for OS for high volume disease

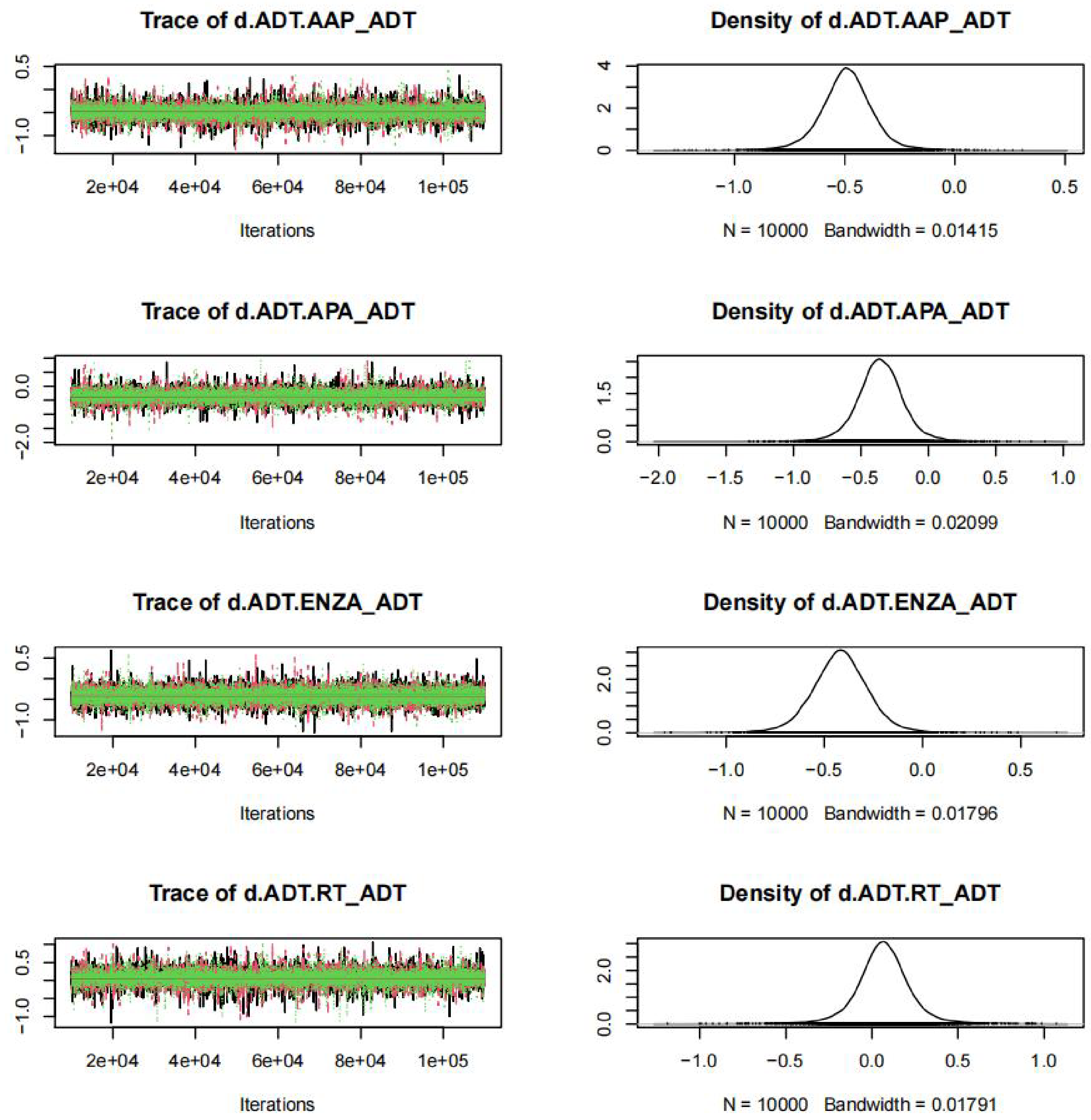

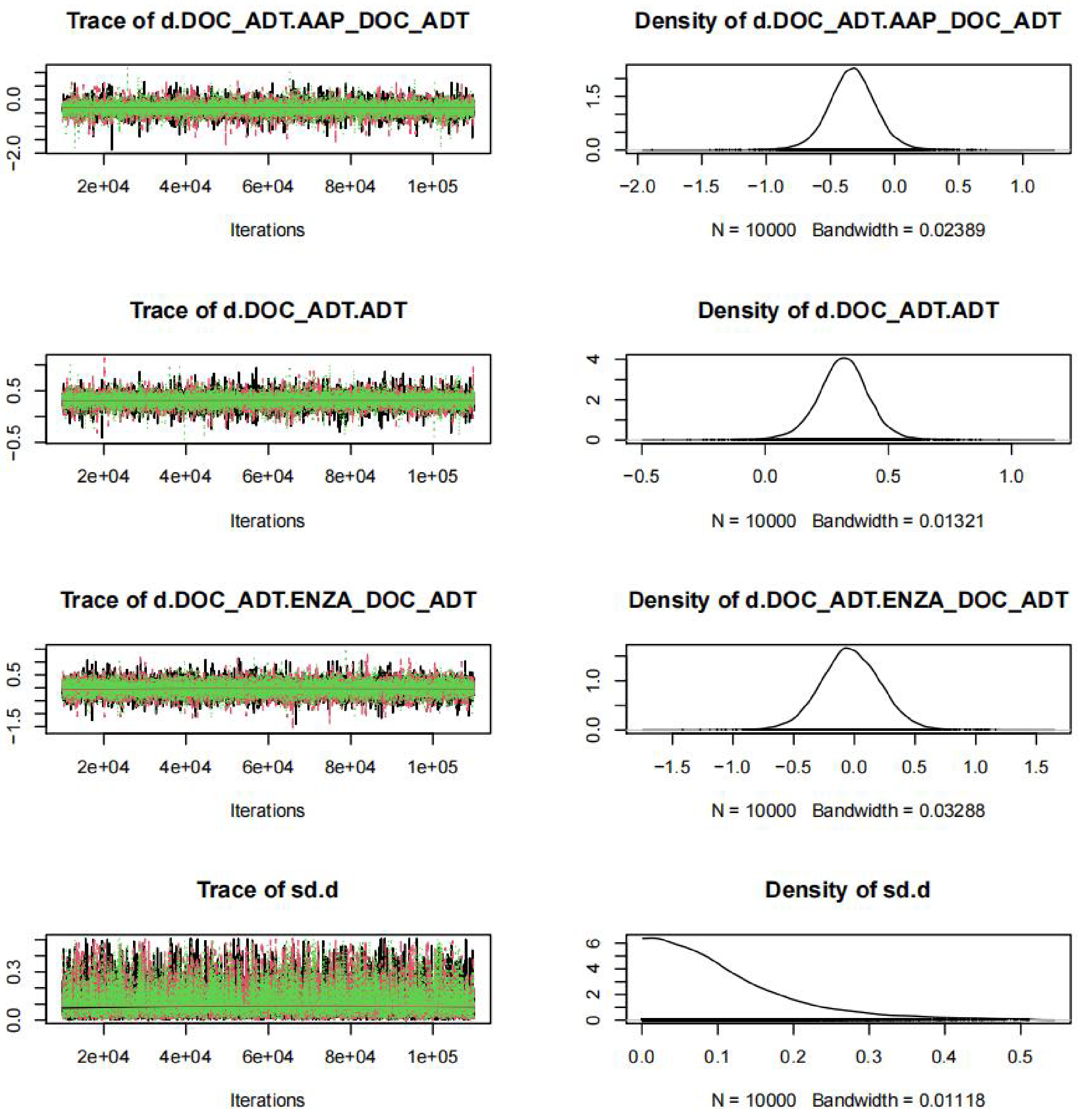
Trace of d.DOC_ADT.AAP_DOC_ADT
Iterations
Density of d.DOC_ADT.AAP_DOC_ADT
N = 10000 Bandwidth = 0.02389
Trace of d.DOC_ADT.ADT
Iterations
Density of d.DOC_ADT.ADT
N = 10000 Bandwidth = 0.01321
Trace of d.DOC_ADT.ENZA_DOC_ADT
Iterations
Density of d.DOC_ADT.ENZA_DOC_ADT
N = 10000 Bandwidth = 0.03288
Trace of sd.d
Iterations
Density of sd.d
N = 10000 Bandwidth = 0.01118

D: Trace and density plots for OS for low volume disease

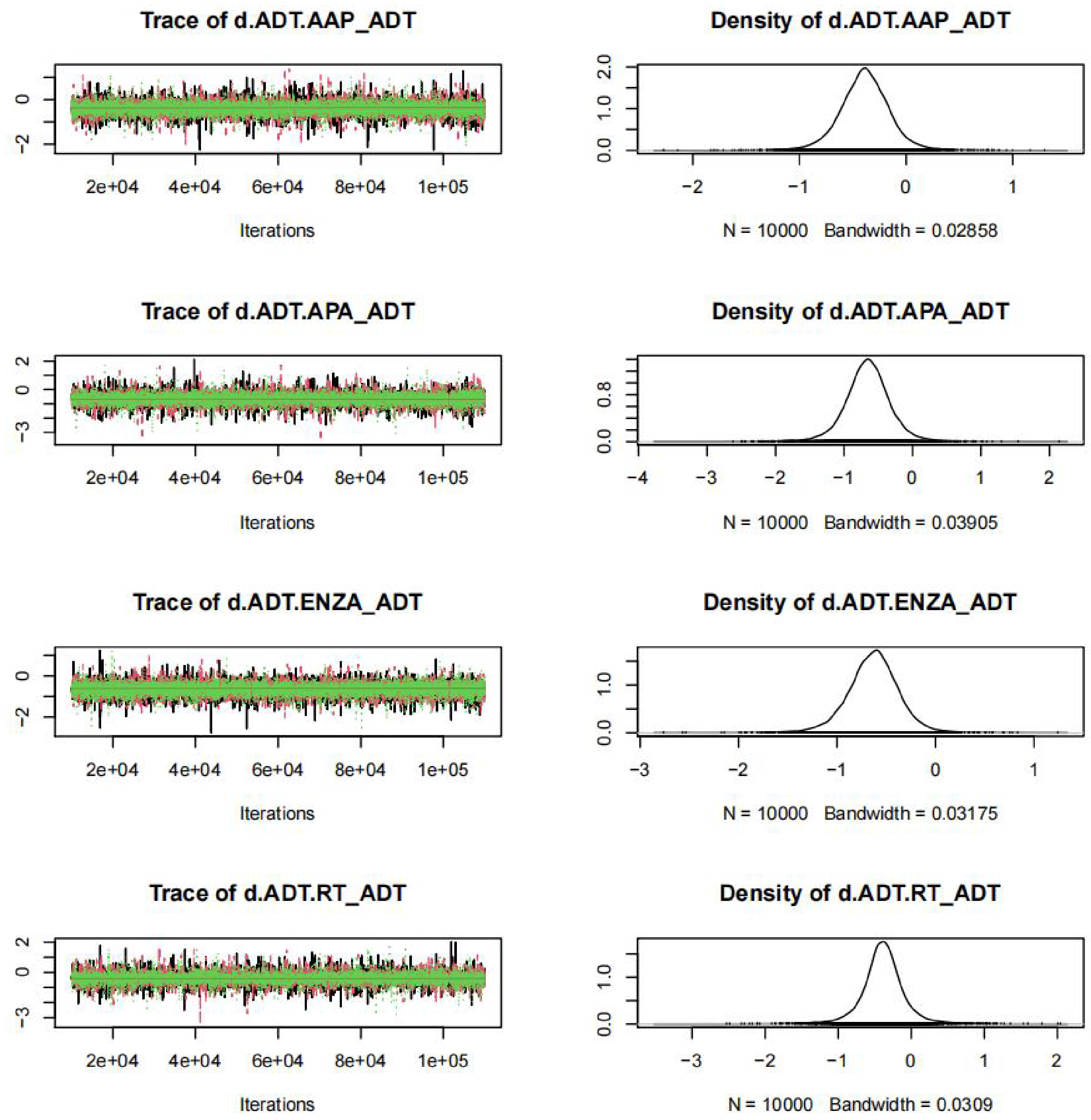

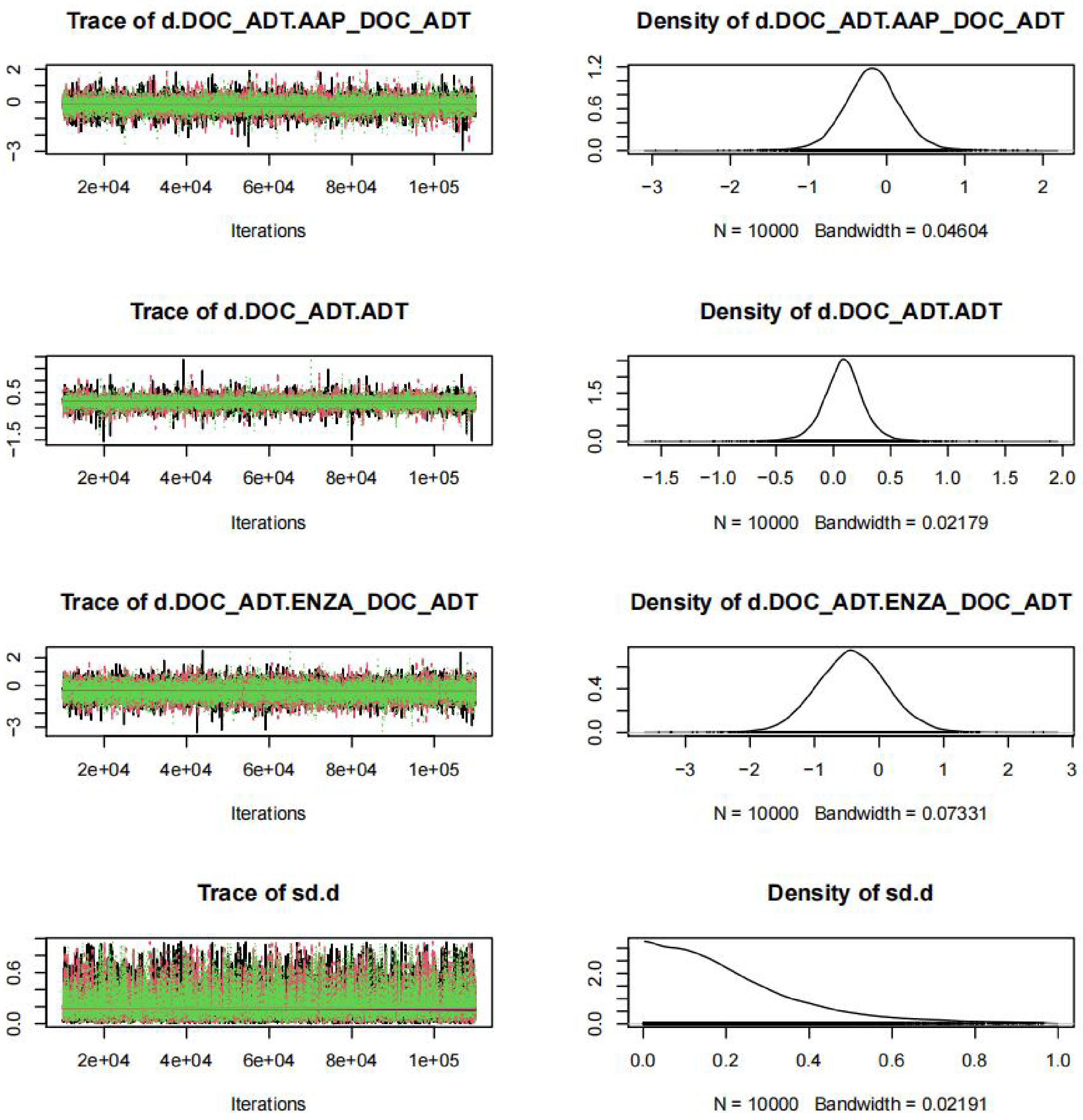
Trace of d.DOC_ADT.AAP_DOC_ADT
Density of d.DOC_ADT.AAP_DOC_ADT
Iterations
N = 10000 Bandwidth = 0.04604
Trace of d.DOC_ADT.ADT
Density of d.DOC_ADT.ADT
Iterations
N = 10000 Bandwidth = 0.02179
Trace of d.DOC_ADT.ENZA_DOC_ADT
Density of d.DOC_ADT.ENZA_DOC_ADT
Iterations
N = 10000 Bandwidth = 0.07331
Trace of sd.d
Density of sd.d
Iterations
N = 10000 Bandwidth = 0.02191

E: Trace and density plots for OS for visceral metastasis

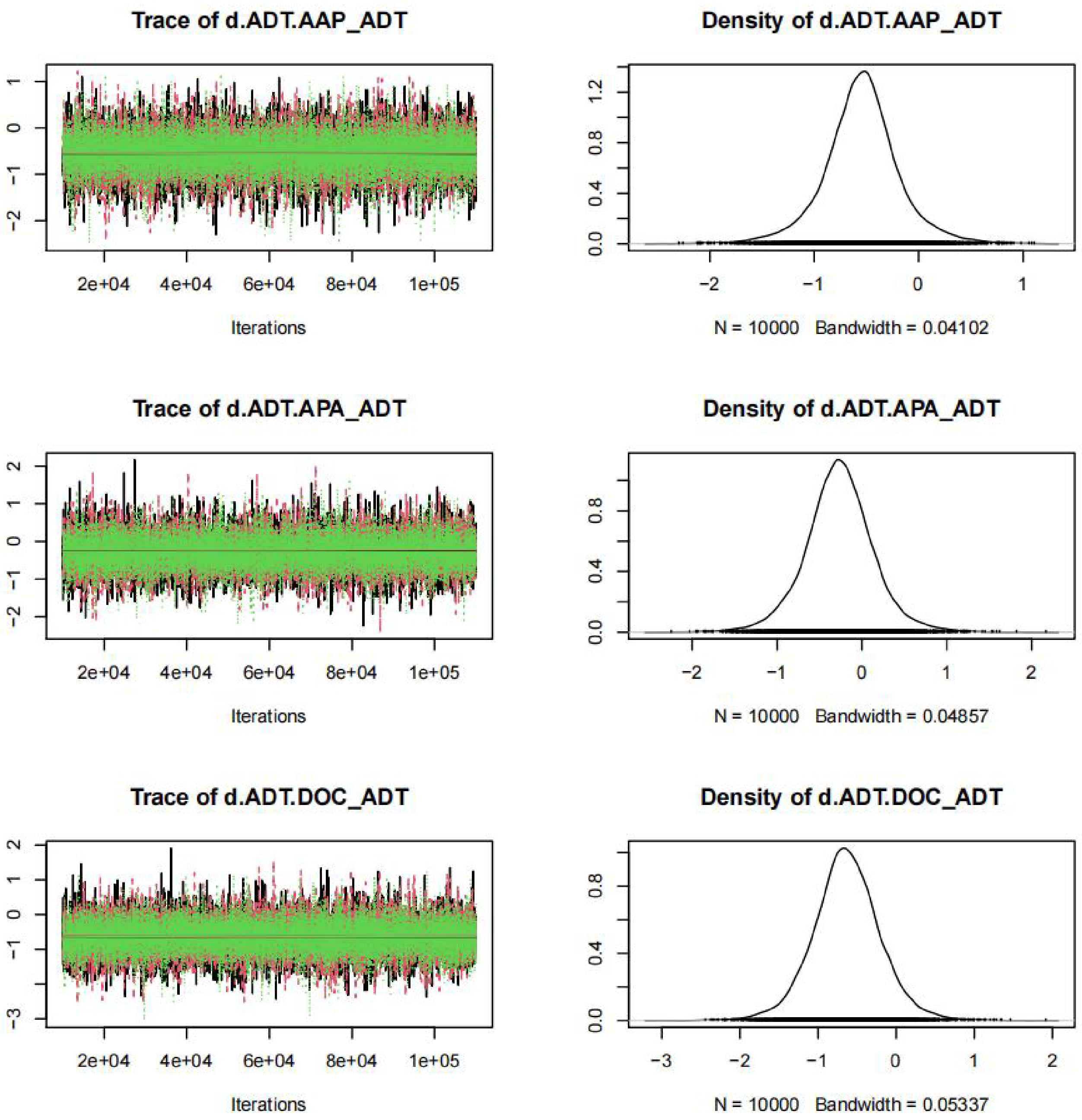

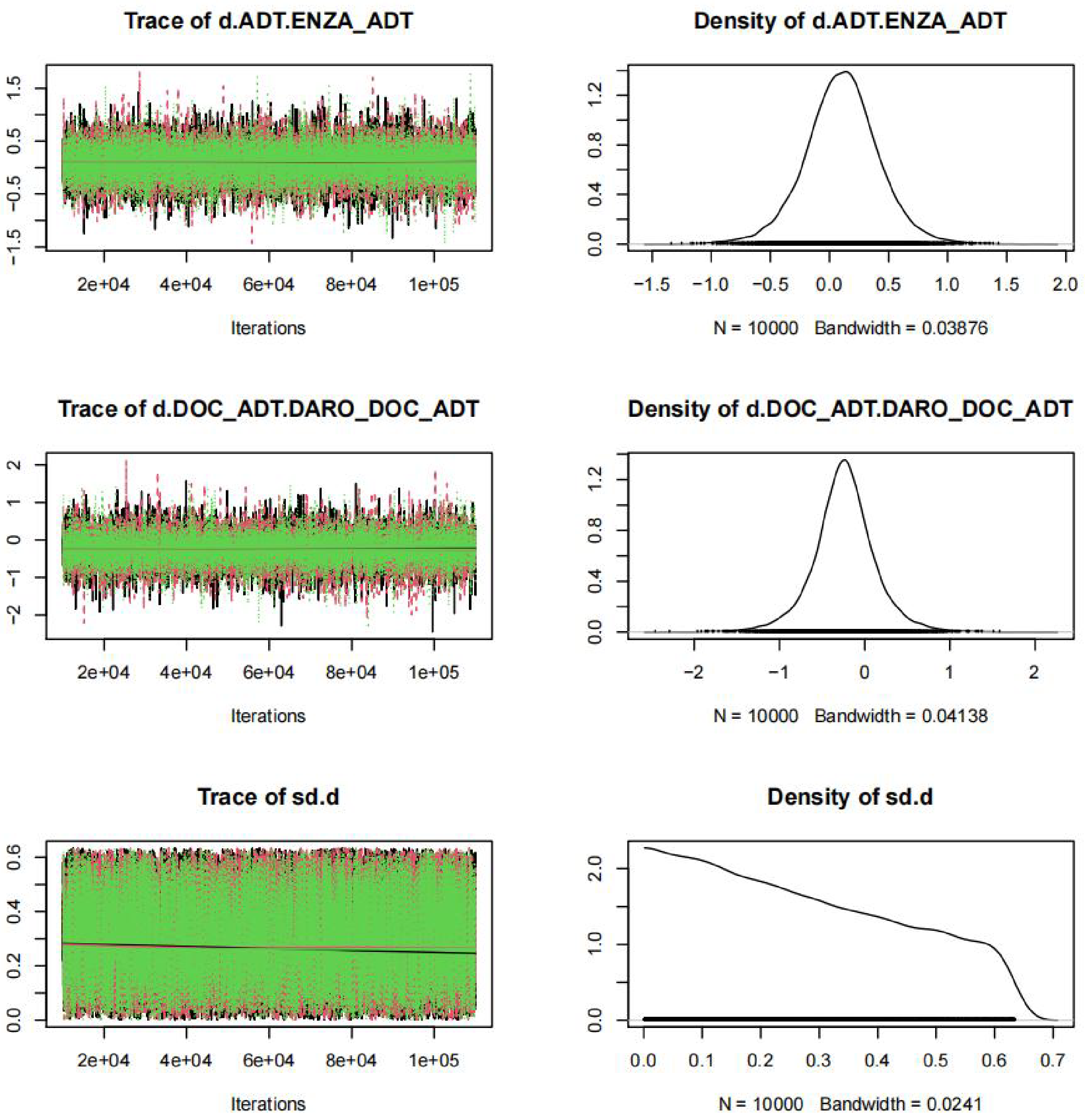
Trace of d.ADT.ENZA_ADT
Iterations
Density of d.ADT.ENZA_ADT
N = 10000 Bandwidth = 0.03876
Trace of d.DOC_ADT.DARO_DOC_ADT
Iterations
Density of d.DOC_ADT.DARO_DOC_ADT
N = 10000 Bandwidth = 0.04138
Trace of sd.d
Iterations
Density of sd.d
N = 10000 Bandwidth = 0.0241

F: Trace and density plots for high grade AE

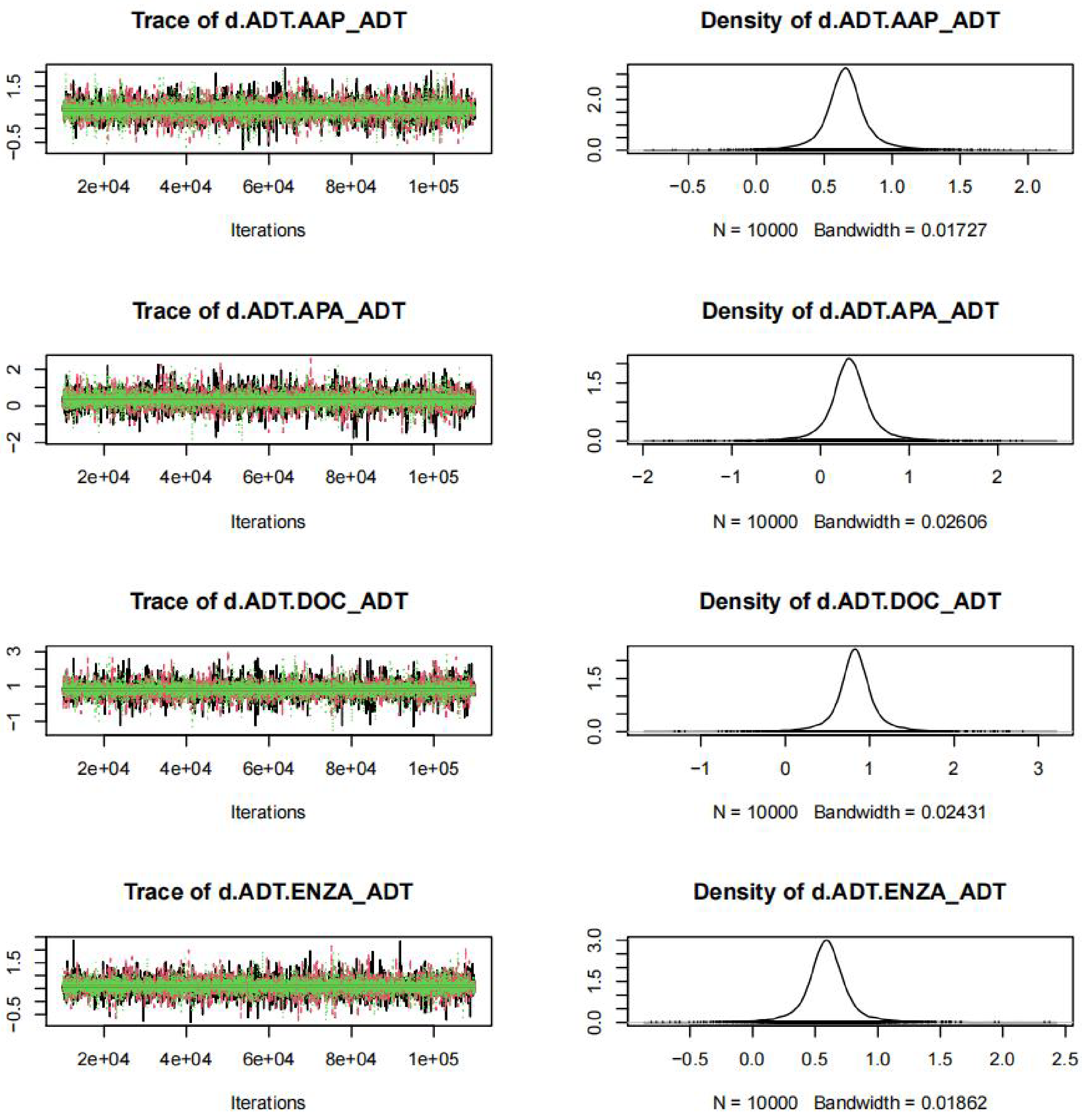

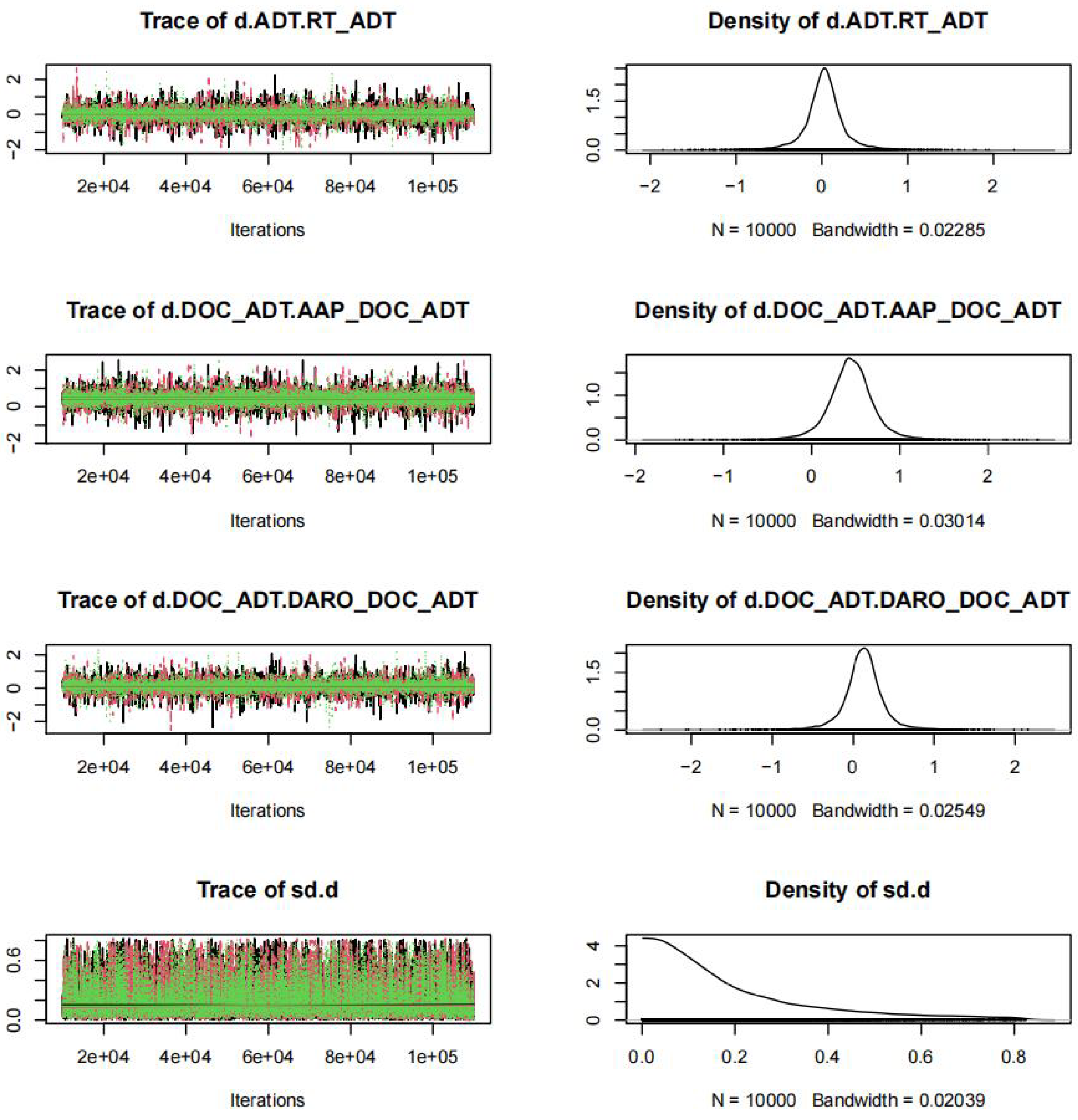
Trace of d.ADT.RT_ADT
Iterations
Density of d.ADT.RT_ADT
N = 10000   Bandwidth = 0.02285
Trace of d.DOC_ADT.AAP_DOC_ADT
Iterations
Density of d.DOC_ADT.AAP_DOC_ADT
N = 10000   Bandwidth = 0.03014
Trace of d.DOC_ADT.DARO_DOC_ADT
Iterations
Density of d.DOC_ADT.DARO_DOC_ADT
N = 10000   Bandwidth = 0.02549
Trace of sd.d
Iterations
Density of sd.d
N = 10000   Bandwidth = 0.02039

Supplementary Material 11: Summary of grade 3-5 adverse events in reported trials and corresponding odds ratio

STUDY No.	3	4	5	7	9	11	12	14	15
Trial	STAMPEDE-arm C	LATITUDE	TAMPEDE -arm G	ENZAMET	ARCHES	TITAN	STAMPEDE -RT	PEACE-1	ARASENS
G3-5 AE (Exp.)	288/550 (52%)	411/597 (69%)	443/948 (47%)	321/563 (57.0%)	254/572 (44.4%)	279/524 (53.2%)	380/985 (39%)	217/347 (62.5%)	458/652 (70.2%)
G3-5 AE (Control)	399/1228 (32%)	309/602 (51%)	315/960 (33%)	241/558 (43.2%)	172/574 (30.0%)	237/527 (45.0%)	398/1050 (38%)	181/350 (51.7%)	439/650 (67.5%)
OR	2.28 (1.86-2.81)	2.10 (1.66-2.65)	1.80 (1.49-2.16)	1.74 (1.38-2.21)	1.87 (1.46-2.38)	1.39 (1.09-1.78)	1.03 (0.86-1.23)	1.56 (1.15-2.11)	1.13 (0.90-1.44)
		1.86(1.61-2.14)		1.79(1.51-2.11)					

AE: adverse event; Exp.: experimental group; OR: odds ratio.

Supplementary Material 12: Assessment of the certainty of evidence using the confidence in network meta-analysis (CINeMA)

1. Efficacy-OS for all population

1.1 network plot drawn by CINeMA

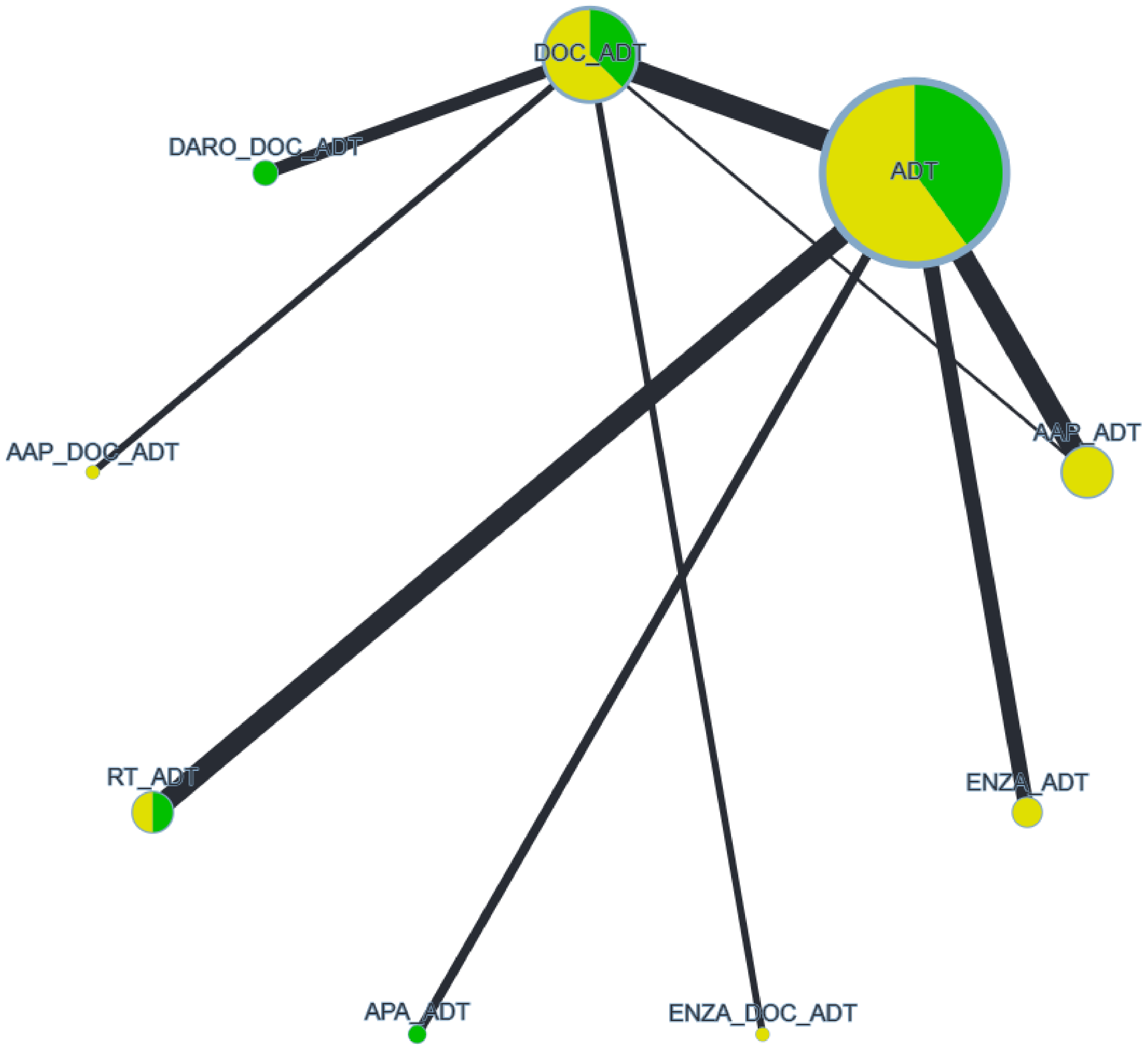

1.2 Risk of Bias Chart showing the contribution of low, moderate or high RoB comparisons to each network estimate

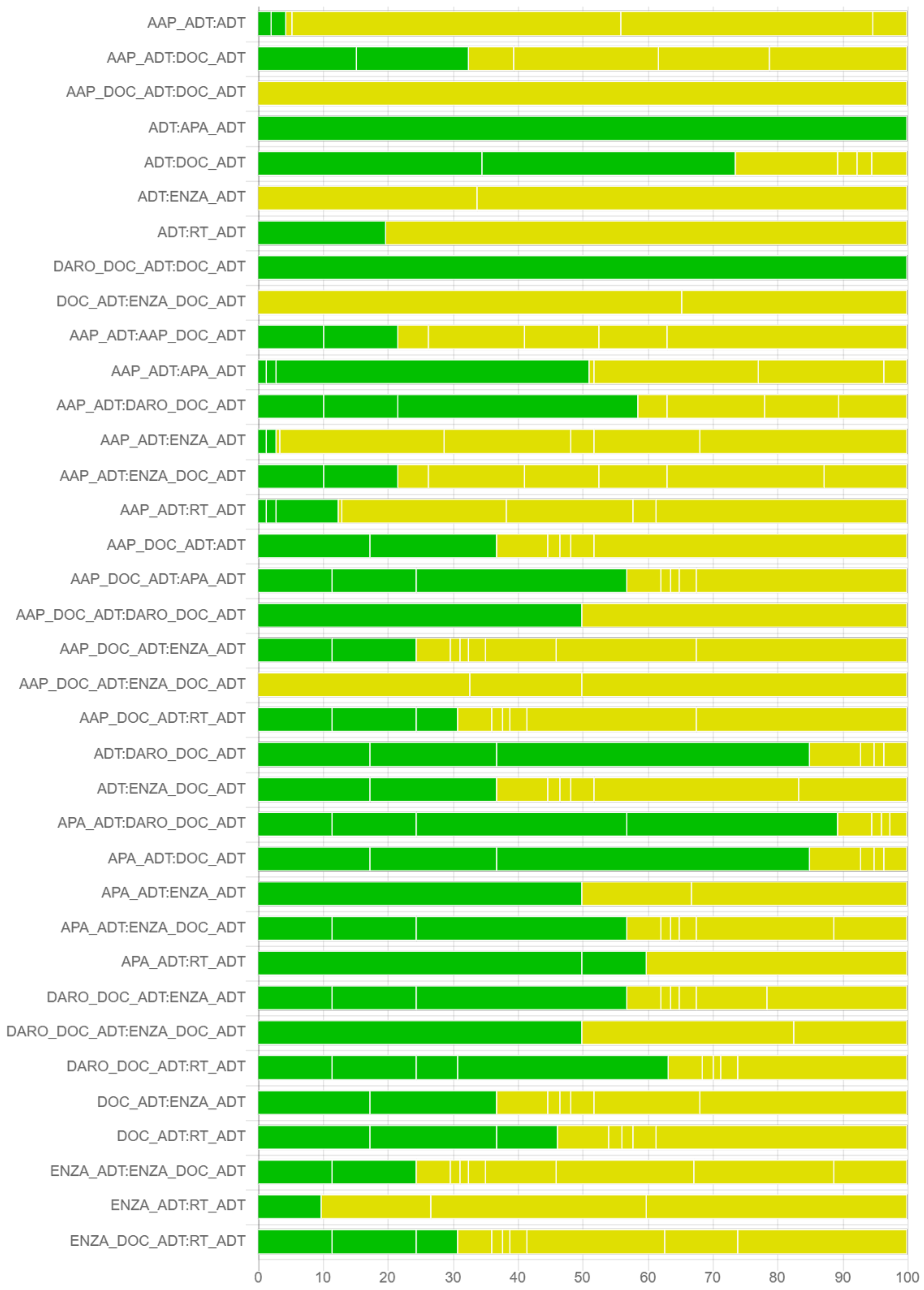

1.3 Evaluation of the Certainty of evidence Using CINEMA Framework, mixed evidence

Comparison	Number of studies	Within-study bias	Reporting bias	Indirectness	Imprecision	Heterogeneity	Incoherence	Confidence rating
AAP_ADT:ADT	2	Some concerns	Low risk	No concerns	No concerns	No concerns	Major concerns	Very low
AAP_ADT:DOC_ADT	1	Some concerns	Low risk	No concerns	Some concerns	No concerns	Major concerns	Very low
AAP_DOC_ADT:DOC_ADT	1	Some concerns	Low risk	No concerns	No concerns	Some concerns	Some concerns	Very low
ADT:APA_ADT	1	No concerns	Low risk	No concerns	No concerns	No concerns	Some concerns	Moderate
ADT:DOC_ADT	3	No concerns	Low risk	No concerns	No concerns	No concerns	Some concerns	Moderate
ADT:ENZA_ADT	2	Some concerns	Low risk	No concerns	No concerns	No concerns	Some concerns	Low
ADT:RT_ADT	2	Some concerns	Low risk	No concerns	Some concerns	No concerns	Some concerns	Low
DARO_DOC_ADT:DOC_ADT	1	No concerns	Low risk	No concerns	No concerns	No concerns	Some concerns	Moderate
DOC_ADT:ENZA_DOC_ADT	2	Some concerns	Low risk	No concerns	Some concerns	Some concerns	Some concerns	Very low
AAP_ADT:AAP_DOC_ADT	0	Some concerns	Low risk	No concerns	Some concerns	Some concerns	Some concerns	Very low
AAP_ADT:APA_ADT	0	No concerns	Low risk	No concerns	Some concerns	Some concerns	Some concerns	Very low
AAP_ADT:DARO_DOC_ADT	0	No concerns	Low risk	No concerns	No concerns	Some concerns	Some concerns	Low
AAP_ADT:ENZA_ADT	0	Some concerns	Low risk	No concerns	Major concerns	No concerns	Some concerns	Very low
AAP_ADT:ENZA_DOC_ADT	0	Some concerns	Low risk	No concerns	Major concerns	No concerns	Some concerns	Very low
AAP_ADT:RT_ADT	0	Some concerns	Low risk	No concerns	No concerns	No concerns	Some concerns	Low
AAP_DOC_ADT:ADT	0	Some concerns	Low risk	No concerns	No concerns	No concerns	Some concerns	Low
AAP_DOC_ADT:APA_ADT	0	No concerns	Low risk	No concerns	Major concerns	No concerns	Some concerns	High
AAP_DOC_ADT:DARO_DOC_ADT	0	Some concerns	Low risk	No concerns	Major concerns	No concerns	Some concerns	Very low
AAP_DOC_ADT:ENZA_ADT	0	Some concerns	Low risk	No concerns	Major concerns	No concerns	Some concerns	Very low
AAP_DOC_ADT:ENZA_DOC_ADT	0	Some concerns	Low risk	No concerns	Major concerns	No concerns	Some concerns	Very low
AAP_DOC_ADT:RT_ADT	0	Some concerns	Low risk	No concerns	No concerns	No concerns	Some concerns	Low
ADT:DARO_DOC_ADT	0	No concerns	Low risk	No concerns	No concerns	No concerns	Some concerns	Moderate
ADT:ENZA_DOC_ADT	0	Some concerns	Low risk	No concerns	No concerns	No concerns	Some concerns	Low
APA_ADT:DARO_DOC_ADT	0	No concerns	Low risk	No concerns	Some concerns	Some concerns	Some concerns	Very low
APA_ADT:DOC_ADT	0	No concerns	Low risk	No concerns	Some concerns	No concerns	Some concerns	Low
APA_ADT:ENZA_ADT	0	Some concerns	Low risk	No concerns	Major concerns	No concerns	Some concerns	Very low

APA_ADT:ENZA_DOC_ADT	0	No concerns	Low risk	No concerns	Major concerns	No concerns	Some concerns	Very low
APA_ADT:RT_ADT	0	No concerns	Low risk	No concerns	No concerns	No concerns	Some concerns	Moderate
DARO_DOC_ADT:ENZA_ADT	0	No concerns	Low risk	No concerns	Some concerns	No concerns	Some concerns	Low
DARO_DOC_ADT:ENZA_DOC_ADT	0	Some concerns	Low risk	No concerns	Some concerns	No concerns	Some concerns	Very low
DARO_DOC_ADT:RT_ADT	0	No concerns	Low risk	No concerns	No concerns	No concerns	Some concerns	Moderate
DOC_ADT:ENZA_ADT	0	Some concerns	Low risk	No concerns	Some concerns	No concerns	Some concerns	Very low
DOC_ADT:RT_ADT	0	Some concerns	Low risk	No concerns	Some concerns	No concerns	Some concerns	Very low
ENZA_ADT:ENZA_DOC_ADT	0	Some concerns	Low risk	No concerns	Major concerns	No concerns	Some concerns	Very low
ENZA_ADT:RT_ADT	0	Some concerns	Low risk	No concerns	No concerns	No concerns	Some concerns	Low
ENZA_DOC_ADT:RT_ADT	0	Some concerns	Low risk	No concerns	Some concerns	No concerns	Some concerns	Very low

2. Efficacy- generalized PFS for all population

2.1 network plot drawn by CINeMA

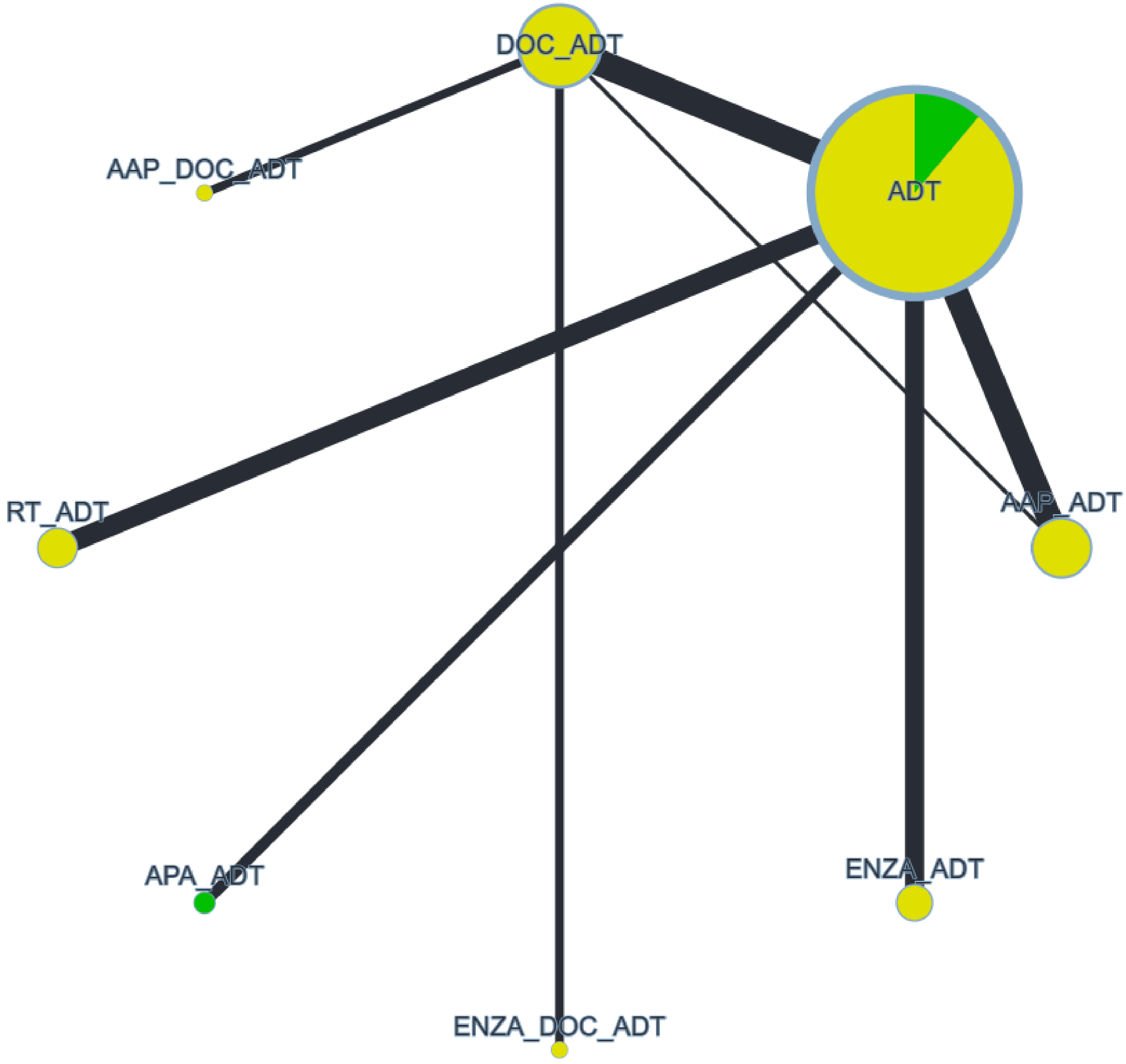

2.2 Risk of Bias Chart showing the contribution of low, moderate or high RoB comparisons to each network estimate

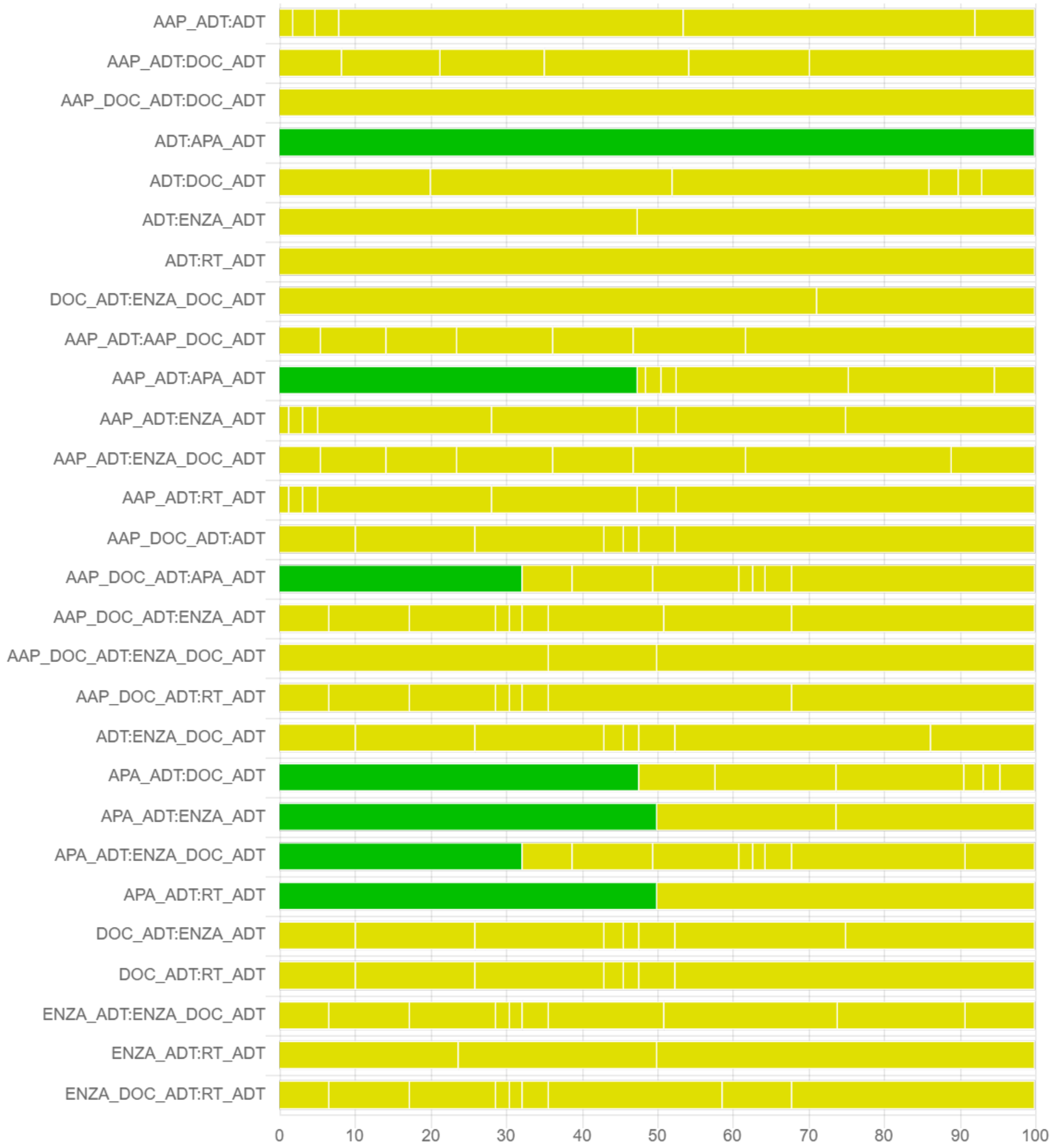

2.3 Evaluation of the Certainty of evidence Using CINEMA Framework, mixed evidence

Comparison	Number of studies	Within-study bias	Reporting bias	Indirectness	Imprecision	Heterogeneity	Incoherence	Confidence rating
AAP_ADT:ADT	2	Some concerns	Low risk	No concerns	No concerns	No concerns	No concerns	Moderate
AAP_ADT:DOC_ADT	1	Some concerns	Low risk	No concerns	No concerns	Some concerns	No concerns	Low
AAP_DOC_ADT:DOC_ADT	1	Some concerns	Low risk	No concerns	No concerns	No concerns	No concerns	Moderate
ADT:APA_ADT	1	No concerns	Low risk	No concerns	No concerns	No concerns	No concerns	High
ADT:DOC_ADT	3	Some concerns	Low risk	No concerns	No concerns	No concerns	No concerns	Moderate
ADT:ENZA_ADT	2	Some concerns	Low risk	No concerns	No concerns	No concerns	No concerns	Moderate
ADT:RT_ADT	1	Some concerns	Low risk	No concerns	Major concerns	No concerns	No concerns	Very low
DOC_ADT:ENZA_DOC_ADT	2	Some concerns	Low risk	No concerns	No concerns	No concerns	No concerns	Moderate
AAP_ADT:AAP_DOC_ADT	0	Some concerns	Low risk	No concerns	No concerns	Some concerns	No concerns	Low
AAP_ADT:APA_ADT	0	Some concerns	Low risk	No concerns	Major concerns	No concerns	No concerns	Very low
AAP_ADT:ENZA_ADT	0	Some concerns	Low risk	No concerns	No concerns	Some concerns	No concerns	Low
AAP_ADT:ENZA_DOC_ADT	0	Some concerns	Low risk	No concerns	No concerns	Some concerns	No concerns	Low
AAP_ADT:RT_ADT	0	Some concerns	Low risk	No concerns	No concerns	No concerns	No concerns	Moderate
AAP_DOC_ADT:ADT	0	Some concerns	Low risk	No concerns	No concerns	No concerns	No concerns	Moderate
AAP_DOC_ADT:APA_ADT	0	Some concerns	Low risk	No concerns	Some concerns	Some concerns	No concerns	Very low
AAP_DOC_ADT:ENZA_ADT	0	Some concerns	Low risk	No concerns	Major concerns	No concerns	No concerns	Very low
AAP_DOC_ADT:ENZA_DOC_ADT	0	Some concerns	Low risk	No concerns	Major concerns	No concerns	No concerns	Very low
AAP_DOC_ADT:RT_ADT	0	Some concerns	Low risk	No concerns	No concerns	No concerns	No concerns	Moderate
ADT:ENZA_DOC_ADT	0	Some concerns	Low risk	No concerns	No concerns	No concerns	No concerns	Moderate
APA_ADT:DOC_ADT	0	Some concerns	Low risk	No concerns	Some concerns	Some concerns	No concerns	Very low
APA_ADT:ENZA_ADT	0	Some concerns	Low risk	No concerns	Some concerns	Some concerns	No concerns	Very low
APA_ADT:ENZA_DOC_ADT	0	Some concerns	Low risk	No concerns	Some concerns	Some concerns	No concerns	Very low
APA_ADT:RT_ADT	0	Some concerns	Low risk	No concerns	No concerns	No concerns	No concerns	Moderate
DOC_ADT:ENZA_ADT	0	Some concerns	Low risk	No concerns	No concerns	No concerns	No concerns	Moderate
DOC_ADT:RT_ADT	0	Some concerns	Low risk	No concerns	No concerns	Some concerns	No concerns	Low
ENZA_ADT:ENZA_DOC_ADT	0	Some concerns	Low risk	No concerns	Major concerns	No concerns	No concerns	Very low
ENZA_ADT:RT_ADT	0	Some concerns	Low risk	No concerns	No concerns	No concerns	No concerns	Moderate
ENZA_DOC_ADT:RT_ADT	0	Some concerns	Low risk	No concerns	No concerns	No concerns	No concerns	Moderate

3. Efficacy-OS for HVD

3.1 network plot drawn by CINeMA

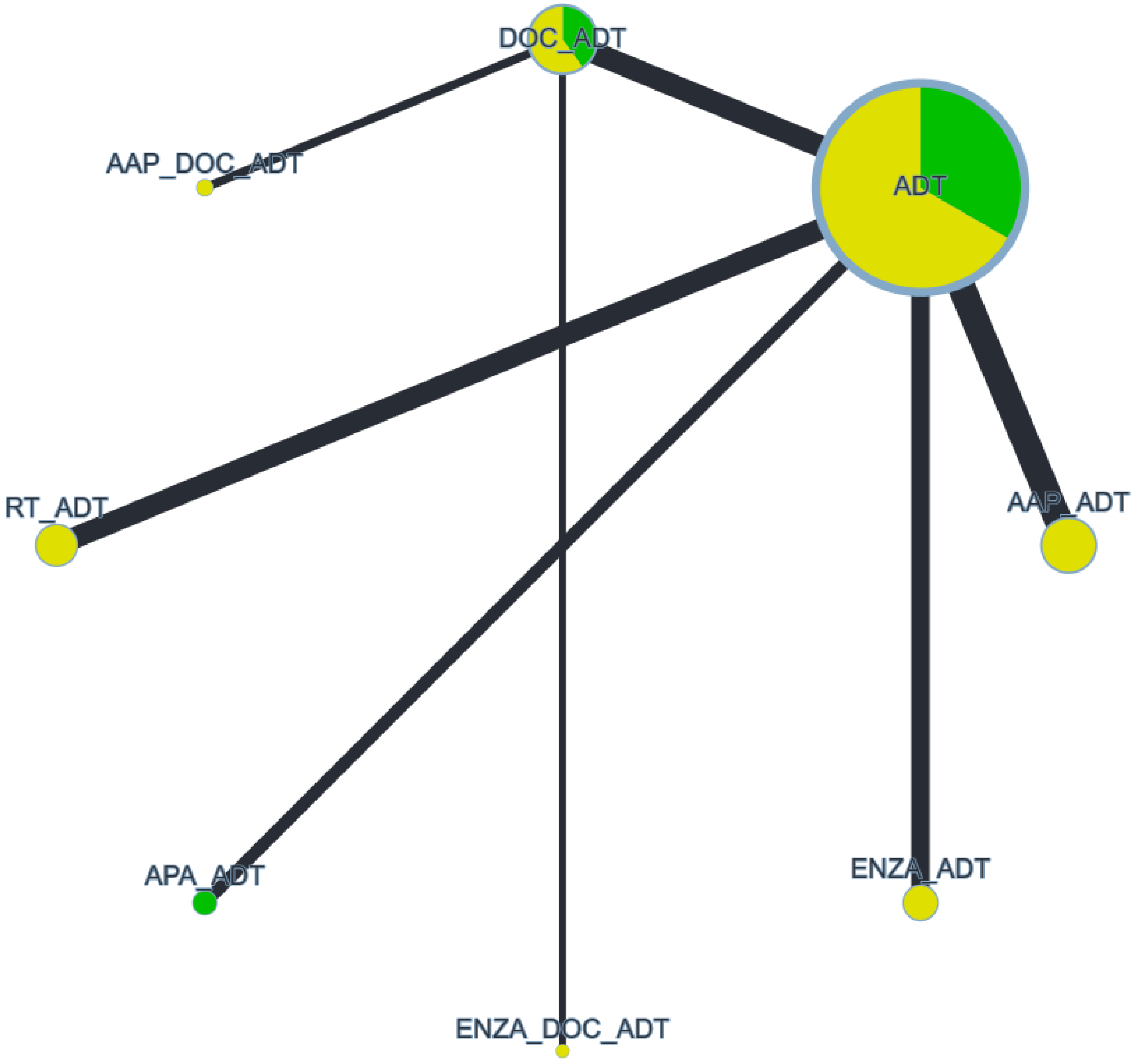

3.2 Risk of Bias Chart showing the contribution of low, moderate or high RoB comparisons to each network estimate

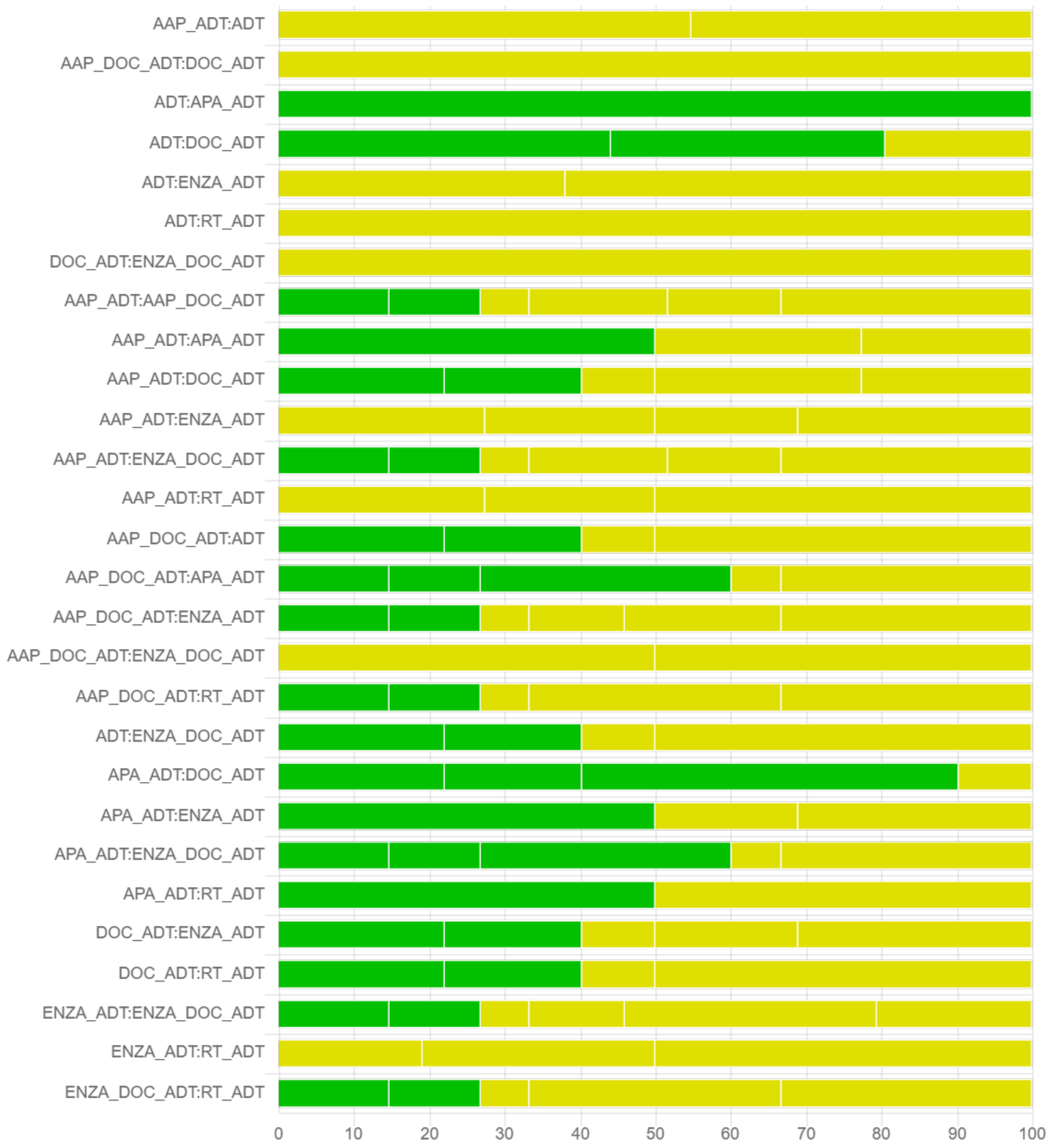

3.3 Evaluation of the Certainty of evidence Using CINEMA Framework, mixed evidence

Comparison	Number of studies	Within-study bias	Reporting bias	Indirectness	Imprecision	Heterogeneity	Incoherence	Confidence rating
AAP_ADT:ADT	2	Some concerns	Low risk	No concerns	No concerns	No concerns	Major concerns	Very low
AAP_DOC_ADT:DOC_ADT	1	Some concerns	Low risk	No concerns	No concerns	Some concerns	Major concerns	Very low
ADT:APA_ADT	1	No concerns	Low risk	No concerns	No concerns	Some concerns	Major concerns	Very low
ADT:DOC_ADT	3	No concerns	Low risk	No concerns	No concerns	No concerns	Major concerns	Low
ADT:ENZA_ADT	2	Some concerns	Low risk	No concerns	No concerns	Some concerns	Major concerns	Very low
ADT:RT_ADT	1	Some concerns	Low risk	No concerns	Some concerns	Some concerns	Major concerns	Very low
DOC_ADT:ENZA_DOC_ADT	1	Some concerns	Low risk	No concerns	Major concerns	No concerns	Major concerns	Very low
AAP_ADT:AAP_DOC_ADT	0	Some concerns	Low risk	No concerns	Some concerns	Some concerns	Major concerns	Very low
AAP_ADT:APA_ADT	0	Some concerns	Low risk	No concerns	Major concerns	No concerns	Major concerns	Very low
AAP_ADT:DOC_ADT	0	Some concerns	Low risk	No concerns	Major concerns	No concerns	Major concerns	Very low
AAP_ADT:ENZA_ADT	0	Some concerns	Low risk	No concerns	Some concerns	Some concerns	Major concerns	Very low
AAP_ADT:ENZA_DOC_ADT	0	Some concerns	Low risk	No concerns	Major concerns	No concerns	Major concerns	Very low
AAP_ADT:RT_ADT	0	Some concerns	Low risk	No concerns	No concerns	No concerns	Major concerns	Very low
AAP_DOC_ADT:ADT	0	Some concerns	Low risk	No concerns	No concerns	No concerns	Major concerns	Very low
AAP_DOC_ADT:APA_ADT	0	No concerns	Low risk	No concerns	Some concerns	Some concerns	Major concerns	Very low
AAP_DOC_ADT:ENZA_ADT	0	Some concerns	Low risk	No concerns	No concerns	Major concerns	Major concerns	Very low
AAP_DOC_ADT:ENZA_DOC_ADT	0	Some concerns	Low risk	No concerns	Some concerns	Some concerns	Major concerns	Very low
AAP_DOC_ADT:RT_ADT	0	Some concerns	Low risk	No concerns	No concerns	No concerns	Major concerns	Very low
ADT:ENZA_DOC_ADT	0	Some concerns	Low risk	No concerns	Some concerns	Some concerns	Major concerns	Very low
APA_ADT:DOC_ADT	0	No concerns	Low risk	No concerns	Major concerns	No concerns	Major concerns	Very low
APA_ADT:ENZA_ADT	0	Some concerns	Low risk	No concerns	Major concerns	No concerns	Major concerns	Very low
APA_ADT:ENZA_DOC_ADT	0	No concerns	Low risk	No concerns	Major concerns	No concerns	Major concerns	Very low
APA_ADT:RT_ADT	0	Some concerns	Low risk	No concerns	No concerns	Some concerns	Major concerns	Very low
DOC_ADT:ENZA_ADT	0	Some concerns	Low risk	No concerns	Major concerns	No concerns	Major concerns	Very low
DOC_ADT:RT_ADT	0	Some concerns	Low risk	No concerns	No concerns	No concerns	Major concerns	Very low
ENZA_ADT:ENZA_DOC_ADT	0	Some concerns	Low risk	No concerns	Major concerns	No concerns	Major concerns	Very low
ENZA_ADT:RT_ADT	0	Some concerns	Low risk	No concerns	No concerns	Some concerns	Major concerns	Very low
ENZA_DOC_ADT:RT_ADT	0	Some concerns	Low risk	No concerns	Some concerns	Some concerns	Major concerns	Very low

4. Efficacy-OS for LVD

4.1 network plot drawn by CINeMA

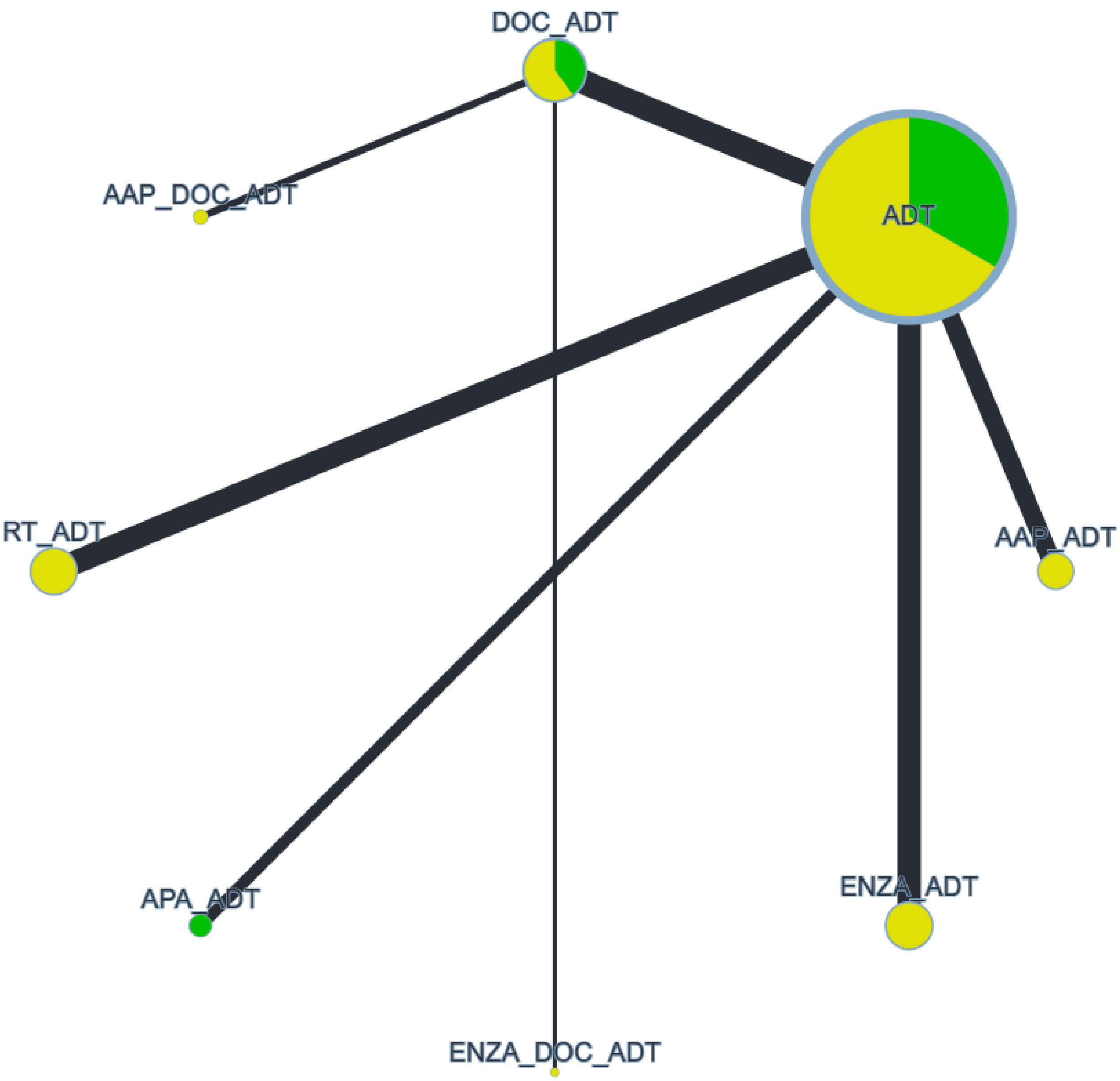

4.2 Risk of Bias Chart showing the contribution of low, moderate or high RoB comparisons to each network estimate

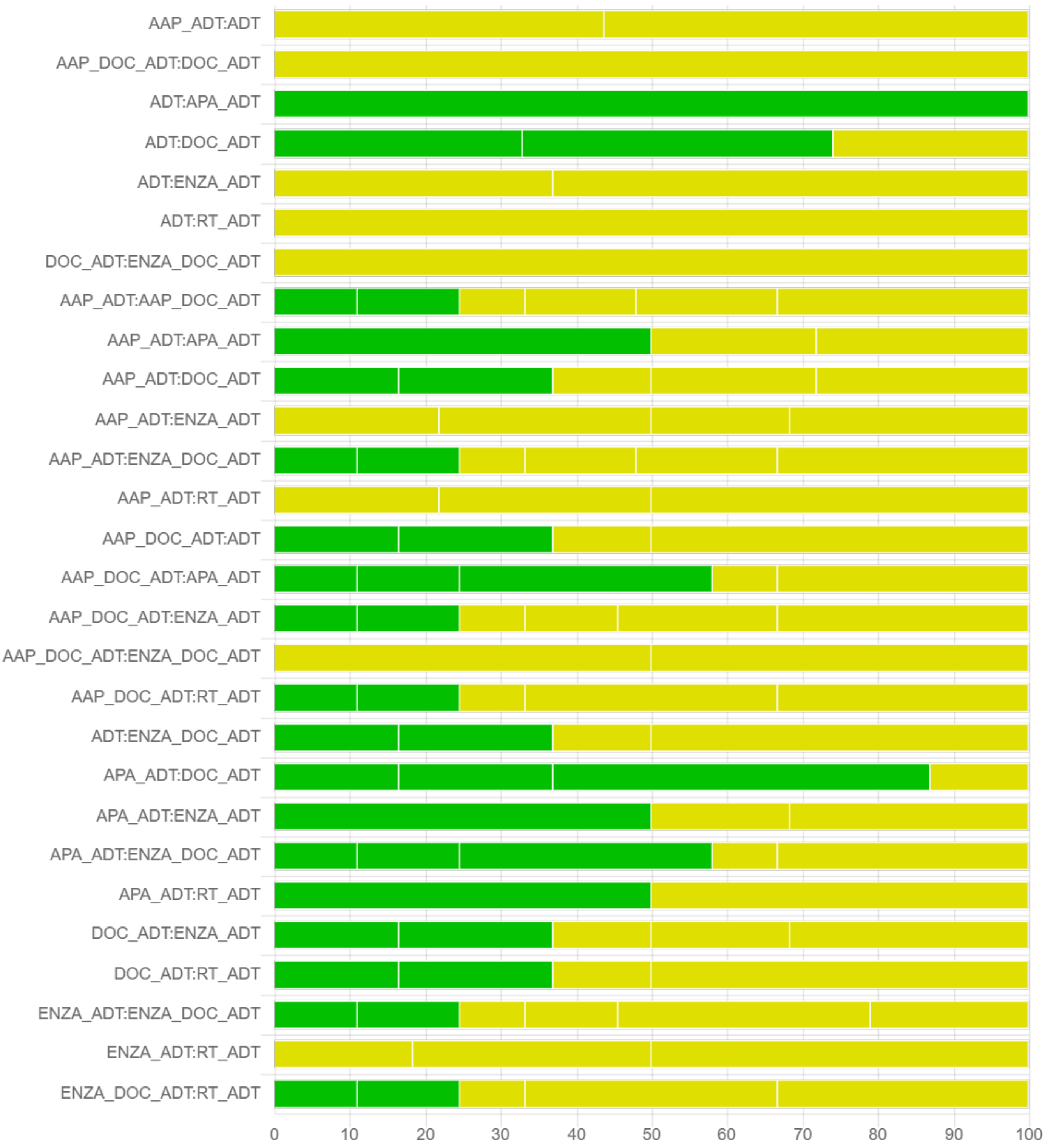

4.3 Evaluation of the Certainty of evidence Using CINEMA Framework, mixed evidence

Comparison	Number of studies	Within-study bias	Reporting bias	Indirectness	Imprecision	Heterogeneity	Incoherence	Confidence rating
AAP_ADT:ADT	2	Some concerns	Low risk	No concerns	No concerns	Some concerns	Major concerns	Very low
AAP_DOC_ADT:DOC_ADT	1	Some concerns	Low risk	No concerns	Major concerns	No concerns	Major concerns	Very low
ADT:APA_ADT	1	No concerns	Low risk	No concerns	Some concerns	Some concerns	Major concerns	Very low
ADT:DOC_ADT	3	No concerns	Low risk	No concerns	Some concerns	Some concerns	Major concerns	Very low
ADT:ENZA_ADT	2	Some concerns	Low risk	No concerns	No concerns	No concerns	Major concerns	Very low
ADT:RT_ADT	1	Some concerns	Low risk	No concerns	No concerns	Some concerns	Major concerns	Very low
DOC_ADT:ENZA_DOC_ADT	1	Some concerns	Low risk	No concerns	Major concerns	No concerns	Major concerns	Very low
AAP_ADT:AAP_DOC_ADT	0	Some concerns	Low risk	No concerns	Major concerns	No concerns	Major concerns	Very low
AAP_ADT:APA_ADT	0	Some concerns	Low risk	No concerns	Major concerns	No concerns	Major concerns	Very low
AAP_ADT:DOC_ADT	0	Some concerns	Low risk	No concerns	Some concerns	Some concerns	Major concerns	Very low
AAP_ADT:ENZA_ADT	0	Some concerns	Low risk	No concerns	Major concerns	No concerns	Major concerns	Very low
AAP_ADT:ENZA_DOC_ADT	0	Some concerns	Low risk	No concerns	Major concerns	No concerns	Major concerns	Very low
AAP_ADT:RT_ADT	0	Some concerns	Low risk	No concerns	Major concerns	No concerns	Major concerns	Very low
AAP_DOC_ADT:ADT	0	Some concerns	Low risk	No concerns	Major concerns	No concerns	Major concerns	Very low
AAP_DOC_ADT:APA_ADT	0	No concerns	Low risk	No concerns	Major concerns	No concerns	Major concerns	Very low
AAP_DOC_ADT:ENZA_ADT	0	Some concerns	Low risk	No concerns	Major concerns	No concerns	Major concerns	Very low
AAP_DOC_ADT:ENZA_DOC_ADT	0	Some concerns	Low risk	No concerns	Major concerns	No concerns	Major concerns	Very low
AAP_DOC_ADT:RT_ADT	0	Some concerns	Low risk	No concerns	Major concerns	No concerns	Major concerns	Very low
ADT:ENZA_DOC_ADT	0	Some concerns	Low risk	No concerns	Major concerns	No concerns	Major concerns	Very low
APA_ADT:DOC_ADT	0	No concerns	Low risk	No concerns	Major concerns	No concerns	Major concerns	Very low
APA_ADT:ENZA_ADT	0	Some concerns	Low risk	No concerns	Major concerns	No concerns	Major concerns	Very low
APA_ADT:ENZA_DOC_ADT	0	No concerns	Low risk	No concerns	Major concerns	No concerns	Major concerns	Very low
APA_ADT:RT_ADT	0	Some concerns	Low risk	No concerns	Major concerns	No concerns	Major concerns	Very low
DOC_ADT:ENZA_ADT	0	Some concerns	Low risk	No concerns	No concerns	Some concerns	Major concerns	Very low
DOC_ADT:RT_ADT	0	Some concerns	Low risk	No concerns	Some concerns	Some concerns	Major concerns	Very low
ENZA_ADT:ENZA_DOC_ADT	0	Some concerns	Low risk	No concerns	Major concerns	No concerns	Major concerns	Very low
ENZA_ADT:RT_ADT	0	Some concerns	Low risk	No concerns	Some concerns	Some concerns	Major concerns	Very low
ENZA_DOC_ADT:RT_ADT	0	Some concerns	Low risk	No concerns	Major concerns	No concerns	Major concerns	Very low

5. Efficacy-OS for visceral metastasis

5.1 network plot drawn by CINeMA

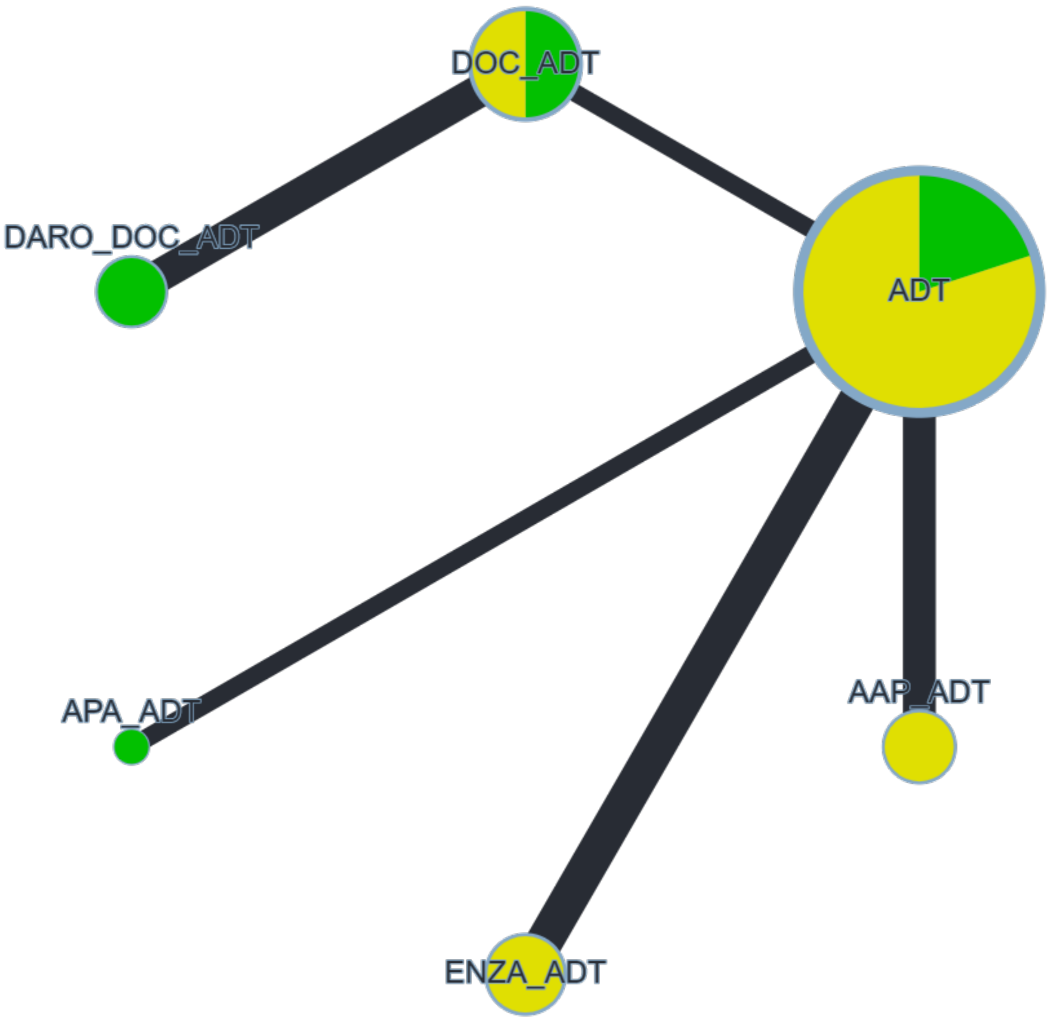

5.2 Risk of Bias Chart showing the contribution of low, moderate or high RoB comparisons to each network estimate

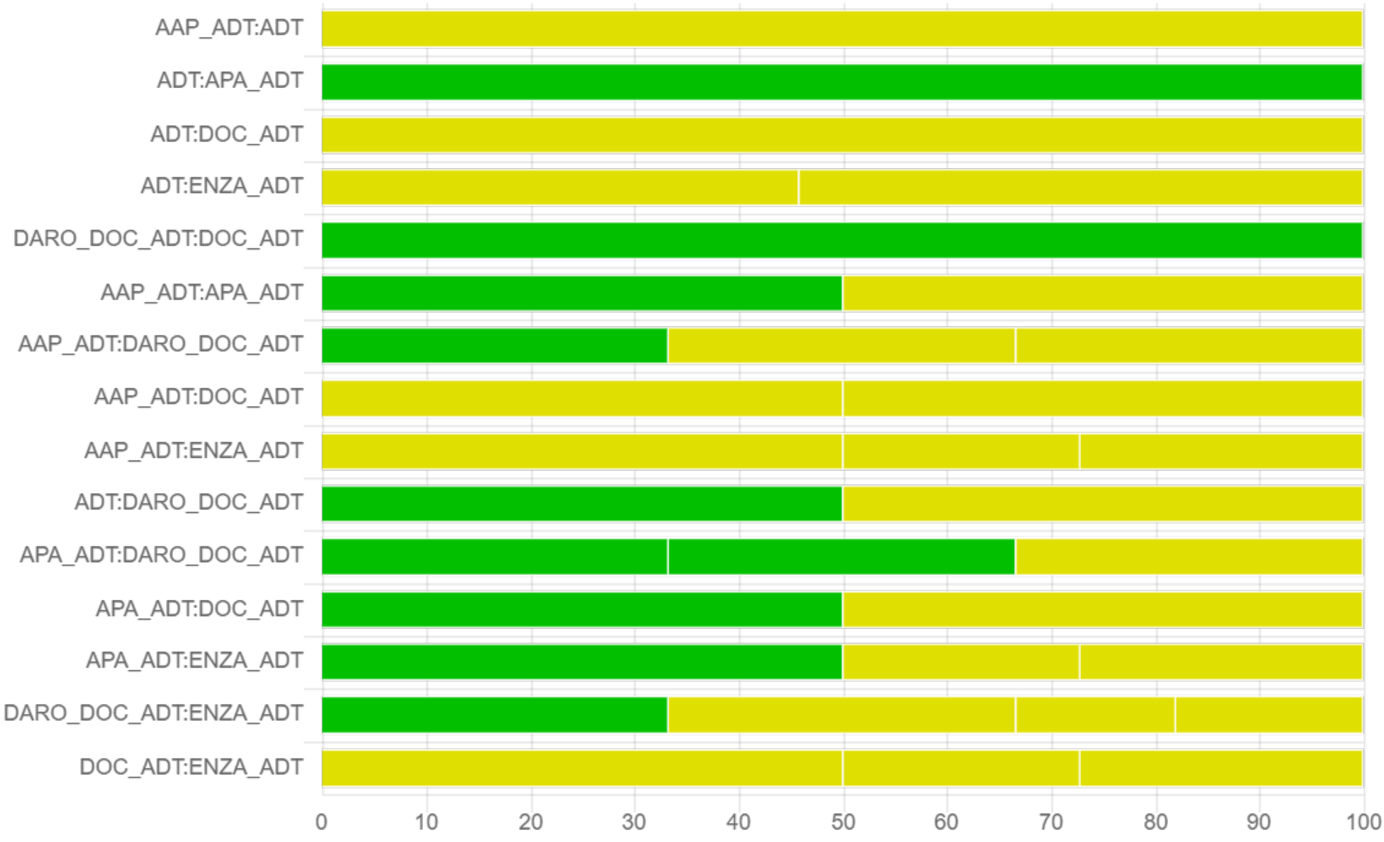

5.3 Evaluation of the Certainty of evidence Using CINEMA Framework, mixed evidence

Comparison	Number of studies	Within-study bias	Reporting bias	Indirectness	Imprecision	Heterogeneity	Incoherence	Confidence rating
AAP_ADT:ADT	1	Some concerns	Low risk	No concerns	No concerns	Major concerns	Major concerns	Very low
ADT:APA_ADT	1	No concerns	Low risk	No concerns	Major concerns	No concerns	Major concerns	Very low
ADT:DOC_ADT	1	Some concerns	Low risk	No concerns	Some concerns	Some concerns	Major concerns	Very low
ADT:ENZA_ADT	2	Some concerns	Low risk	No concerns	Major concerns	No concerns	Major concerns	Very low
DARO_DOC_ADT:DOC_ADT	1	No concerns	Low risk	No concerns	Major concerns	No concerns	Major concerns	Very low
AAP_ADT:APA_ADT	0	Some concerns	Low risk	No concerns	Major concerns	No concerns	Major concerns	Very low
AAP_ADT:DARO_DOC_ADT	0	Some concerns	Low risk	No concerns	Major concerns	No concerns	Major concerns	Very low
AAP_ADT:DOC_ADT	0	Some concerns	Low risk	No concerns	Major concerns	No concerns	Major concerns	Very low
AAP_ADT:ENZA_ADT	0	Some concerns	Low risk	No concerns	No concerns	Major concerns	Major concerns	Very low
ADT:DARO_DOC_ADT	0	Some concerns	Low risk	No concerns	Some concerns	Some concerns	Major concerns	Very low
APA_ADT:DARO_DOC_ADT	0	No concerns	Low risk	No concerns	Major concerns	No concerns	Major concerns	Very low
APA_ADT:DOC_ADT	0	Some concerns	Low risk	No concerns	Major concerns	No concerns	Major concerns	Very low
APA_ADT:ENZA_ADT	0	Some concerns	Low risk	No concerns	Some concerns	Some concerns	Major concerns	Very low
DARO_DOC_ADT:ENZA_ADT	0	Some concerns	Low risk	No concerns	No concerns	Major concerns	Major concerns	Very low
DOC_ADT:ENZA_ADT	0	Some concerns	Low risk	No concerns	Some concerns	Some concerns	Major concerns	Very low

6.Safety-Grade 3-5 adverse envents

6.1 network plot drawn by CINeMA

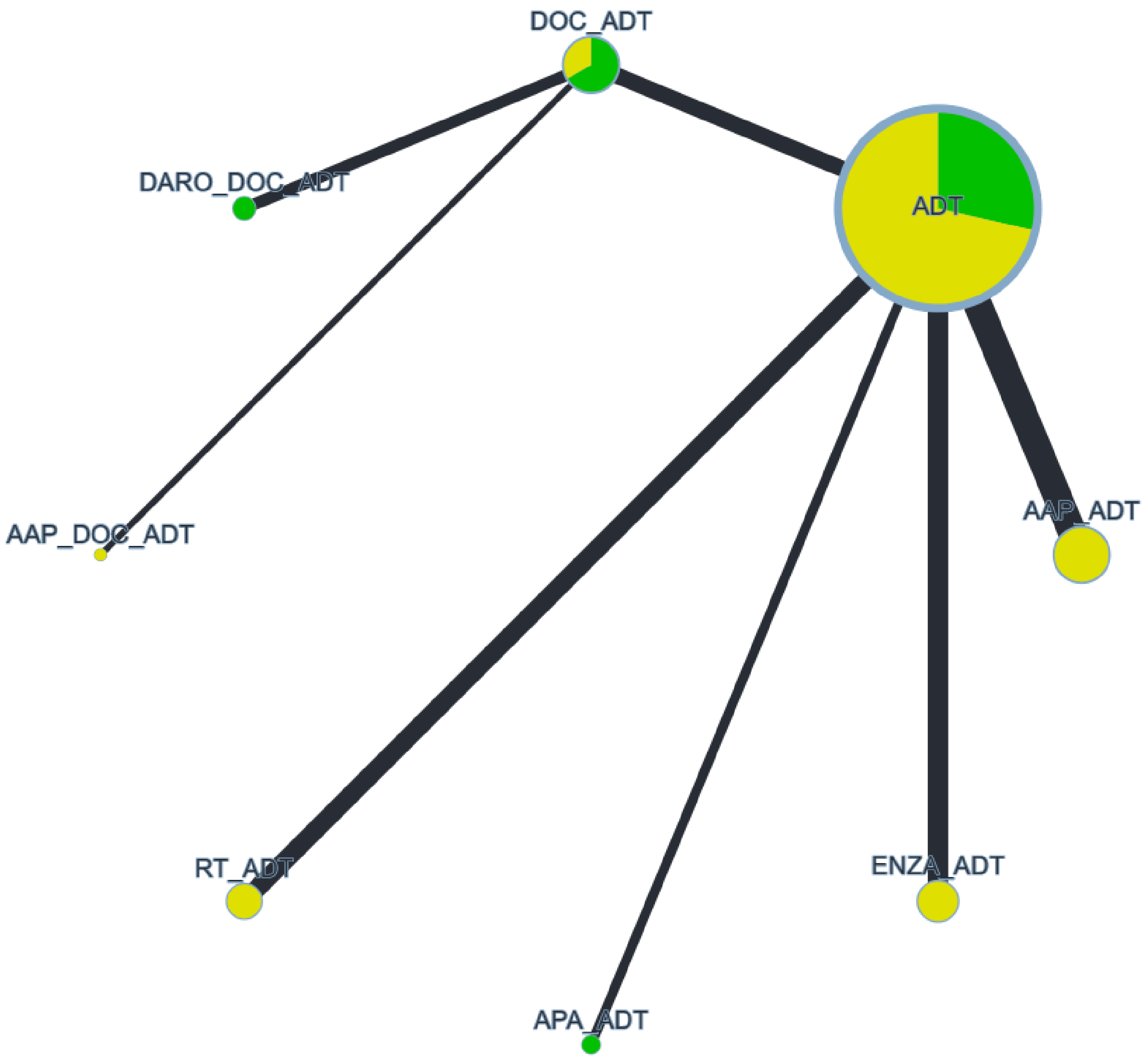

6.2 Risk of Bias Chart showing the contribution of low, moderate or high RoB comparisons to each network estimate

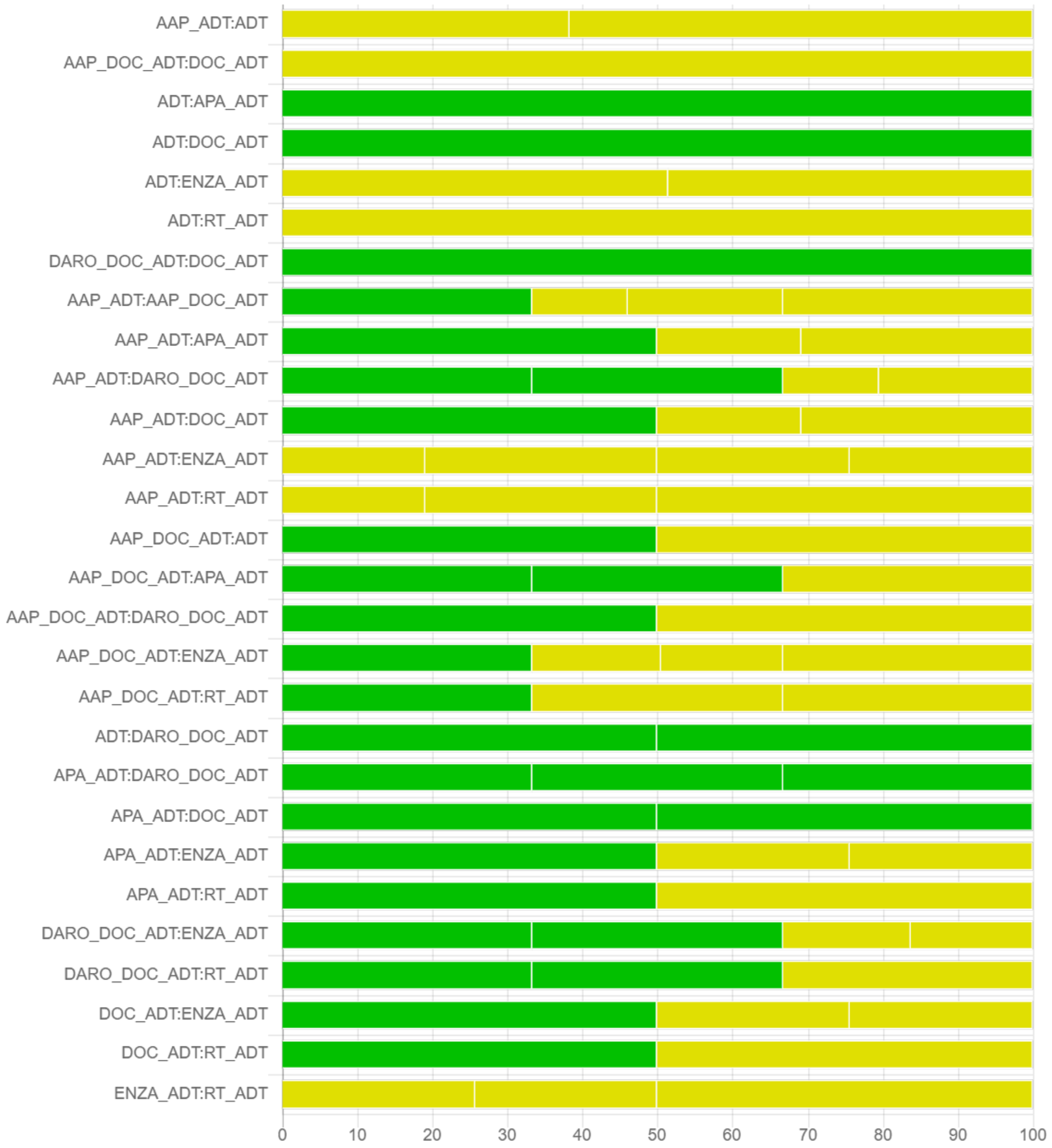

6.3 Evaluation of the Certainty of evidence Using CINEMA Framework, mixed evidence

Comparison	Number of studies	Within-study bias	Reporting bias	Indirectness	Imprecision	Heterogeneity	Incoherence	Confidence rating
AAP_ADT:ADT	2	Some concerns	Low risk	No concerns	No concerns	Major concerns	Major concerns	Very low
AAP_DOC_ADT:DOC_ADT	1	Some concerns	Low risk	No concerns	No concerns	Major concerns	Major concerns	Very low
ADT:APA_ADT	1	No concerns	Low risk	No concerns	No concerns	Major concerns	Major concerns	Very low
ADT:DOC_ADT	1	No concerns	Low risk	No concerns	No concerns	Major concerns	Major concerns	Very low
ADT:ENZA_ADT	2	Some concerns	Low risk	No concerns	No concerns	Major concerns	Major concerns	Very low
ADT:RT_ADT	1	Some concerns	Low risk	No concerns	No concerns	Major concerns	Major concerns	Very low
DARO_DOC_ADT:DOC_ADT	1	No concerns	Low risk	No concerns	Some concerns	Some concerns	Major concerns	Very low
AAP_ADT:AAP_DOC_ADT	0	Some concerns	Low risk	No concerns	No concerns	Major concerns	Major concerns	Very low
AAP_ADT:APA_ADT	0	Some concerns	Low risk	No concerns	No concerns	Major concerns	Major concerns	Very low
AAP_ADT:DARO_DOC_ADT	0	No concerns	Low risk	No concerns	Some concerns	Some concerns	Major concerns	Very low
AAP_ADT:DOC_ADT	0	Some concerns	Low risk	No concerns	Some concerns	Some concerns	Major concerns	Very low
AAP_ADT:ENZA_ADT	0	Some concerns	Low risk	No concerns	Some concerns	Some concerns	Major concerns	Very low
AAP_ADT:RT_ADT	0	Some concerns	Low risk	No concerns	No concerns	Major concerns	Major concerns	Very low
AAP_DOC_ADT:ADT	0	Some concerns	Low risk	No concerns	No concerns	Major concerns	Major concerns	Very low
AAP_DOC_ADT:APA_ADT	0	No concerns	Low risk	No concerns	No concerns	Major concerns	Major concerns	Very low
AAP_DOC_ADT:DARO_DOC_ADT	0	Some concerns	Low risk	No concerns	Some concerns	Some concerns	Major concerns	Very low
AAP_DOC_ADT:ENZA_ADT	0	Some concerns	Low risk	No concerns	No concerns	Major concerns	Major concerns	Very low
AAP_DOC_ADT:RT_ADT	0	Some concerns	Low risk	No concerns	No concerns	Major concerns	Major concerns	Very low
ADT:DARO_DOC_ADT	0	No concerns	Low risk	No concerns	No concerns	Major concerns	Major concerns	Very low
APA_ADT:DARO_DOC_ADT	0	No concerns	Low risk	No concerns	No concerns	Major concerns	Major concerns	Very low
APA_ADT:DOC_ADT	0	No concerns	Low risk	No concerns	No concerns	Major concerns	Major concerns	Very low
APA_ADT:ENZA_ADT	0	Some concerns	Low risk	No concerns	Some concerns	Some concerns	Major concerns	Very low
APA_ADT:RT_ADT	0	Some concerns	Low risk	No concerns	No concerns	Major concerns	Major concerns	Very low
DARO_DOC_ADT:ENZA_ADT	0	No concerns	Low risk	No concerns	No concerns	Major concerns	Major concerns	Very low
DARO_DOC_ADT:RT_ADT	0	No concerns	Low risk	No concerns	No concerns	Major concerns	Major concerns	Very low
DOC_ADT:ENZA_ADT	0	Some concerns	Low risk	No concerns	Some concerns	Some concerns	Major concerns	Very low
DOC_ADT:RT_ADT	0	Some concerns	Low risk	No concerns	No concerns	Major concerns	Major concerns	Very low
ENZA_ADT:RT_ADT	0	Some concerns	Low risk	No concerns	No concerns	Major concerns	Major concerns	Very low

The end.
